# Synchrony is more than its top-down and climatic parts: interacting Moran effects on phytoplankton in British seas

**DOI:** 10.1371/journal.pcbi.1006744

**Published:** 2019-03-28

**Authors:** Lawrence W. Sheppard, Emma J. Defriez, Philip C. Reid, Daniel C. Reuman

**Affiliations:** 1 Department of Ecology and Evolutionary Biology and Kansas Biological Survey, University of Kansas, Lawrence, Kansas, USA; 2 Department of Life Sciences, Imperial College London, Ascot, United Kingdom; 3 Marine Institute, Plymouth University, Drake Circus, Plymouth, United Kingdom; 4 Marine Biological Association of the UK, The Laboratory, Citadel Hill, Plymouth, United Kingdom; 5 Department of Ecology and Evolutionary Biology and Kansas Biological Survey, University of Kansas, Lawrence, Kansas, USA; 6 Laboratory of Populations, Rockefeller University, New York, New York, USA; UC Davis, UNITED STATES

## Abstract

Large-scale spatial synchrony is ubiquitous in ecology. We examined 56 years of data representing chlorophyll density in 26 areas in British seas monitored by the Continuous Plankton Recorder survey. We used wavelet methods to disaggregate synchronous fluctuations by timescale and determine that drivers of synchrony include both biotic and abiotic variables. We tested these drivers for statistical significance by comparison with spatially synchronous surrogate data. Identification of causes of synchrony is distinct from, and goes beyond, determining drivers of local population dynamics. We generated timescale-specific models, accounting for 61% of long-timescale (> 4yrs) synchrony in a chlorophyll density index, but only 3% of observed short-timescale (< 4yrs) synchrony. Thus synchrony and its causes are timescale-specific. The dominant source of long-timescale chlorophyll synchrony was closely related to sea surface temperature, through a climatic Moran effect, though likely via complex oceanographic mechanisms. The top-down action of *Calanus finmarchicus* predation enhances this environmental synchronising mechanism and interacts with it non-additively to produce more long-timescale synchrony than top-down and climatic drivers would produce independently. Our principal result is therefore a demonstration of interaction effects between Moran drivers of synchrony, a new mechanism for synchrony that may influence many ecosystems at large spatial scales.

## Author summary

The size of the annual bloom in phytoplankton can vary similarly from year to year in different parts of the same oceanic region, a phenomenon called *spatial synchrony*. The growth of phytoplankton near the ocean surface is the foundation of marine food webs, which include numerous commercially exploited species. And spatial synchrony in phytoplankton abundance time series can have consequences for the total production of marine ecosystems. Therefore we studied the spatial synchrony of fluctuations in green phytoplankton abundance in 26 areas in seas around the British Isles. Variation and synchrony can occur differently on long and short timescales. We used a novel wavelet-based approach to examine long- and short-timescale fluctuations separately, and we thereby show that slow synchronous fluctuations in phytoplankton can be explained by the effects of slow synchronous fluctuations in sea surface temperature and related oceanographic phenomena, and by the effects of synchronous fluctuations in a zooplankton predator. Crucially, these drivers reinforce one another in a super-additive way, the interaction constituting a new mechanism of synchrony. Future changes in the climate or changes in predation are likely to influence phytoplankton synchrony via this mechanism and hence may influence the aggregate productivity of British seas.

## Introduction

Many ecosystems are subject to large spatially synchronous fluctuations, with serious consequences for ecosystem services and stability across space and time. Spatial synchrony is the tendency for spatially separated populations to undergo correlated fluctuations. Spatial synchrony is a fundamental and nearly ubiquitous feature of ecological population dynamics, having been observed in thousands of species from a wide variety of taxa [1].

It is well known that spatially synchronous fluctuations in population abundance can result from spatially synchronous fluctuations in the environment; this is called the Moran effect [2]. For example correlated fluctuations in the abundances of spatially separated coral reef fish populations were found to be related to spatially extensive climatic fluctuations related to the ENSO index [3]. A major challenge has been to identify the environmental drivers acting in real systems and thereby to demonstrate the Moran effect in action [4, 5]. Cases in which individual environmental drivers such as precipitation or temperature fluctuations have been statistically identified as Moran-type synchronizers are emerging [6, 7]. Steen *et al*. suggest the Moran effect is generally more important than dispersal as a factor synchronizing population fluctuations [8].

But synchronizing mechanisms can be complex. Defriez *et al*. identify multiple potential environmental drivers of synchrony in terrestrial vegetation growth [9], specifically temperature and precipitation. Multiple Moran drivers may, in principle, show interaction effects. Kendall *et al*. suggested theoretically that dispersal and Moran effects do not combine in a merely additive way [10], i.e., population synchrony in their models differed from what would be expected by simply adding the synchronizing effects of dispersal and environmental correlation. Even considering only Moran effects, in addition to synchronous environmental forcing (here referred to as a ‘climatic’ driver of synchrony, or as a ‘climatic’ Moran effect), populations may also be subject to biotic factors such as spatially synchronous predation (here considered a ‘top-down’ driver of synchrony, included for our purposes under the term ‘Moran effect’). Spatial synchrony in a given species may thus be the result of a complex of interacting factors.

To illustrate the theoretical potential for interaction effects between Moran drivers, consider the simple case of a population index *γ*(*n*, *t*) following an autoregressive (AR1) process in locations *n* = 1, 2 at times *t*. By the Moran theorem [2] the correlation of the population between the two locations is the correlation of the environmental drivers, which is equal to their covariance normalized by the geometric mean of their variances. The Moran theorem is a statement about correlations of stochastic processes. These correlations can be estimated from simulations or sampled data, but sampling error and transients make such estimation inexact for short time series. Real ecological data does not, in general, arise from autoregressive processes with only one driver. Moran’s original formulation includes only a single spatially synchronous driver, but a model including a second driver makes clear the importance of any relationship between the drivers to the resultant ecological synchrony. If the environmental drivers are each a weighted sum of variables *α*(*n*, *t*) and *β*(*n*, *t*), then the numerator and denominator of their correlation now depend not only on covariances between locations of *α*, and likewise for *β*, but also on covariances (interactions) between these drivers. See Appendix S1 of Supporting Information for mathematical working.

The planktonic ecosystem is the foundation of the oceanic food web and of commercial fisheries. And it is also an exemplar of a complex ecological network within which any species may, in principle, be subject to numerous synchronizing influences which may interact as indicated above. Several factors affect phytoplankton growth, including CO_2_, temperature, and pH [11]; each of these has the potential to promote synchrony via a Moran effect. Zooplankton predation and a suite of interrelated oceanographic phenomena are also of well known importance [12]. Sea surface temperature relates to water column mixing and critical-layer depth via complex mechanisms that can drive phytoplankton synchrony [13–15]. Plankton growth is highly ‘patchy’ [16], but, nevertheless, at a regional scale plankton communities show coherent and persistent patterns of change through time [17–19]. The examination of variability on long spatial and temporal scales may illuminate causes of plankton synchrony and patches. Plankton make an excellent system in which to investigate the possible importance of multiple and possibly interacting Moran drivers.

In this paper we examine annualized plankton abundance data in the sea around the British Isles, drawn from the Continuous Plankton Recorder (CPR) survey of the Marine Biological Association (MBA) of the United Kingdom, in order to understand the possibly complex influences acting to spatially synchronize phytoplankton abundance. Until recently, the CPR survey was run by the Sir Alister Hardy Foundation for Ocean Science, SAHFOS. The CPR data includes a Phytoplankton Color Index (PCI) which undergoes spatially correlated fluctuations [20], and which we analyse. Raitsos *et al*. [21] have calibrated this index with chlorophyll fluorescence and show [22] that it correlates well with satellite measures of ocean color. The PCI is a good indicator of the presence of chlorophyll and primary production in the North Sea [23, 24].

Phytoplankton abundance undergoes fluctuations with varying timescales at different times, complicating the identification of Moran effects and interactions between Moran effects; this is a general feature of spatiotemporal ecological data. Monthly time series were compiled but ‘missing months’ when no data were available required filling with median values for each site and month (Methods). The monthly data were dominated by the seasonal phytoplankton bloom and seasonal changes in all the covariates, with a fixed one-year period. Seasonal plankton dynamics have been well studied. Our examination of annualized data instead gives information about the aggregate effect of drivers on fluctuations in the bloom size from year to year, allowing us to identify coherent inter-year fluctuations with statistical confidence. Sheppard *et al*. show that synchrony in aphid populations across the United Kingdom (UK) changed substantially from before to after about 1993, shifting from long-timescale-dominated to short-timescale-dominated synchrony in response to a shift in the North Atlantic Oscillation [7]. Via an approach that resolved patterns on different timescales, Defriez *et al*. showed the synchrony of fluctuating plankton populations in the North Sea changed significantly from before the 1980s to after the 1980s [20]. A regime shift in the abundances of various plankton species occurred in the 1980s [25–27] including an overall increase in the PCI and a reduction in *C. finmarchicus* abundance. Research continues to emerge showing the importance of the timescale structure of population dynamics to understanding synchrony [7, 9, 15, 20, 28–30].

Another complicating factor for identifying Moran drivers for plankton is the likelihood of relationships other than strict proportionality between fluctuations in a driver variable and the fluctuations induced in plankton growth, including possibly time-lagged relationships. Liebhold *et al*. suggest phase-coherence-based techniques as a way of identifying relationships with a delay or phase shift [1]. Pioneering work by Colebrook [31] using Fourier techniques demonstrates a ‘phase-shifted’ relationship between the fluctuations of North Sea plankton abundance and sea surface temperature.

We earlier demonstrated [7] how wavelet techniques were effective in accounting for the synchrony of aphid fluctuations, in spite of complexities of biological systems such as those listed above. We here build on our earlier timescale- and phase-sensitive wavelet methods and apply the improved methods to fluctuations in phytoplankton abundance. Among the wavelet approaches in our earlier work, we developed a wavelet version of the classic Moran theorem that made it possible to quantify the proportion of observed synchrony attributable to a given Moran driver at a given timescale. We also developed an approach based on examining the statistics of spatially synchronous artificial time series (surrogate data) to demonstrate that observed wavelet relationships between empirical data represented statistically significant associations. The wavelet transform (see [32] for general background) of a plankton abundance time series *x*(*t*) (*t* = 1, …, *T*) is a complex number *W*_*σ*_(*t*) with magnitude and phase that give, respectively, the strength and phase of the oscillations in *x*(*t*) of timescale/period *σ* at time *t*. The *frequency* of a periodic fluctuation is henceforth identified as the reciprocal of its timescale, *σ*.

Because of the complexity of the plankton system, understanding phytoplankton synchrony requires the development of statistical wavelet models incorporating the effects of more than one driver, and exploration of the effects on the synchrony expected when these drivers either reinforce or counteract one another. We apply a timescale specific fitting procedure to represent the fluctuations of the plankton in terms of K drivers,

$$\begin{array}{c} w_{\sigma }^{\left(\text{phytoplankton}\right)}\left(t\right)\approx \beta_{1}\left(\sigma \right)w_{\sigma }^{\left(\text{driver}\;\text{1}\right)}\left(t\right)+\cdots +\beta_{K}\left(\sigma \right)w_{\sigma }^{\left(\text{driver}\;K\right)}\left(t\right), \end{array}$$ (1)

where the *w*_*σ*_(*t*) are normalizations (rescalings by a constant) of the wavelet transforms, *W*_*σ*_(*t*) (details below). The use of complex *β*(*σ*) coefficients allows for lagged effects (phase shifts) of drivers on phytoplankton, as well as variable strengths of effects depending on the timescale of fluctuation. For example, a negative correlation between PCI and *C. finmarchicus* consumers is described by Beaugrand *et al*. [33]: this is equivalent to a half-cycle phase shift.

Our specific research goals are: 1) to determine which physical and ecological variables are associated with PCI fluctuations at long and short timescales and therefore probably help cause synchrony in PCI through Moran effects; 2) to determine how much of the synchrony observed at long and short timescales in PCI can be explained statistically by the collective influence of these factors; 3) to explore possible interaction effects among the distinct Moran influences, i.e., to explore whether the factors significantly reinforce or counteract each other (in a non-additive manner) in causing PCI synchrony. If detected, interaction effects among Moran drivers would be a newly recognized mechanism of synchrony. We emphasize that our primary goal here is to understand the causes of spatial synchrony in the PCI, which is distinct from explaining local dynamics of the PCI, a previously much investigated topic. To accomplish our goals, we have developed new wavelet methods that may be widely applicable. Since some of the factors we consider are environmental and some comprise the influence of herbivorous zooplankton, our explorations constitute an examination of ‘top-down’ and ‘climatic’ Moran effects, and possible interactions between these effects.

## Results

### Theoretical illustration that enhanced synchrony can result from reinforcement between Moran drivers

**Fig 1:**
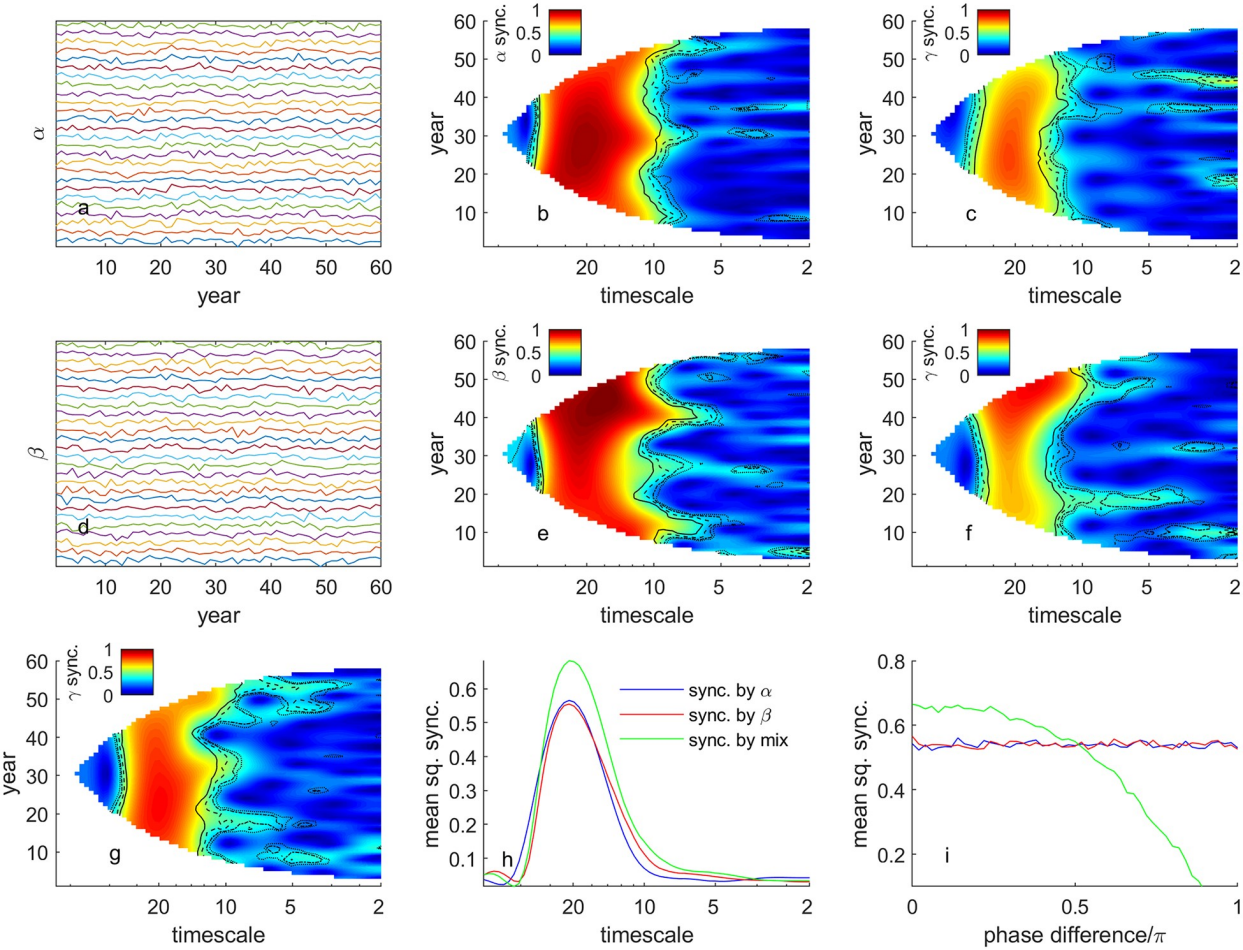
Synthetic example showing the effects of two different synchronizing factors, individually and in interactive combination. Panel (a) shows fluctuations in 26 signals *α*(*n*, *t*) with a synchronous 20-year-period sine wave component obscured by independent local noise. Panel (d) shows 26 signals *β*(*n*, *t*) with a synchronous 20-year-period cosine component, also obscured by independent local noise of the same strength. Panels (b) and (e), which are wavelet mean field magnitudes (WMFM; Methods) of (a) and (d), respectively, display synchrony as a function of time and timescale and reveal synchrony at timescale 20 years. Panels (c) and (f) show WMFMs of populations influenced by *α* and *β*, respectively, and also by independent local noise time series of the same strength. *β* acts with a 5-year lag, 1/4 of the underlying 20-year periodicity. Synchrony is still visible, and is similar in strength, but is muted relative to (b) and (e) by the additional local noise acting on the populations. Panel (g) shows the WMFM of populations subject to a mixed influence of *α* and *β*, normalized so this influence had the same variance through time as the influences *α* and *β* had individually for panels (c) and (f). Independent local noise of the same strength was again applied. Synchrony is stronger in (g) than in (c) and (f), demonstrating interaction effects of the synchronizing agents *α* and *β*. Panel (h) shows the time-averaged square of the WMFM of (g), in green (called the mean squared WMFM or mean squared synchrony in Methods), which is greater than that of (c), in blue, or (f), in red. Panel (i) shows how interaction effects are a result of the phase relationships between the drivers. As the phase of the co-sinusoids underlying *β* are modified in further simulations, the interaction effect goes from positive to negative as the phase shift passes 0.5*π*. Significance contours on Fig.1b,c,e,f,g represent wavelet phasor mean field magnitude (Methods; WPMFM) significance thresholds at the 0.1, 0.05, 0.01, and 0.001 levels, respectively for the dot, dot-dash, dash and line contours. These significance thresholds are relative to a null hypothesis of no association between the phases of the 26 transforms. See text for mathematical details. WMFMs are not guaranteed to be ≤ 1 at all times, but ours were except for (e), which had maximum squared value 1.0111. For clearer plotting, we reassigned values > 1 in (e) to 1. Sync. = synchrony.

We constructed a numerical example including frequency-specific synchrony arising from multiple drivers, as a conceptual illustration and for use in verifying the effectiveness of statistical methods to be used on data. The example is described below. By construction, the example includes two drivers of synchrony that can either reinforce or counteract one another in the same way as the drivers of PCI synchrony identified below. Thus the example also serves a pedagogical purpose by introducing, in simplified form, the kinds of relationships we later show to hold for plankton in British seas. The example makes use of phase-shifted relationships between variables and timescale-specific synchrony, and shows explicitly how interaction effects can increase or decrease synchrony. The approach also illustrates the use of the wavelet tools we apply to empirical data. Our example is demonstrated principally through Fig 1. By way of overview, Fig 1a, 1b and 1c show an artificial spatiotemporal ‘driver’, a wavelet measure of its synchrony, and the resulting synchrony of a ‘driven’ variable, respectively (details below). Fig 1d, 1e and 1f show a different driver, its synchrony, and the resulting synchrony of a second driven variable, respectively. Fig 1g, 1h and 1i describe the synchrony of a third driven variable that depends on both drivers.

For sampling locations indexed by *n* and sampling times indexed by *t*, define the (biotic or abiotic) environmental random variable (Fig 1a) $\alpha \left(n,t\right)=\sqrt{\frac{1}{4}}\alpha_{S}\left(n,t\right)+\sqrt{\frac{3}{4}}\alpha_{L}\left(n,t\right)$, where $\alpha_{S}\left(n,t\right)=\sqrt{2}\sin\left(wt\right)$ is the ‘synchronous component’ of *α*(*n*, *t*) and *α*_*L*_(*n*, *t*) is a ‘local noise’. This and other local noise terms will be considered to be standard-normally distributed for all *n* and *t*, and independent through time *t*, across space *n*, and of other local noises. We take *w* = 2*π*/20, so the timescale of oscillations we consider in this example is 20 years. We use *n* = 1, …, 26 and *t* = 0, …, 59 in this example because these values are similar to the analogous values in the empirical data we study below. It is straightforward to show that $\sqrt{\frac{1}{4}}\alpha_{S}$ contributes 1/4 and $\sqrt{\frac{3}{4}}\alpha_{L}$ contributes 3/4 of the variance through time of *α*, so that the ‘combination ratio’ of the synchronous and local-noise components of *α* is 1:3. Likewise define the environmental variable $\beta \left(n,t\right)=\sqrt{\frac{1}{4}}\beta_{S}\left(n,t\right)+\sqrt{\frac{3}{4}}\beta_{L}\left(n,t\right)$ (Fig 1d), where we set $\beta_{S}\left(n,t\right)=\sqrt{2}\cos\left(wt-\varphi \pi \right)$ for *φ* a constant, and *β*_*L*_(*n*, *t*) is another local noise. Again, the combination ratio of the synchronous and local-noise components is 1:3. We initially consider the case *φ* = 0, later relaxing this assumption.

The *wavelet mean field magnitude* (WMFM; Methods) is a statistical technique that takes spatiotemporal data such as *α*(*n*, *t*) and provides a plot that shows how much synchrony exists between these time series for each time and timescale of analysis [7]. The WMFM approach picks out the synchronized fluctuations in *α* (Fig 1b) and in *β* (Fig 1e) as bright features at timescale 20 years spanning the duration of the time series, introducing the WMFM technique by way of demonstration.

Three alternative population-dynamical variables *γ*^(*i*)^(*n*, *t*) for *i* = 1, 2, 3 are now constructed, each influenced by different combinations of *α* and *β*. Each variable *α* and *β* is partially spatially synchronous, and can thus have a synchronizing effect on *γ*^(*i*)^. We will imagine *α* represents a top-down influence such as predation, and *β* represents a climatic influence. Depending on the causal mechanism, the two signals *α* and *β* may bear different phase relationships to the fluctuations they produce in a population variable *γ*. For example a low value of *α* may tend to produce high values of *γ*, yielding a negative correlation and a *π* phase difference between their fluctuations, whereas the effect of *β* may be subject to a time delay introducing a different phase shift. We define $\gamma^{\left(i\right)}\left(n,t\right)=\sqrt{\frac{1}{4}}\gamma_{S}^{\left(i\right)}\left(n,t\right)+\sqrt{\frac{3}{4}}\gamma_{L}^{\left(i\right)}\left(n,t\right)$, where the $\gamma_{L}^{\left(i\right)}$ are local noise, $\gamma_{S}^{\left(1\right)}\left(n,t\right)=-\alpha \left(n,t\right)$, $\gamma_{S}^{\left(2\right)}\left(n,t\right)=-\beta \left(n,t-5\right)$, and $\gamma_{S}^{\left(3\right)}\left(n,t\right)=-\left(\alpha \left(n,t\right)+\beta \left(n,t-5\right)\right)/f$, where $f=\sqrt{2+\frac{1}{2}\cos\left(\varphi \pi \right)}$ is chosen so that the variance through time of $\gamma_{S}^{\left(3\right)}\left(n,t\right)$ is 1, the same as the variance through time of $\gamma_{S}^{\left(1\right)}$ and $\gamma_{S}^{\left(2\right)}$ (Appendix S1). Thus in all three cases the partially synchronous components $\gamma_{S}^{\left(i\right)}\left(n,t\right)$ are combined with the local noise components $\gamma_{L}^{\left(i\right)}\left(n,t\right)$ in combination ratio 1:3. The negative influence of *α* on *γ*^(1)^ will produce a negative correlation between these variables. The 5-year lag of the effects of *β* on *γ*^(2)^ is a quarter of the 20-year cycle built into *β* (*π*/2 radians phase difference). The *γ*^(3)^ example models a combined influence of top-down (*α*) and climatic (*β*) Moran effects, though the strength of the combined effect is kept equal to the strength of the individual top-down and climatic effects acting, respectively, on *γ*^(1)^ and *γ*^(2)^ through *α* and *β*. Thus effects can be fairly compared across our three population models.

WMFMs of the *γ*^(*i*)^ reveal that synchrony is transmitted to all three population variables, but that, for *φ* = 0, the synchrony in *γ*^(3)^ was stronger than that in *γ*^(1)^ and *γ*^(2)^, revealing interaction effects between Moran drivers. The WMFMs of *γ*^(1)^ (Fig 1c) and *γ*^(2)^ (Fig 1f) show that the influence of *α* and *β*, respectively, have synchronized these population variables at the 20-year timescale. Synchrony in the populations is less than in the driver variables, as expected since the populations are also influenced by local noise. The WMFM of *γ*^(3)^ (Fig 1g) also shows synchrony at 20-year timescale, and this synchrony is stronger than that of *γ*^(1)^ and *γ*^(2)^. Time-averaged squared WMFMs (Fig 1h) clarify the comparison of strengths of synchrony of the *γ*^(*i*)^. Because of the *π*/2 phase relationship between sine (in *α*_*S*_) and cosine (in *β*_*S*_) when *φ* = 0, and the *π*/2-phase-lagged effects of *β* on *γ*^(3)^, the synchronizing influences of *α* and *β* on *γ*^(3)^ reinforce one another. The asynchronous variations in *α* and *β* (local noise) tend to cancel out when these drivers are combined, leaving their synchronous components to have relatively larger influence.

We investigated interaction effects between Moran drivers further by considering *φ* ≠ 0. When *φ* ≠ 0, the effects of *α*_*S*_ and the lagged effects of *β*_*S*_ on *γ*^(3)^ no longer perfectly reinforce each other. For *φ* increasing from 0 to 0.5, reinforcement is partial, and positive interaction effects are seen; for *φ* increasing further from 0.5 to 1, negative interactions are seen (Fig 1i). Fig 1i shows typical (average over 50 realisations) time-averaged squared mean field magnitude values for *γ*^(*i*)^ at 20-year timescale for *i* = 1, 2, 3 in blue, red, and green, respectively. The crossover threshold of *φ* = 0.5 between the green and red or blue lines on Fig 1i corresponds to the fact that quarter-cycle-shifted sinusoids (*π*/2 phase shift) have correlation 0 with each other.

### PCI fluctuations and synchrony have both top-down and climatic causes

At long timescales (> 4 years), the best wavelet model of PCI according to our model selection and testing procedure (Methods), which was based on wavelet regression and out-of-sample cross validation, included two predictor variables: *C. finmarchicus* abundance and sea surface temperature during the growing season. Models considered in the model selection procedure were constructed using combinations of the predictors listed in Tables 1 and 2. Other high-ranked models were similar, but not identical to the top model (S1 Table): all included *C. finmarchicus* and either growing season or annual average temperature; summer salinity and autumn cloud cover were included as predictors in some models. For simplicity and because all of the top-ranking models included *C. finmarchicus* and a temperature variable, and because other variables were only included in some of the top-ranking models, subsequent analyses focussed on the one top model. At short timescales (< 4 years) the best model included *C. finmarchicus* abundance, decapod larvae abundance, and echinoderm larvae abundance. Even the best model at short timescales performed poorly for our purpose of explaining synchrony (see below), so other short-timescale models were not listed.

**Table 1:** The names of the plankton variables investigated. Each time series was constructed by averaging monthly values over all twelve months, to produce one value per year per location.

*Calanus* I-IV
*Para-Pseudocalanus* spp.
*Acartia* spp. (unidentified)
*Oithona* spp.
*Pseudocalanus elongatus* adult
*Temora longicornis*
*Centropages typicus*
*Calanus finmarchicus*
*Calanus helgolandicus*
*Metridia lucens*
Echinoderm larvae
Decapoda larvae (total)
Euphausiacea (total)

**Table 2:** The names given to the environmental variables investigated. Each time series was constructed by averaging monthly values over the relevant months, to produce one value per year per location.

Variable	Months averaged
Yearly temperature	1 to 12
Spring temperature	3 to 5
Summer temperature	6 to 8
Autumn temperature	9 to 11
Growing season temperature	3 to 9
Yearly wind speed	1 to 12
Spring wind speed	3 to 5
Summer wind speed	6 to 8
Autumn wind speed	9 to 11
Growing season wind speed	3 to 9
Yearly salinity	1 to 12
Spring salinity	3 to 5
Summer salinity	6 to 8
Autumn salinity	9 to 11
Growing season salinity	3 to 9
Yearly cloud cover	1 to 12
Spring cloud cover	3 to 5
Summer cloud cover	6 to 8
Autumn cloud cover	9 to 11
Growing season cloud cover	3 to 9

Two timescale bands (<4 years and >4 years), as opposed to more bands, were used for simplicity and because wavelet methods have finite ability to resolve timescales of fluctuation in finite time series. The practical distinction between short (<4 year) and long (>4 year) timescale bands follows the same reasoning as [7]: considering lag-1 autocorrelation, a time series (or a spatially synchronous component thereof) dominated by >4-year components of variability will demonstrate slow changes in the sense that successive years will typically be positively correlated. If dominated by <4-year components, the correlation will be negative. The 4-year timescale is the boundary between persistence and anti-persistence in the ecological data.

Each of the variables included in the best models at short and long timescales, respectively, were tested for statistically significant spatial coherence (Methods) with PCI when controlling for other variables in the model. In other words, normalized predictor transforms $w_{n,\sigma }^{\left(k\right)}\left(t\right)$ included in a model were required to provide a significant improvement in model explanatory power over the model with that predictor removed (Methods). For long-timescales, the *p*-value associated with removing *C. finmarchicus* from the top-ranked model was 0.0023, and the *p*-value associated with removing growing season temperature was 0.0019. For short timescales, the *p*-values associated with separate removals of *C. finmarchicus*, decapod larvae, and echinoderm larvae abundance, respectively, from the top-ranked model were 0.0034, 0.0361, and 0.0017. Our best models were also tested and validated in several additional ways (Methods).

We extracted the phases of the *β*_*k*_ coefficients in our best fit models to determine the lag between the fluctuations in the *k*th predictor and the corresponding content in the PCI wavelet transforms. See Methods for details and S1 Fig for a plot of phase differences. A quarter-cycle phase shift existed between the long-timescale driver, temperature, and PCI, with high PCI values following low temperature values. Across the range of timescales in the long-timescale band, the phase of PCI leads the phase of temperature with coefficient phases between 0.5 and 2.1 radians. *C. finmarchicus* was found to be in antiphase with PCI at all frequencies, indicating an association between high *C. finmarchicus* abundance and low PCI. In the long-timescale band, the phase of PCI leads the phase of *C. finmarchicus* with coefficient phases between 2.8 and 4.2 radians; and in the short-timescale band between 3.0 and 3.5 radians. The two other short-timescale predictors, decapod larvae and echinoderm larvae, were in phase with the fluctuations in PCI. In the short-timescale band, decapod larvae had coefficient phases between 0 and 0.4 radians, and echinoderm larvae had coefficient phases between -0.4 and 0 radians.

The drivers and their phase relationships to PCI at low frequencies correspond to those illustrated in Fig 1. PCI maintains an antiphase relationship with *C. finmarchicus* abundance just as *γ*^(1)^ does with driver *α*, and a quarter cycle phase shift with the temperature variable just as *γ*^(2)^ does with driver *β*. We can consider *C. finmarchicus* a top-down driver of PCI fluctuations, as the anti-phase relationship between the variables, which pertains even after including temperature in the model, implies variation in PCI driven by predation (see Discussion for further elaboration). As in Fig 1i, the resultant mean squared WMFM of PCI will depend on the interaction between the effects of these drivers, whether further synchronizing or desynchronizing, as investigated below.

Returning to short timescales, the in-phase relationships between PCI and echinoderm and decapod larvae may reflect environmental or other influences that impact these variables in similar ways. Reid and Beaugrand proposed that the larger phytoplankton production reflected in the growth in the PCI index that followed the 1980s North Sea regime shift increased sedimentation of planktonic detritus to the benthos leading to a cascade of ecological responses in the pelagos and benthos [19]. Kirby *et al*. and Lindley *et al*. showed that decapods and echinoderms responded to the climatic regime shift with a marked increase in abundance of both the benthic adult stage and their planktonic larvae [34, 35].

### Fractions of synchrony explained

Having just demonstrated a dual climatic and top-down Moran effect on PCI, we now explore the proportion of PCI synchrony this effect can explain.

**Fig 2:**
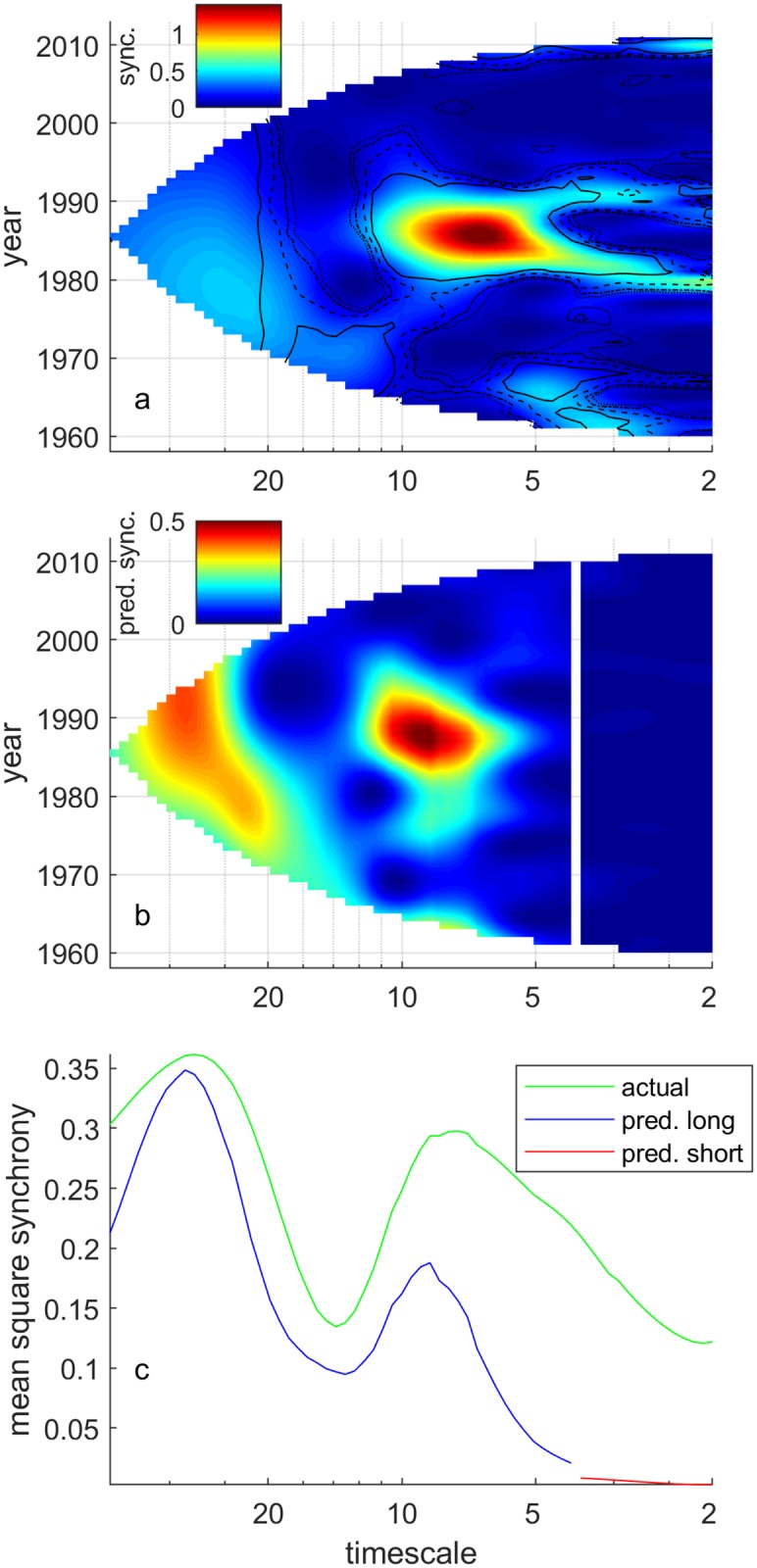
Our best long-timescale model explained synchrony on long timescales, but our short-timescale model did not explain synchrony on short timescales. The PCI squared WMFM plot (a, colors) showed that spatially synchronous fluctuations in PCI occurred at different time points within the time series for different frequencies. Contours indicate statistically significant phase synchrony (at the 0.1, 0.05, 0.01, and 0.001 levels, respectively for the different contours) and are taken from the WPMFM plot (Methods; S2 Fig). Synchrony predicted (Methods) by the best long-timescale wavelet model (b, left of white line) resembled real patterns of synchrony, but synchrony predicted by the best short-timescale model (b, right of white line) did not. Mean squared WMFM (c, green line; Methods) was moderately well approximated by the mean squared value of predicted synchrony for long timescales (c, blue line) but not for short timescales (c, red line). Sync. = synchrony; pred. = predicted.

The WMFM plot for PCI (Fig 2a, colors) showed that synchrony (high WMFM) in PCI occurred at different time points within the time series for different frequencies. The wavelet phasor mean field magnitude plot (WPMFM; Methods) for PCI (S2 Fig; significance contours from that figure are superimposed on the WMFM plot of Fig 2a) showed that synchrony was significant for some times and timescales and not for others. Here the WMFM is being used to display synchrony and the WPMFM is being used to test for significance of phase synchrony. Significance testing only the phase associations highlights significantly synchronous features in the transforms without requiring a particular null hypothesis about the site-specific variability in the transforms (Methods). The significance contours of the WPMFM were constructed and are interpretable in the same way as for the simulated time series in Fig 1. Synchrony is especially apparent in the WMFM plot on long timescales, and in a feature in the mid 1980s. A ‘regime shift’ in the North Sea plankton community in the 1980s has been well documented (e.g., [26, 36]), and is likely to be associated with this feature.

Our wavelet Moran theorem (Methods) led to model-predicted synchrony plots and fractions of synchrony explained that showed that our best models explained PCI synchrony effectively on long timescales, but poorly on short timescales. Model-predicted synchrony plots have the same format as WMFM plots but represent the synchrony that would have occurred if the only synchronizing agents were those predictor variables included in the model (Methods; this is $|\Pi_{\sigma }^{\left(0h\right)}\left|\right|r_{\sigma }^{\left(h\right)}\left(t\right)|$ in the notation used there). The model-predicted synchrony plot based on our best long-timescale model (Fig 2b, left of the white line) resembled the PCI WMFM plot (Fig 2a) for long timescales. In particular, a feature in the model-predicted synchrony plot (Fig 2b) representing high synchrony on the longest timescales examined (> 20 years) corresponds to a similar peak in the observed PCI WMFM plot; note the different color bars on the two panels when making this comparison. Synchrony is also high on both plots at timescales of around 8 years in the 1980s. In contrast, the model-predicted synchrony plot based on our best short-timescale model (Fig 2b, right of white line) bore no resemblance to the PCI WMFM plot (Fig 2a) for short timescales. Correspondingly, the mean squared WMFM or mean squared synchrony (Fig 2c, green line; this is the mean through time of the square of Fig 2a) only moderately exceeded the mean squared value of synchrony predicted by our best model on long timescales (Fig 2c, blue line; this is the mean through time of the square of Fig 2b for long timescales), but greatly exceeded it on short timescales (Fig 2c, red line). The fraction of long-timescale mean squared synchrony in PCI explained by our best long-timescale model was 61.1%. The fraction for short-timescales was only 3.1% (Methods). Thus growing season temperature and *C. finmarchicus* abundance were probably the dominant Moran effects producing synchrony (high WMFM) in PCI on long timescales (with temperature likely acting indirectly, see Discussion); and we have not explained short-timescale synchrony.

The significant fit on short timescales of the model with predictors *C. finmarchicus*, echinoderm, and decapod larvae abundances does not conflict with the result that the same model explains very little of the synchrony in PCI abundance; the two results illustrate that explaining dynamics and explaining synchrony are related but distinct concepts. The significant fit reflects the fact that local associations between the drivers and PCI are greater, on short timescales, than would be expected by chance. But spatial synchrony can only result when there is a strong association between drivers and PCI and, crucially, when the drivers themselves are substantially synchronous. The small fraction of synchrony accounted for at high frequencies indicates that most of the spatial synchrony at high frequencies results from factors other than the drivers we were able to identify. See the material on our wavelet Moran theorem in Methods for additional details.

### Synchrony is more than its top-down and climatic components

Having just shown that top-down and climatic Moran effects produced most of the synchrony (mean squared WMFM) in PCI on long timescales, we now explore whether these effects interacted.

Randomizations showed that interactions between the Moran effects of growing season temperature and *C. finmarchicus* were significant and substantial in their influence on long-timescale PCI synchrony. In one randomization test, mean squared model synchrony ($\left\{\right|r_{\sigma }^{\left(h\right)}\left(t\right){\left|\right\}}_{t}$ in Methods) for our best long-timescale model was compared to the same quantity computed after *C. finmarchicus* data were replaced by surrogate datasets randomized so their patterns of synchrony were preserved but relationships with growing season temperature were eliminated (called *spatially synchronous surrogates*, Methods). Mean squared model synchrony is distinct from mean squared model-predicted synchrony, which was pictured in Fig 2c, blue line (Methods). The latter depends on model synchrony as well as on the strength of relationship between the model and PCI.

**Fig 3:**
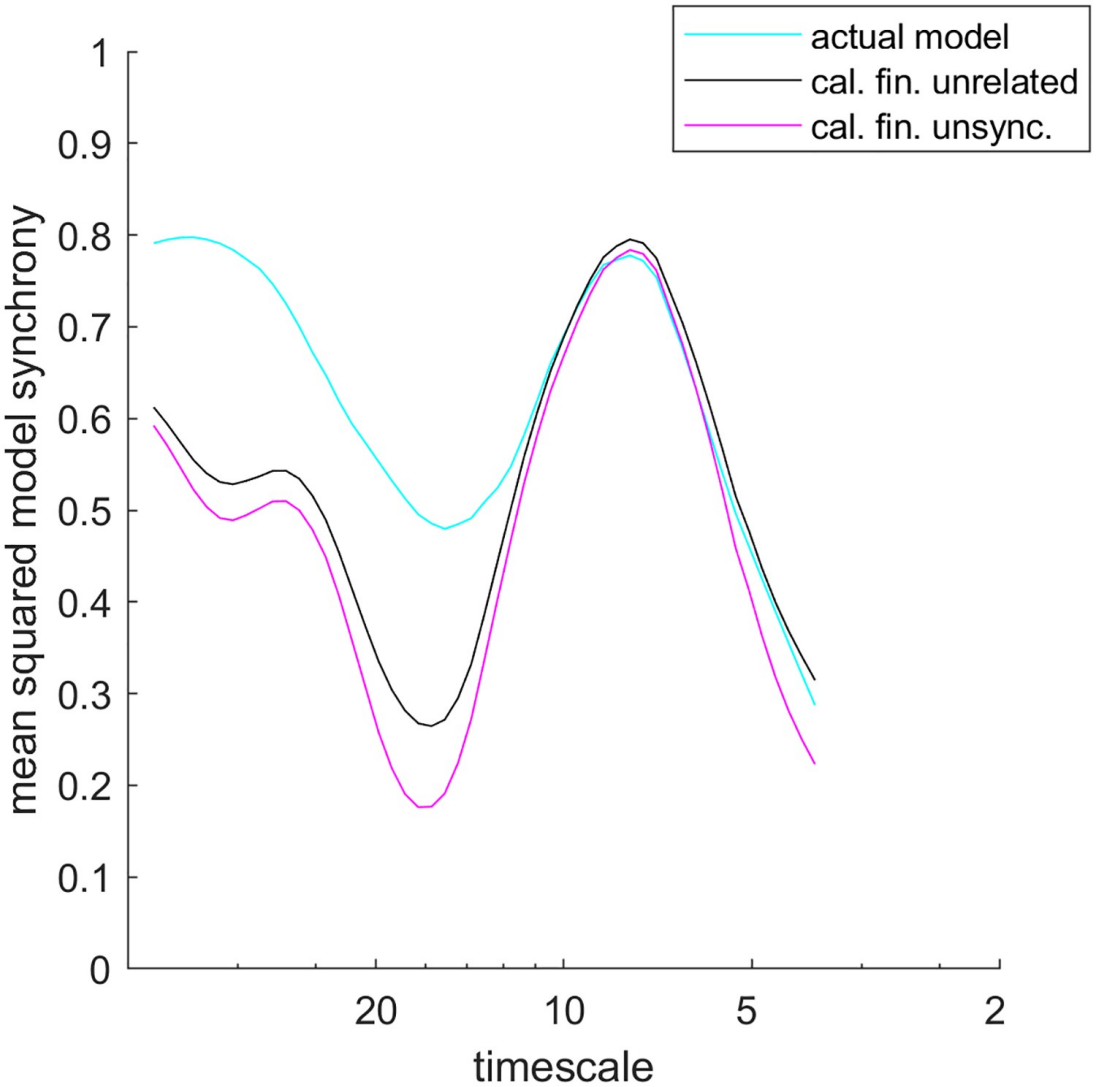
Randomizations revealed that interactions between Moran effects were important for long-timescale PCI synchrony. Mean squared model synchrony $\left\{\right|r_{\sigma }^{\left(h\right)}\left(t\right){\left|\right\}}_{t}$ (Methods) at long timescales for our best long-timescale model (cyan line), compared to $\left\{\right|r_{\sigma }^{\left(h\right)}\left(t\right){\left|\right\}}_{t}$ using synchrony-preserving surrogates (black line) and asynchronous surrogates (magenta line) of *C. finmarchicus* data. The best model had predictors growing season temperature and *C. finmarchicus* abundance, and synchrony-preserving surrogates randomized away interactions between these climatic and top-down Moran effects while retaining the effects themselves. Black and magenta lines show average results across 1000 surrogates. *C. fin*. = *C. finmarchicus*; unsync. refers to surrogates of the *C. finmarchicus* data for which synchrony, as well as relationships with temperature data, has been randomized away.

The reduction of synchrony from the randomization, attributable to the elimination of interactions between *C. finmarchicus* and temperature Moran effects, was substantial (Fig 3, cyan line versus black line). Thus these interactions contributed substantially to the synchrony of PCI. When *C. finmarchicus* data were instead replaced by asynchronous surrogates (Fig 3, magenta line), eliminating both interactions with temperature and *C. finmarchicus* synchrony itself, the further decrease was modest compared to the decrease attributable to interactions. This highlights the importance of interaction effects. The average of $\left\{\right|r_{\sigma }^{\left(h\right)}\left(t\right){\left|\right\}}_{t}$ across long timescales using spatially synchronous surrogates of *C. finmarchicus* was only 84% of the average using the actual, unmanipulated data. The actual data yielded higher average $\left\{\right|r_{\sigma }^{\left(h\right)}\left(t\right){\left|\right\}}_{t}$ than did 97.7% of spatially synchronous surrogates. Thus PCI synchrony is significantly higher than it would be if its two main Moran drivers were independent. Furthermore, we applied our synchrony attribution theorem (Methods) to calculate the fractions of long-timescale synchrony (mean squared WMFM) in PCI explained by, respectively, growing season temperature, *C. finmarchicus* abundance, and interactions between these: 41.4%, 5.4%, and 14.3%. Interaction effects were more important than the direct effects of *C. finmarchicus* abundance for PCI synchrony.

## Discussion

Through a combination of novel wavelet modeling and basic knowledge of plankton biology, we have provided compelling evidence that most of the long-timescale spatial synchrony (mean squared WMFM) in phytoplankton density in UK seas is due to climatic Moran effects related to temperature (41.4%), ‘top-down’ Moran effects of *C. finmarchicus*, a copepod consumer (5.4%), and, importantly, interactions between these effects (14.3%). It has been known for many years that *C. finmarchicus* and oceanographic phenomena related to temperature have important effects on the local dynamics of PCI, but our results go beyond earlier work by showing that these drivers and their interaction produce timescale-dependent synchrony through the Moran effect. To our knowledge this is the first study to demonstrate interacting Moran effects. It is reasonable to expect that similar interacting Moran effects may be a major cause of synchrony in other systems because of the complex of related factors driving most population fluctuations. Our results also confirm and extend the conclusions of a growing body of work [7, 9, 15, 20, 29, 37, 38] that synchrony is strongly timescale-dependent.

Other major outcomes of our study are methodological. We described and applied wavelet regression methods that appropriately account for spatial and temporal autocorrelation of variables and that make possible model selection to determine possible drivers of synchrony. The wavelet modeling approach introduced here is intended to examine the specific problems of timescale specificity in the action and effectiveness of driving variables on spatial synchrony; and phase delays that may be part of ecological influences. Traditional linear models do not partition variation by timescale. ANOVA- or regression-based approaches will only identify time-delayed effects if the delay is specified before fitting. Autoregressive models can include time-delayed effects in the form of additional terms but many such terms must be estimated to model complex cross-spectral relationships across a range of timescales, and interpretations of results become correspondingly difficult. By examining all possible timescales, as our wavelet techniques do, we were able to represent any phase delay and proportionality relationship that is found at a given timescale by a single complex number, simplifying both the statistical testing and interpretation of such relationships. In particular, the quarter-cycle phase shift between temperature and its effect on plankton on long timescales is naturally represented in this way. Our new wavelet Moran theorem and synchrony attribution theorem are useful to partition the amount of synchrony due to individual factors and to interactions. Our approaches are based on and extend a long history of using wavelets as a standard tool for time series analysis [32], many years of application in ecology (e.g., [39, 40]), and a growing literature in which wavelets and other spectral methods are applied to synchrony [7, 9, 15, 20, 28–30].

Our models are statistical and do not, taken completely on their own, reveal causal relationships in plankton dynamics or Moran effects. Nevertheless, our statistical results combined with biological reasoning to make compelling the hypothesis that long-timescale fluctuations in PCI have both climatic and top-down causes, and that PCI synchrony is therefore produced in large part through joint Moran effects. Strong spatial coherence between two variables, A and B (as we observed on long timescales for A = PCI and B = growing season temperature or *C. finmarchicus*), means that either: A or a closely related variable causally influences the dynamics of B; or *vice versa*; or causation is bi-directional; or that some third variable influences A and B. If A is temperature and B is PCI, the only reasonable possibility is that temperature and/or some related oceanographic variable influences PCI, possibly via complex mechanisms (see below). Thus there was a climatic Moran effect. Spatial coherence between *C. finmarchicus* and PCI may be expected because phytoplankton is a food resource for *C. finmarchicus*. The approximate anti-phase relationship between *C. finmarchicus* and PCI supports the hypothesis that increased *C. finmarchicus* abundance reduces PCI abundance through grazing, bringing about a Moran effect on PCI. If, instead, the reverse causal hypothesis held, i.e., high prey abundance supported high predator abundance, then the two variables would be in phase. Bi-directional causation in a classic predator-prey cycle would suggest a phase difference of *π*/2, not observed. Thus there was a top-down Moran effect. *C. finmarchicus* is known to feed on microheterotrophs which do not contribute to PCI. In fact, microheterotrophs are a sufficient resource for *C. finmarchicus* to perform lipid synthesis and egg production, and egg production depends on a number of factors including food supply, phenology, and temperature, of which green phytoplankton abundance is only one [41–44]. Thus green phytoplankton abundance is apparently not a limiting factor, enabling *C. finmarchicus* to causally influence PCI in an anti-phase relationship, without the strong feedbacks associated with specialist predator-prey relationships.

Our synthetic example (Fig 1) illustrates and illuminates the mechanisms our results support as underlying PCI synchrony. Model synchrony is high in part because fluctuations in PCI attributable to the two variables (growing season temperature and *C. finmarchicus* abundance) are reinforcing—they are in phase. The effects of temperature (probably indirect—see below) are shifted by a quarter-cycle relative to temperature itself, and are reinforced by the effects of *C. finmarchicus*, which are in anti-phase with *C. finmarchicus* itself. These effects match the effects illustrated in the example (Fig 1) of *β* and *α*, respectively, on *γ*^(3)^. If phase relationships among the variables were to shift, interaction effects could cease or become negative, as in the synthetic example, reducing PCI synchrony. This could occur even if the strength of PCI dependence on these drivers and the spatial synchrony of the drivers remained unchanged, just as analogous phase shifts reduced synchrony in the synthetic example (Fig 1i, green line).

Long timescale drivers of synchrony, such as temperature, are less likely to be detectable in short time series data than are short-timescale covariates such as echinoderm larvae abundance. The existence of a phase shift between drivers and PCI makes detections with limited data even more difficult via correlation-based approaches. Nevertheless, these long-timescale relationships should not be overlooked as they are strong drivers of synchrony. They are also very relevant to consideration of a persistent change in the ecosystem such as a regime shift.

The long-timescale climatic Moran effects that our analysis reveals are unlikely to be direct effects solely of temperature on phytoplankton growth and abundance; they are more likely to reflect Moran effects of multifaceted oceanographic processes implicating water-column mixing, nutrients, and light penetration as well as temperature. In many marine systems, sea surface temperature is closely negatively correlated with near-surface nutrient concentrations because vertical mixing elevates sub-surface waters which are both cold and nutrient rich. Also, surface heating tends to stratify the water column, which leads to the depletion of nutrients in the mixed layer. Mixing also affects light penetration by increasing concentrations of suspended sediments. These interrelated processes are often studied on seasonal timescales [12], whereas our statistical results pertain to timescales > 4 years. Our results only reveal the importance of climatic Moran effects for PCI synchrony, not the detailed oceanographic mechanisms. Detailed study of mechanisms may be an important topic of future work. Oceanographic mechanisms of synchrony have been explored, for example, for the sub-polar and sub-tropical North Atlantic [14], away from the continental shelf.

Defriez *et al*. demonstrated that dual Moran effects of temperature and precipitation help produce synchrony of terrestrial vegetation on global scales, though they did not find interaction effects between these factors [9]. Their statistical approach was geographic, and differed fundamentally from ours. But the two approaches are complementary, and it may be possible to combine them to maximize the potential for the statistical detection of causes of synchrony and interaction effects. Walter *et al*. provide some initial steps toward integrating spatial and wavelet approaches [37].

At certain timescales there is a lot of reinforcement between drivers of PCI synchrony (Fig 3, 18-year timescale, compare cyan and black lines), yielding additional synchrony; at other timescales less (Fig 3, 8-year timescale). These differences between timescales in the degree of reinforcement between Moran effects may possibly be a consequence of particular processes of climatic variability, with their own characteristic timescales, being incorporated into both drivers, thereby creating reinforcement only on those characteristic timescales. The underlying timescale-specific nature of interactions between Moran drivers could be another fruitful topic of future research.

Powerful parametric methods now exist (e.g., [45]) to fit complex nonlinear mechanistic models to spatiotemporal population data sets to infer system parameters and mechanisms driving dynamics. Although these methods have been used effectively, especially for infectious disease dynamics, we pursued nonparametric wavelet approaches instead because we think the space of possible dynamical models for plankton in UK seas is still too poorly constrained for the mechanistic approach. The approach proceeds by proposing a suite of alternative models which may represent the dynamics of a system. Models are fitted to data using powerful modern statistical tools, and model selection is used to determine which model, and therefore which combination of mechanisms, is best supported. For instance, cholera dynamics in Bengal, for which high resolution data were available, were confronted with a few alternative models to make valuable inferences about the importance of asymptomatic or unreported cases [46]. But plankton include hundreds of species, and thousands of reasonable alternative models could probably be specified. Each model would be elaborate, and available data may not be sufficient to adequately constrain parameters. Our nonparametric wavelet approach can provide mechanistic insights for complex systems, helping reduce the space of possible models and possibly paving the way for future mechanistic modeling.

Recent evidence suggests that changes in the synchrony of climatic drivers and the populations they influence may be a more important consequence of global change than previously recognized [7, 20, 47–50]. *C. finmarchicus* is in decline in UK seas because it is a cold-water species and is shifting northward with climatic warming [26]; it is being replaced by *C. helgolandicus*. Relative to *C. finmarchicus*, *C. helgolandicus* had different phase relationships with both PCI and growing season temperature (details not shown), so our results show the potential for major changes in PCI synchrony stemming from both changes in direct top-down Moran effects and changes in interactions between top-down and climatic Moran effects (Appendix S2 and S3 Fig for details and substantial uncertainties about the future state of the North Sea ecosystem). Phytoplankton are at the root of the marine food web, and it is known that patterns of phytoplankton synchrony and patchiness have a major influence on the foraging of higher-trophic-level organisms [20, 51, 52]. Thus understanding the future of phytoplankton synchrony has major practical implications. We hope our methods and the understanding we provided of the causes of past PCI synchrony serve as a launch point for important future research predicting how patterns of plankton synchrony in the oceans may be altered.

## Materials and methods

### Data

The CPR survey has consistently monitored plankton in the seas around the British Isles, on a monthly basis since January 1946 [53]. The survey, operated by SAHFOS from 1999 until recently when it was taken over by the MBA, uses voluntary merchant ships of opportunity to tow the CPR sampling device. At any given time several devices are in use, towed along different shipping routes. The device traps plankton on a continuously moving ribbon of silk, which is later cut into sections for microscope analysis. Each section represents a 10 nautical mile transect made at a particular time and place. The number of organisms of various types is recorded, together with the PCI, which corresponds to the amount of chlorophyll retained on the ribbon. Detailed descriptions of the survey are published elsewhere [22, 54].

The PCI has four values corresponding to observations of ‘No Green’, ‘Very Pale Green’, ‘Pale Green’, and ‘Green’. Colebrook gives the dilution factors needed to render samples with different colors the same color: ‘Very Pale Green’ = 1, ‘Pale Green’ = 2, ‘Green’ = 6.5 [53]. We used Colebrook’s original dilution factors to generate our time series (Appendix S3). Other authors indicate that the PCI is well correlated with satellite ocean chlorophyll measurements during the satellite period [21, 22, 55].

The ecological and physical variables to be compared with PCI and examined as potential Moran drivers or covariates are shown in Tables 1 and 2, respectively. We examined densities of 13 major zooplankton components from the CPR, including the important phytoplankton consumers *C. finmarchicus* and *C. helgolandicus*, calanoid copepod species. Data about physical oceanographic variables were downloaded from the International Comprehensive Ocean-Atmosphere Data Set (ICOADS) Release 2.5. We investigate measurements of temperature and salinity made in the top ten meters of the water column, and measurements of wind speed and cloud cover made at the sea surface.

Measurements of each of the biological and physical variables were compiled into 26 time series representing 2 by 2 degree areas of sea (Appendix S3, S4 Fig). Our annualized data reflect changes in the growing conditions of the annual phytoplankton bloom. Many generations of organisms contribute to the phytoplankton bloom each year, and their overall abundance is driven by climatic and other factors that vary on long timescales, and in the case of some climatic phenomena, periodically. This results in corresponding inter-annual variation in the PCI, which can be identified with timescale-specific methods. Annual time series for several variables are in S5–S9 Figs. For each variable the annualized time series were subjected to Box-Cox normalization (see Appendix S4, S2 Table, and [56]).

### Statistical methods

We use a variety of methods, some new. Here we summarize the techniques and how to interpret their outputs, with details in the Supporting Information. Please see www.github.com/reumandc/wsyn for a software package for the R statistical programming language containing code for the techniques of this study and a vignette explaining how to use the code. We start with *wavelet transforms*, on which other methods are based. Time series can be regarded as composed of fluctuations on different timescales (i.e., of different characteristic oscillatory periods). The wavelet transform provides time- and timescale-specific information on these fluctuations. We use a complex Morlet transform, as demonstrated in S10 Fig and described mathematically in Appendix S5.

The *spatial coherence* [7] of two spatiotemporal variables $x_{n}^{\left(0\right)}\left(t\right)$ and $x_{n}^{\left(1\right)}\left(t\right)$ measured at locations *n* = 1, …, *N* and times *t* = 1, …, *T* quantifies, in a timescale-specific way, the strength of the association between the variables. The spatial coherence, which is based on the ‘power-normalized’ (Appendix S5) wavelet transforms $w_{n,\sigma }^{\left(0\right)}\left(t\right)$ and $w_{n,\sigma }^{\left(1\right)}\left(t\right)$ of $x_{n}^{\left(0\right)}\left(t\right)$ and $x_{n}^{\left(1\right)}\left(t\right)$, is a function of timescale, *σ*. It takes values between 0 and 1, with higher values meaning a stronger association (Appendix S6).

Unlike commonly used correlation measures, the spatial coherence has the benefit of finding associations even if fluctuations are strongly related only on particular timescales, and even if there are phase delays between the corresponding fluctuations. A demonstration of the spatial coherence technique is in S11 Fig.

We approached question 1 from the Introduction via a multivariate linear modeling approach for wavelet transforms (Appendix S7) that made it possible to quantify determinants of synchrony in PCI and their interactions. If $x_{n}^{\left(k\right)}\left(t\right)$ for *k* = 0, …, *K* are spatiotemporal variables (*n* = 1, …, *N*, *t* = 1, …, *T*) and $w_{n,\sigma }^{\left(k\right)}\left(t\right)$ are their power-normalized wavelet transforms (Appendix S5), then the linear models statistically explain variation in the response transforms $w_{n,\sigma }^{\left(0\right)}\left(t\right)$ in terms of the predictor transforms $w_{n,\sigma }^{\left(k\right)}\left(t\right)$:

$$\begin{array}{c} w_{n,\sigma }^{\left(0\right)}\left(t\right)\approx \beta_{1}\left(\sigma \right)w_{n,\sigma }^{\left(1\right)}\left(t\right)+\cdots +\beta_{K}\left(\sigma \right)w_{n,\sigma }^{\left(K\right)}\left(t\right). \end{array}$$ (2)

We take $x_{n}^{\left(0\right)}\left(t\right)$ to be PCI, and $x_{n}^{\left(k\right)}\left(t\right)$ for *k* > 0 to be potential influences on PCI (different variables selected from Tables 1 and 2 in different models). Such models can be fitted with data; for a given model, the fitted coefficients of the model, which are functions of timescale, maximize (Appendix S7) the spatial coherence between the left and right sides of Eq 2 at every timescale. Two nested models can be compared statistically using surrogate resampling approaches (Appendix S8) which are standard in wavelet statistics; conceptually, these are analogous to standard *F*-tests between nested linear models in the sense that a more complex model is tested against a simpler alternative. But *F*-distributions are not used in the wavelet case. Conventional significance tests assuming serially independent data are inappropriate for spatially synchronous wavelet transform data in which correlations are present between the values. The Fourier surrogates we use (Appendix S8) are a standard alternative.

A model selection and testing procedure (Appendix S9) determines which predictors *k* = 1, …, *K* should optimally be included for a given range of timescales. These predictors are the good candidates for statistically explaining PCI dynamics and synchrony on those timescales. For a model to be ranked, each predictor transform $w_{n,\sigma }^{\left(k\right)}\left(t\right)$ (*k* > 0) included in the model was required to provide a significant improvement over the model with that predictor removed. Among those models for which all predictors were significant, we performed leave-one-out cross validation to select the best model and avoid over fitting. Models were constructed separately for short (< 4 years) and long (> 4 years) timescales. The choice of these two timescale ranges was justified in the main text and in Appendix S9.

Best models selected by the model selection procedure were tested in additional ways (Appendix S10-Appendix S12) beyond the testing that was built into the selection procedure (Appendix S9). First, the phases of the model coefficients *β*_*k*_(*σ*) are supposed to represent the phase lag of effects (or associations) at timescale *σ* of $x_{n}^{\left(k\right)}\left(t\right)$ on $x_{n}^{\left(0\right)}\left(t\right)$ (Eq (2) for notation). Strong associations which are consistent over time and space should therefore produce a correspondence between the phase of *β*_*k*_(*σ*) and the average phase, over space and/or time, of $w_{n,\sigma }^{\left(0\right)}\left(t\right)\bar{w_{n,\sigma }^{\left(k\right)}\left(t\right)}$, where the overbar denotes complex conjugation; the phase of this quantity is the phase difference between $x_{n}^{\left(0\right)}\left(t\right)$ and $x_{n}^{\left(k\right)}\left(t\right)$ at timescale *σ*, time *t*, and location *n*. We tested for this phase correspondence and found it to be good (Appendix S10). The phases of the *β*_*k*_ for our best-fitting models were extracted for interpretation of model output (Results), to determine the lag between the fluctuations in the *k*th predictor and the corresponding content in the PCI wavelet transforms. Details of this process are also in Appendix S10.

Another test of the potential usefulness of our top-ranked models as tools to reveal Moran effects had to do with whether the models represented ‘local associations’. An alternative possibility is that PCI is statistically associated with these variables on average across the the British seas sampled, but that seawater mixing and dispersal prevent the dynamics of PCI from being attributable to local drivers. This alternative possibility would suggest a dominant role of dispersal as a synchronizing agent rather than Moran effects. We tested for local associations between PCI and the four predictor variables appearing in top models at long or short timescales through a spatial permutation test. Results indicated that associations were local in all cases, with details in Appendix S11.

Finally, spatial coherences between nine individual phytoplankton species abundances from the CPR data set and *C. finmarchicus* were tested in earlier work [38], and those results were also consistent with our findings that PCI and *C. finmarchicus* were significantly spatially coherent and in anti-phase (Appendix S12).

Given a spatiotemporal dataset *x*_*n*_(*t*), we used two tools for describing the synchrony among these time series, and its time and timescale dependence. The *wavelet mean field magnitude* (WMFM) plot, here denoted |*r*_*σ*_(*t*)|, is the magnitude of $r_{\sigma }\left(t\right)=\frac{1}{N}\sum_{n=1}^{N}w_{n,\sigma }\left(t\right)$. The WMFM plot is applied in Fig 1b, 1c and 1e–1g, from which one can get a sense of the nature of the output of the method and how to interpret that output. See Appendix S13 for details. We refer to the quantity $\frac{1}{T}\sum_{t}|r_{\sigma }(t){|}^{2}={\left\{|r_{\sigma }(t)|\right\}}_{t}$ as the *mean squared synchrony* of the *x*_*n*_(*t*), where {⋅}_*t*_ here and henceforth denotes the time average of the square of the quantity in braces. The *wavelet phasor mean field magnitude* (WPMFM; Appendix S13) provides a plot in the same format as the WMFM, but quantifies only the phase synchrony of the *x*_*n*_(*t*) as a function of time and timescale. Phase synchrony is always between 0 and 1 and equals 1 at the *t* and *σ* for which time series have identical phases of oscillation. Phase synchrony can be straightforwardly tested for significance at *t* and *σ*. Details on these techniques are in Appendix S13 and a demonstration is in S12 Fig. WMFM and WPMFM plots for PCI are in S2 Fig, and for several other variables in S13–S16 Figs.

Having determined robust wavelet models (Eq 2) of PCI transforms for long and short timescales as described above, we applied a new ‘wavelet Moran theorem’ (Appendix S14) to quantify to what extent the synchrony in PCI can be explained by the predictors included in our best models. The theorem is a generalization of an earlier result [7]. A wavelet model can be a good model, in the sense that it is validated by our testing procedures and explains a significant amount of variation in $w_{n,\sigma }^{\left(0\right)}\left(t\right)$, without contributing to synchrony. Our Moran theorem allows us to quantify how well the predictors explain synchrony. In words, the theorem states that at timescale *σ*, if the only synchronizing influences on a biological variable $x_{n}^{\left(0\right)}\left(t\right)$ are the biotic or abiotic variables $x_{n}^{\left(k\right)}\left(t\right)$ for *k* = 1, …, *K*, then the strength of synchrony of the biological variable is the strength of synchrony in the best-fitting wavelet model times a spatial coherence of that model with the biological variable. In formulas, $\left\{\right|r_{\sigma }^{\left(0\right)}{\left(t\right)|\}}_{t}\approx \left\{\right|r_{\sigma }^{\left(h\right)}{\left(t\right)|\}}_{t}{\left|\Pi_{\sigma }^{\left(0h\right)}\right|}^{2}$, where $|r_{\sigma }^{\left(0\right)}\left(t\right)|$ is the WMFM of $x_{n}^{\left(0\right)}\left(t\right)$, $|r_{\sigma }^{\left(h\right)}\left(t\right)|$ is a WMFM for the best-fitting wavelet model, and $|\Pi_{\sigma }^{\left(0h\right)}|$ is a spatial coherence of the model with the biological variable. The left side measures synchrony of the biological variable and the first multiplicand measures model synchrony for a model that accounts for the variables $x_{n}^{\left(k\right)}\left(t\right)$, *k* = 1, …, *K*. The theorem formalizes the reasoning that synchrony of a driven variable should depend on synchrony of the combined effects of the drivers and on the strength of the influence of the drivers. Additional independent drivers may act to increase synchrony of the $x_{n}^{\left(0\right)}\left(t\right)$, turning the approximate equality, ≈, into an inequality, >. The right side of the Moran formula is then the amount of synchrony attributable to the drivers $x_{n}^{\left(k\right)}\left(t\right)$ for *k* = 1, …, *K*.

The theorem also provides a ‘predicted synchrony’ plot for a model, which has format like the WMFM plot $|r_{\sigma }^{\left(0\right)}\left(t\right)|$ and can be compared to it, but shows the pattern of synchrony that would pertain if the only synchronizing influences on $x_{n}^{\left(0\right)}\left(t\right)$ were the variables $x_{n}^{\left(k\right)}\left(t\right)$ (Appendix S15). Observed synchrony of $x_{n}^{\left(0\right)}\left(t\right)$ (i.e., $|r_{\sigma }^{\left(0\right)}\left(t\right)|$) is expected to be strictly greater than predicted synchrony, the difference corresponding to synchronizing influences not included in the model. Using the Moran theorem, it is straightforward to quantify the fraction of synchrony explained by the model for a timescale band (Appendix S15). With $x_{n}^{\left(0\right)}\left(t\right)$ being PCI, we calculated predicted synchrony and fractions of synchrony explained for long and short timescales using our best long- and short-timescale models, respectively.

To investigate whether interaction effects of Moran drivers were important for the synchrony of PCI, we considered mean squared model synchrony $\left\{\right|r_{\sigma }^{\left(h\right)}\left(t\right){\left|\right\}}_{t}$ of the best models chosen by our methods, and we analyzed how it was affected by randomizations of predictor variables that eliminated any interaction effects that may have occurred (Appendix S16).

We also proved an extension of our wavelet Moran theorem (theorem 1 Appendix S14), dubbed the ‘synchrony attribution theorem’ (theorem 2 Appendix S17), that makes it possible to partition synchrony explained by a wavelet model into components explained by individual predictors, and a component explained by interaction effects between predictors. We applied the theorem with $x_{n}^{\left(0\right)}\left(t\right)$ again being PCI. We tested our methods on the numerical example of the Theoretical illustration section of Results (Appendix S18).

## Supporting information

S1 Text One supplementary text document is provided, including Appendix S1 to S19. (PDF)

S1 Table Table of best long-timescale models. Of all models we considered (Methods), the model with the highest leave-one-out goodness of fit score for which no variables could be dropped without significantly reducing model fit was the model listed in the top row and analyzed in the main text. The table includes all models considered for which the leave-one-out goodness of fit score was at least 90% that of the top model and for which no variables could be dropped without significantly reducing model fit.
(PDF)

S2 Table The mean of the Box-Cox coefficients found at each location, for every variable. (PDF)

S1 FigPhytoplankton wavelet phase shifts relative to four explanatory variables, compared to phases of coefficients of top-ranked models, for the purposes of testing and interpreting top-ranked models.Top panels: The typical phase difference, *φ*, i.e. the phase of $\frac{1}{N}\sum_{n}e^{i\eta (n,t)}$, where *η*(*n*, *t*) is the phase of $w_{n,\sigma }^{\left(0\right)}\left(t\right)\bar{w_{n,\sigma }^{\left(k\right)}\left(t\right)}$, for the four predictors *k* appearing in the top-ranked models at long and short timescales: growing season temperature (a); *C. finmarchicus* abundance (b); echinoderm larvae abundance (c); and decapod larvae abundance (d). Bottom panels: The typical phase difference, *θ*, i.e. the phase of $\frac{1}{NT}\sum_{n,t}e^{i\eta (n,t)}$ (green), compared to phases of corresponding coefficients *β*_*k*_(*σ*) from top-ranked models (magenta). Magenta lines extend across long timescales for predictors included in the top-ranked long-timescale model, across short timescales for predictors included in the top-ranked short-timescale model, and across long and short timescales for *C. finmarchicus*, since that variable was in the top-ranked models for both long and short timescales. Temp. = growing season temperature; *C. fin*. = *C. finmarchicus*; Ech. = echinoderm; Dec. = decapod.(JPG)

S2 Fig The synchrony of PCI expressed as wavelet mean field magnitude (a) and wavelet phasor mean field magnitude (b) plots. Statistical significance thresholds on the WPMFM are plotted as contours showing actual phase agreement between locations greater than the 90th, 95th, 99th and 99.9th percentile of a distribution of unsynchronized unit phasors (dotted, dash-dotted, dashed and line contours respectively).
(JPG)

S3 Fig Like Fig 3 from the main text, but with one additional line (the red line) representing mean squared model synchrony after setting the coefficient *β*_*k*_(*σ*) of the *C. finmarchicus* term in the best long-timescale model (see main text) to 0. (JPG)

S4 Fig The 26 areas of the North Sea and British seas which were used. (JPG)

S5 Fig The annualized time series for PCI (mean of 12 monthly values). Plots numbered as in S4 Fig.
(JPG)

S6 Fig The annualized time series for growing season temperature (mean of monthly values for March to September). Plots numbered as in S4 Fig.
(JPG)

S7 Fig The annualized time series for *C. finmarchicus* abundance (mean of 12 monthly values). Plots numbered as in S4 Fig.
(JPG)

S8 Fig The annualized time series for echinoderm larvae abundance (mean of 12 monthly values). Plots numbered as in S4 Fig.
(JPG)

S9 Fig The annualized time series for decapod larvae abundance (mean of monthly values). Plots numbered as in S4 Fig.
(JPG)

S10 Fig Demonstrations of the detection of oscillations on different timescales for different times using the magnitude of the wavelet transform. The time series of panel d was the sum of: 1) a sine wave of amplitude 1 and period 15 that operated for the first half of the time series (a); 2) a sine wave of amplitude 1 and period 8 that operated for the second half of the time series (b); and 3) normally distributed white noise of standard deviation 0.5 (c). Although periodicities in (d) and changes therein are difficult to detect by eye with any certainty, the magnitude of the wavelet transform (f; Appendix S5 in S1 Text) reveals them clearly. The time series of (e) changes period gradually from 5 to 10. The magnitude of the wavelet transform (g) shows the change.
(PNG)

S11 Fig Measuring relationships between variables using spatial coherence. This figure was adapted with only minor changes from supplementary figure 5 of [7]. The time series of panel e were used as drivers in producing the time series of panel f and the figure shows how this relationship can be detected with the spatial coherence technique. The time series of panel e were constructed as the sum of: 1) a single common signal of amplitude 1 and period 12 years (a); 2) a single common signal of amplitude 5 and period 2 years (b); and 3) normally distributed white noise of standard deviation 1.5, independently generated for each of the 10 time series (c). The time series of panel f were produced via the relationship *y*_*n*_(*t*) = (*x*_*n*_(*t*) + *x*_*n*_(*t* − 1))/2 + *ϵ*_*n*_(*t*) where the *ϵ*_*n*_(*t*) were independent normal random numbers of mean 0 and standard deviation 3. This transmits the period-12 component of the *x* signals to the *y* but not the period-2 component because averaging covers a whole period for that component. Correlations (f, green numbers) between *x*_*n*_(*t*) and *y*_*n*_(*t*) did not indicate any particular relationship. Correlations cannot detect the relationship between the *x* and *y* because the technique confounds phenomena occurring on different timescales. Spatial coherences revealed a highly significant relationship at periods around 12 years (g) and on average over long timescales (left *p*-value on panel g, long timescales defined as > 4 years) but no relationship (right *p*-value) for short timescales (< 4 years). The red line on g is the spatial coherence and black lines are 50th, 95th, and 99th percentiles of spatial coherences of synchrony-preserving surrogate data sets (Appendix S8 in S1 Text) appropriately representing the null hypothesis of no relationship between the *x* and *y*. See also Appendix S9 in S1 Text for a description of how the aggregate long- and short-timescale *p*-values were computed.
(PNG)

S12 Fig Detecting time- and timescale-specific synchrony with the wavelet mean field. This figure was taken without change from [7]. Panels a-d show the principle of how synchrony can differ for different timescales of dynamics. Time series y1 and y2 (a) are exactly anti-correlated (out of phase), as are y3 and y4 (b). Combining y1 with y3 and y1 with y4 gives two time series (c) which are synchronized on long timescales, but anti-synchronized on short timescales. The reverse is also possible (d). This timescale-specific structure of synchrony cannot be detected with correlation coefficients, which are 0 for both c and d, because contributions from different timescales cancel. In practice, real population and environmental signals are broadband, and exact cancelation is unlikely, but asynchrony at some frequencies can strongly conceal important synchrony at other frequencies. Panels e-k demonstrate this concealment, using artificial data, and also show how the wavelet mean field detects time- and timescale-specific synchrony. Each of 11 artificial time series were constructed as the sum of: 1) a single common signal of amplitude 1 that changes its oscillatory period at *t* = 50 from 10 years to 5 years (e); 2) oscillations of amplitude 3 that have the same oscillatory period (3 years), but random and independent phases in each of the 11 constructed time series (f); and 3) white noise of standard deviation 1.5, again independently generated for each of the 11 time series (g). Synchrony in the resulting time series cannot be visually detected (i), nor is it readily apparent by examining the 55 pairwise correlation coefficients between time series (j), which spanned a wide range of values including 0. But the wavelet mean field magnitude (k) showed clear color bands at 10-year period for *t* < 50 and 5-year period for *t* > 50. The wavelet mean field magnitude displays strength of synchrony as a function of timescale of dynamics and time, here with red indicating synchrony and blue asynchrony. Wavelet phasor mean fields provide plots similar to (k) but with values between 0 and 1 that indicate the strength of phase synchrony and that can be straightforwardly significance tested as describer in Appendix S13 in S1 Text.
(PNG)

S13 Fig The synchrony of temperature expressed as wavelet mean field magnitude (a) and wavelet phasor mean field magnitude (b) plots. Statistical significance thresholds on the WPMFM are plotted as contours showing actual phase agreement between locations greater than the 90th, 95th, 99th and 99.9th percentile of a distribution of unsynchronized unit phasors (dotted, dash-dotted, dashed and line contours respectively).
(JPG)

S14 Fig The synchrony of *C. finmarchicus* abundance fluctuations expressed as wavelet mean field magnitude (a) and wavelet phasor mean field magnitude (b) plots. Statistical significance thresholds on the WPMFM are plotted as contours showing actual phase agreement between locations greater than the 90th, 95th, 99th and 99.9th percentile of a distribution of unsynchronized unit phasors (dotted, dash-dotted, dashed and line contours respectively).
(JPG)

S15 Fig The synchrony of echinoderm larvae abundance fluctuations expressed as wavelet mean field magnitude (a) and wavelet phasor mean field magnitude (b) plots. Statistical significance thresholds on the WPMFM are plotted as contours showing actual phase agreement between locations greater than the 90th, 95th, 99th and 99.9th percentile of a distribution of unsynchronized unit phasors (dotted, dash-dotted, dashed and line contours respectively).
(JPG)

S16 Fig The synchrony of decapod larvae abundance fluctuations expressed as wavelet mean field magnitude (a) and wavelet phasor mean field magnitude (b) plots. Statistical significance thresholds on the WPMFM are plotted as contours showing actual phase agreement between locations greater than the 90th, 95th, 99th and 99.9th percentile of a distribution of unsynchronized unit phasors (dotted, dash-dotted, dashed and line contours respectively).
(JPG)

## References

[1] Liebhold A, Koenig WD, Bjørnstad O. Spatial synchrony in population dynamics. Annual Review of Ecology Evolution and Systematics. 2004;35:467–490. 10.1146/annurev.ecolsys.34.011802.132516

[2] Moran PAP. The statistical analysis of the Canadian Lynx cycle. Australian Journal of Zoology. 1953;1:291–298. 10.1071/ZO9530163

[3] Cheal AJ, Delean S, Sweatman H, Thompson AA. Spatial synchrony in coral reef fish populations and the influence of climate. Ecology. 2007;88(1):158–169. 10.1890/0012-9658(2007)88[158:SSICRF]2.0.CO;2 17489464

[4] Grenfell BT, Wilson K, Finkelstäd BF, Coulson TN, Murray S, Albon SD, et al Noise and determinism in synchronized sheep dynamics. Nature. 1998;394:674–677. 10.1038/29291

[5] Post E, Forchhammer MC. Synchronization of animal population dynamics by large-scale climate. Nature. 2002;420:168–171. 10.1038/nature01064 12432390

[6] Haynes KJ, Bjørnstad ON, Allstadt AJ, Liebhold AM. Geographical variation in the spatial synchrony of a forest-defoliating insect: isolation of environmental and spatial drivers. Proceedings of the Royal Society B-Biological Sciences. 2013;280:20122373 10.1098/rspb.2012.2373 PMC3574343 23282993

[7] Sheppard LW, Bell JR, Harrington R, Reuman DC. Changes in large-scale climate alter spatial synchrony of aphid pests. Nature Climate Change. 2016;6:610–613. 10.1038/nclimate2881

[8] Haydon D, Steen H. The effects of large– and small–scale random events on the synchrony of metapopulation dynamics: a theoretical analysis. Proceedings of the Royal Society B, Biological Sciences. 1997;264:1375–1381. 10.1098/rspb.1997.0191

[9] Defriez EJ, Reuman DC. A global geography of synchrony for terrestrial vegetation. Global Ecology and Biogeography. 2017;26(8):878–888. 10.1111/geb.12595

[10] Kendall BE, Bjørnstad ON, Bascompte J, Keitt TH, Fagan WF. Dispersal, Environmental Correlation, and Spatial Synchrony. The American Naturalist. 2000;155:628–636. 10.1086/303350 10777435

[11] Brennan G, Collins S. Growth responses of a green alga to multiple environmental drivers. Nature Climate Change. 2015;5:892–897. 10.1038/nclimate2682

[12] Williams RG, Follows MJ. Ocean Dynamics and the Carbon Cycle: Principles and Mechanisms. Cambridge, UK: Cambridge University Press; 2011.

[13] Dutkiewicz S, Follows M, Marshall J, Gregg WW. Interannual variability of phytoplankton abundances in the North Atlantic. Deep-Sea Research Part II, Topical studies in Oceanography. Deep-Sea Research II. 2001;48:2323–2324. 10.1016/S0967-0645(00)00178-8

[14] Follows M, Dutkiewicz S. Meteorological modulation of the North Atlantic spring bloom. Deep-Sea Research Part II, Topical studies in Oceanography. 2002;49:321–344. 10.1016/S0967-0645(01)00105-9

[15] Defriez EJ, Reuman DC. A global geography of synchrony for marine phytoplankton. Global Ecology and Biogeography. 2017;26(8):867–877. 10.1111/geb.12594

[16] Steele J. Spatial Patterns in Plankton Communities. New York, USA: Springer; 1978.

[17] Colebrook JM. Continuous Plankton Records: zooplankton and environment, north-east Atlantic and North Sea, 1948-1975. Oceanologica Acta. 1978;1:9–23.

[18] Colebrook JM. Environmental influences on long-term variability in marine plankton. Hydrobiologia. 1986;142(309–325). 10.1007/BF00026767

[19] Reid P, Beaugrand G. Large Marine Ecosystems of the North Atlantic. Sherman K and Skjoldal H R, editor. Elsevier Science B.V.: Elsevier Science Amsterdam; 2002.

[20] Defriez EJ, Sheppard LW, Reid PC, Reuman DC. Climate change-related regime shifts have altered spatial synchrony of plankton dynamics in the North Sea. Global Change Biology. 2016;22:2069–2080. 10.1111/gcb.13229 26810148

[21] Raitsos DE, Walne A, Lavendar SJ, Licandro P, Reid PC, Edwards M. A 60-year ocean colour data set from the continuous plankton recorder. Journal of Plankton Research. 2013;35:158–164. 10.1093/plankt/fbs079

[22] Raitsos DE, Pradhan Y, Lavender SJ, Hoteit I, McQuatters-Gollop A, Reid PC, et al From silk to satellite: half a century of ocean colour anomalies in the Northeast Atlantic. Global Change Biology. 2014;20:2117–2123. 10.1111/gcb.12457 24804626

[23] Joint I, Pomroy A. Phytoplankton biomass and production in the southern North Sea. Marine Ecology Progress Series. 1993;99:169–182. 10.3354/meps099169

[24] McClain CR. A Decade of Satellite Ocean Color Observations. Annual Review of Marine Science. 2009;1:19–42. 10.1146/annurev.marine.010908.163650 21141028

[25] Reid PC, Borges M, Svendsen E. A regime shift in the North Sea circa 1988 linked to changes in the North Sea horse mackerel fishery. Fisheries Research. 2001;50:163–171. 10.1016/S0165-7836(00)00249-6

[26] Beaugrand G, Reid PC. Long-term changes in phytoplankton, zooplankton and salmon related to climate. Global Change Biology. 2003;9:801–817. 10.1046/j.1365-2486.2003.00632.x

[27] Mollmann C, Diekmann R. Marine ecosystem regime shifts induced by climate and overfishing: A review for the northern hemisphere. Advances in Ecological Research. 2012;47:303–347. 10.1016/B978-0-12-398315-2.00004-1

[28] Viboud C, Bjørnstad ON, Smith DL, Simonsen L, Miller MA, Grenfell BT. Synchrony, waves, and spatial hierarchies in the spread of influenza. Science. 2006;312:447–451. 10.1126/science.1125237 16574822

[29] Keitt TH. Coherent ecological dynamics induced by large-scale disturbance. Nature. 2008;454:331–335. 10.1038/nature06935 18633416

[30] Vasseur DA, Gaedke U. Spectral analysis unmasks synchronous and compensatory dynamics in plankton communities. Ecology. 2007;88:2058–2071. 10.1890/06-1899.1 17824437

[31] Colebrook JM. Sea surface temperature and zooplankton, North Sea, 1948 to 1983. Journal du Conseil Conseil International pour l’Exploration de la Mer. 1985;42:179–185. 10.1093/icesjms/42.2.179

[32] Addison PS. The Illustrated Wavelet Transform Handbook: Introductory Theory and Applications in Science, Engineering, Medicine and Finance. New York: Taylor and Francis; 2002.

[33] Beaugrand G. Decadal changes in climate and ecosystems in the North Atlantic Ocean and adjacent seas. Deep-Sea Research Part II, Topical Studies in Oceanography. 2009;56:656–673. 10.1016/j.dsr2.2008.12.022

[34] Kirby RR, Beaugrand G, Lindley JA, Richardson AJ, Edwards M, Reid PC. Climate effects and benthic–pelagic coupling in the North Sea. MARINE ECOLOGY PROGRESS SERIES. 2007;330:31–38. 10.3354/meps330031

[35] Lindley JA, Beaugrand G, Luczak C, Dewarumez JM, Kirby RR. Warm-water decapods and the trophic amplification of climate in the North Sea. Biological Letters. 2010;6:773–776. 10.1098/rsbl.2010.0394 PMC3001376 20554562

[36] Wiltshire KH, Malzahn AM, Wirtz K, Greve W, Janisch S, Mangelsdorf P, et al Resilience of North Sea phytoplankton spring bloom dynamics: An analysis of long-term data at Helgoland Roads. Limnology and Oceanography. 2008;53:1294–1302. 10.4319/lo.2008.53.4.1294

[37] Walter JA, Sheppard LW, Anderson TL, Kastens JH, Bjørnstad ON, Liebhold AM, et al The geography of spatial synchrony. Ecology Letters. 2017;20:801–814. 10.1111/ele.12782 28547786

[38] Sheppard LW, Reid PC, Reuman DC. Rapid surrogate testing of wavelet coherences. European Physical Journal Nonlinear Biomedical Physics. 2017;5:1–9.

[39] Grenfell BT, Bjørnstad ON, Kappey J. Traveling waves and spatial hierarchies in measles epidemics. Nature. 2001;414:716–723. 10.1038/414716a 11742391

[40] Cazelles B, Chavez M, Berteaux D, Ménard F, Vik JO, Jenouvrier S, et al Wavelet analysis of ecological timeseries. Oecologia. 2008;156:287–304. 10.1007/s00442-008-0993-2 18322705

[41] Ohman MD, Runge JA. Sustained fecundity when phytoplankton resources are in short supply: omnivory by *Calanus finmarchicus* in the Gulf of St. Lawrence. Limnology and Oceanography. 1994;39:21–36. 10.4319/lo.1994.39.1.0021

[42] Nejstgaard JC, Gismervik I, Solberg PT. Feeding and reproduction by *Calanus finmarchicus*, and microzooplankton grazing during mesocosm blooms of diatoms and the coccolithophore *Emiliania huxleyi*. Marine Ecology Progress Series. 1997;147:197–217. 10.3354/meps147197

[43] Melle W, Runge J, Head E, Plourde S, Castellani C, Licandro P, et al The North Atlantic Ocean as habitat for *C*alanus finmarchicus: Environmental factors and life history traits. Progress in Oceanography. 2014;129:244–284. 10.1016/j.pocean.2014.04.026

[44] Head EJH, Ringuette M. Variability in Calanus finmarchicus egg production rate measurements: methodology versus reality. Journal of Plankton Research. 2017;39:645–663. 10.1093/plankt/fbx022

[45] King AA, Nguyen D, Ionides EL. Statistical inference for partially observed Markov processes via the R package pomp. Journal of Statistical Software. 2016;69:1–43. 10.18637/jss.v069.i12

[46] King AA, Ionides EL, Pascual M, Bouma MJ. Inapparent infections and cholera dynamics. Nature. 2008;454:877–880. 10.1038/nature07084 18704085

[47] Post E, Forchhammer M. Spatial synchrony of local populations has increased in association with the recent Northern Hemisphere climate trend. PNAS. 2004;101:9286–9290. 10.1073/pnas.0305029101 15197267 PMC438969

[48] Koenig W, Liebhold A. Temporally increasing spatial synchrony of North American temperature and bird populations. Nature Climate Change. 2016;6:614–617. 10.1038/nclimate2933

[49] Shestakova T, Gutiérrez E, Kirdyanov A, Camarero J, Génova M, Knorre A, et al Forests synchronize their growth in contrasting Eurasian regions in response to climate warming. PNAS. 2016;113:662–667. 10.1073/pnas.1514717113 26729860 PMC4725483

[50] Black B, van der Sleen P, Di Lorenzo E, Griffin D, Sydeman W, Dunham J, et al Rising synchrony controls western North American ecosystems. Global Change Biology. 2018;. 10.1111/gcb.14128 29575413

[51] Grünbaum D. Predicting availability to consumers of spatially and temporally variable resources. Hydrobiologia. 2002;480:175–191. 10.1023/A:1021296103358

[52] Prarie J, Sutherland K, Nickols K, Kaltenberg A. Biophysical interactions in the plankton: a cross-scale review. Limnology and Oceanography: Fluids and Environments. 2012;2:121–145.

[53] Colebrook JM, Robinson GA. Continuous Plankton Records: Seasonal cycles of phytoplankton and copepods in the North-Eastern Atlantic and the North Sea. Bulletins of Marine Ecology. 1965;6:123–139.

[54] Batten SD, Clark R, Flinkman J, Hays G, John E, John AWG, et al CPR sampling: the technical background, materials and methods, consistency and comparability. Progress In Oceanography. 2003;58(2-4):193–215. 10.1016/j.pocean.2003.08.004

[55] Raitsos DE, Reid PC, Lavender SJ, Edwards M, Richardson AJ. Extending the SeaWiFS chlorophyll data set back 50 years in the northeast Atlantic. Geophysical Research Letters. 2005;32:L06603 10.1029/2005GL022484

[56] Sakia RM. The Box-Cox transformation technique: a review. The Statistician. 1992;41:169–178. 10.2307/2348250

