# Supplementary material for: Synchrony is more than its top-down and climatic parts: interacting Moran effects on phytoplankton in British seas
Source: PLoS Comput Biol. 2019 Mar 28;15(3):e1006744. doi: 10.1371/journal.pcbi.1006744 (PMC6438443; doi:10.1371/journal.pcbi.1006744)
Supplement: S1 Text — (PDF) [file pcbi.1006744.s001.pdf]

Synchrony is more than its top-down and climatic parts: interacting Moran effects on phytoplankton in British seas: Supporting information

L. W. Sheppard, E. J. Defriez, P. C. Reid, D. C. Reuman

Appendix S1 Details of the numerical examples

For the first example given in the Introduction, suppose $\alpha(n,t)$ and $\beta(n,t)$ are biotic or abiotic environmental random variables in locations $n = 1, 2$ at times t, and assume these are independent through time and standard-normally distributed for all n and t. If a population index $\gamma(n,t)$ follows a stationary autoregressive (AR) process such as $\gamma(n,t+1) = c\gamma(n,t)+\alpha(n,t)$ for $n = 1, 2$, then by the Moran theorem [Moran, 1953], the population correlation through time $\text{cor}(\gamma(1), \gamma(2))$ equals $\text{cor}(\alpha(1), \alpha(2))$. If instead $\gamma(n,t+1) = c\gamma(n,t) + a\alpha(n,t) + b\beta(n,t)$, then the Moran theorem implies

$$\text{cor}(\gamma(1), \gamma(2)) = \text{cor}(a\alpha(1) + b\beta(1), a\alpha(2) + b\beta(2)) \tag{1}$$

$$= \frac{a^2\text{cov}(\alpha(1), \alpha(2)) + ab\text{cov}(\alpha(1), \beta(2)) + ab\text{cov}(\beta(1), \alpha(2)) + b^2\text{cov}(\beta(1), \beta(2))}{\sqrt{a^2 + 2ab\text{cov}(\alpha(1), \beta(1)) + b^2}\sqrt{a^2 + 2ab\text{cov}(\alpha(2), \beta(2)) + b^2}}. \tag{2}$$

Thus the population correlation depends not only on covariances $\text{cov}(\alpha(1), \alpha(2))$ or $\text{cov}(\beta(1), \beta(2))$ of α or β between locations (synchrony of the environmental variables), but also on covariances $\text{cov}(\alpha(n), \beta(n))$, and hence includes interaction effects between these Moran drivers. An AR(N) process could be used instead of the AR(1) process used above, with similar result.

We work out some specific examples. Suppose first that β is independent of α, $a = 1$, $b = 1$, $\text{cov}(\alpha(1), \alpha(2)) = 0.9$, and $\text{cov}(\beta(1), \beta(2)) = 0.5$. Then it is straightforward to show $\text{cor}(\gamma(1), \gamma(2)) = 0.7$. If again $a = 1$, $b = 1$, $\text{cov}(\alpha(1), \alpha(2)) = 0.9$, and $\text{cov}(\beta(1), \beta(2)) = 0.5$, but now $\text{cov}(\alpha(1), \beta(1)) = 0.5$, $\text{cov}(\alpha(2), \beta(2)) = 0.5$, $\text{cov}(\alpha(1), \beta(2)) = 0.5$, $\text{cov}(\alpha(2), \beta(1)) = 0.5$, then $\text{cor}(\gamma(1), \gamma(2)) = 0.8$. Thus interactions between Moran drivers in the second example are responsible for a 0.1 increase in population synchrony.

We now present supplementary details of our second example, from Results, the more sophisticated and detailed example making use of phase-shifted relationships between variables and timescale-specific synchrony, and showing explicitly how interaction effects can increase or decrease synchrony.

In the main text we defined $\gamma_S^{(3)}(n,t) = -(\alpha(n,t) + \beta(n,t-5))/f$, where $f = \sqrt{2 + \frac{1}{2}\cos(\varphi\pi)}$ was chosen so that the variance through time of $\gamma_S^{(3)}(n,t)$ is 1, the same as the variance through time of $\gamma_S^{(1)}$ and $\gamma_S^{(2)}$. We know $\text{var}_t(\gamma_S^{(1)}(n,t)) \approx \text{var}_t(\gamma_S^{(2)}(n,t)) \approx 1$ because $\alpha(n,t)$ and $\beta(n,t)$ were constructed to

have variance 1. We know

$$
\begin{aligned}
\mathrm{var}_t[-\alpha(n,t)-\beta(n,t-5)] &= \mathrm{var}_t[-\sqrt{\frac{1}{4}}(\alpha_S(n,t)+\beta_S(n,t-5))-\sqrt{\frac{3}{4}}(\alpha_L(n,t)+\beta_L(n,t-5))] && (3)\\
&\approx \frac{1}{4}\mathrm{var}_t[\alpha_S(n,t)+\beta_S(n,t-5)]+\frac{3}{2} && (4)\\
&\approx \frac{1}{2}\mathrm{var}_t[\sin(wt)+\cos(wt-\phi\pi-\frac{\pi}{2})]+\frac{3}{2} && (5)\\
&= \frac{1}{2}[\mathrm{var}_t(\sin(wt))+\mathrm{var}_t(\cos(wt-\phi\pi-\frac{\pi}{2})) && (6)\\
&+ 2\mathrm{cov}_t(\sin(wt),\cos(wt-\phi\pi-\frac{\pi}{2}))]+\frac{3}{2} && (7)\\
&\approx 2+\mathrm{cov}_t[\sin(wt),\cos(wt-\phi\pi-\frac{\pi}{2})] && (8)\\
&= 2+\mathrm{cov}_t[\sin(wt),\sin(wt-\phi\pi)] && (9)\\
&= 2+\frac{1}{2}\cos(\phi\pi), && (10)
\end{aligned}
$$

where the last equality follows from lemma 3. So $\mathrm{var}_t(\gamma_S^{(3)}(n,t)) \approx 1$.

Appendix S2 Thoughts and uncertainties on future patterns of PCI synchrony

C. finmarchicus is in decline in UK seas because it is a cold-water species and is shifting northward with climatic warming [Beaugrand and Reid, 2003]; it is being replaced by *C. helgolandicus*. Our long-timescale wavelet models can be used to highlight uncertainties in what the consequences of this change may be for long-timescale PCI synchrony. *C. finmarchicus* is a synchronising variable for PCI in the sense that if it were completely asynchronous our model of PCI would have lower synchrony (Fig. 3, magenta line). However, if the contribution of *C. finmarchicus* to PCI variability were reduced to zero with no other changes to the plankton system, PCI should become more synchronous because the main driver of PCI variability would then be temperature alone, and temperature is highly synchronous (Fig. S3). This projection is based on setting the *C. finmarchicus* coefficient $\beta_k(\sigma)$ (see Eq. 1 in the main text) to zero in our best long-timescale model. This result may have value as a projection given the assumptions involved, but it likely has little value as a prediction of future events since reductions in *C. finmarchicus* have already been associated with large-scale changes in the plankton community [Beaugrand and Reid, 2003, Beaugrand et al., 2003]. Relative to *C. finmarchicus*, *C. helgolandicus* had different phase relationships with both PCI and growing season temperature (details not shown), so even if *C. helgolandicus* replaced *C. finmarchicus* with no other changes to the community (still an inaccurate idealization) one could expect major changes in PCI synchrony stemming from both changes in direct top-down Moran effects and changes in interactions between Moran effects.

Appendix S3 Data

All measurements of biological and physical variables, which were made at irregularly spaced but numerous times and locations, were compiled into time series representing the changing values found inside 2 by 2 degree areas of sea (26 different areas spread around the UK; Fig. S4). Where measurements of a variable were available for a given month in a given year within a given 2 by 2 degree area, they were averaged to obtain a single value for that location and month. Where no readings were available, an average value across other years for that calendar month and location was used to fill the gap. The values in this filled monthly time series were then averaged within each year to obtain annualized time series for each of the

variables in each of the 26 areas. Physical variables were sampled independently of biological variables but assembled into time series by the same procedures, except that for biological variables all 12 months were included in a yearly average, whereas for physical variables we explored using averages over different sets of months corresponding to different seasons (table 2) which might coincide with the growing season of certain plankton groups.

As stated in the main text, PCI samples are categorized as No Green (NG), Very Pale Green (VPG), Pale Green (PG) and Green (G) by visual comparison. Colebrook and Robinson [1965] gives the dilution factors needed to render green samples with different colors the same color (VPG 1, PG 2, G 6.5), but alternatives are available. Raitsos et al. [2013] obtains NG 0, VPG 1, PG 2.4 and G 4.6 for ratios of chlorophyll α obtained from samples of different color categories. They note that using these values instead of the dilutions of Colebrook and Robinson [1965] produces little change in time series. Raitsos et al. [2013] also quotes the satellite data comparison of Raitsos et al. [2005], giving the following SeaWiFS chlorophyll α levels for each CPR category: NG 1.15, VPG 1.34, PG 2.06, G 3.92 mg/m^3, with some caveats. We used the original dilution factors of Colebrook and Robinson [1965] to construct monthly time series by taking the mean of available samples in each area in each month in each year.

The biological variables to be compared with the PCI index were zooplankton groups sampled simultaneously with PCI. Individual samples were averaged into monthly time series in the same way as for PCI. Of the very many plankton groups counted in the CPR survey, the species we analyzed were chosen prior to any analyses for their commonness and likely importance for plankton ecology and human concerns.

Two degree summaries of monthly sea surface temperature, cloud and wind data were drawn from the International Comprehensive Ocean-Atmosphere Data Set (ICOADS), release 2.4, in Monthly Summary Groups (MSG1) format. Cloud cover was recorded as proportion of the sky area that was observed to be obscured by cloud, discretized into oktas, or eighths, and was included because of its likely influences on sunlight levels, temperature and salinity variation (related to freshwater input/output). Temperature measurements from the top ten meters of the water column were included, as was wind speed at the sea surface. Salinity sample data was downloaded from `http://www.nodc.noaa.gov/OC5/SELECT/dbsearch/dbsearch.html` on 16 January 2015. Individual samples from the top ten meters of the water column were averaged into monthly time series.

Areas in which PCI samples were unavailable for two or more consecutive years at a time were excluded. The choice of 2 by 2 degree areas was a compromise between spatial resolution and the number of samples available within a typical area to produce useable plankton time series. In the 26 useable areas, CPR data were missing for an average of 2.9 months from each year. For each zooplankton group and for PCI, values for these months were filled with the median value of the time series in that month in that location in other years. The median value was used because plankton data were non-normally distributed (see Appendix S4). Physical data were compiled from monthly time series in the same way, except that when missing months were filled in, the mean value in that month in that location in other years was used instead of the median that was used for the plankton time series. The mean value was used because the physical variables were close to normally distributed (see Appendix S4). For sea surface temperature, over all 26 useable areas an average of only 0.032 months were missing from each year. For cloud cover data, over all 26 useable areas an average of only 0.041 months were missing from each year. For wind speed data, over all 26 useable areas an average of only 0.023 months were missing from each year. For salinity data, over all 26 useable areas an average of 5.3 months were missing for each year. Thus our salinity variables were the least reliable time series used. Other physical variables (e.g., nutrient concentrations) were excluded from our analysis due to even fewer monthly values.

Appendix S4 Box-Cox transformations

For each variable the 26 annualized time series were subjected to Box-Cox normalization. This avoids variability in each data set being dominated by spikes corresponding to outbreaks. Population data

were roughly log-normally distributed. We used a version of the standard optimal Box-Cox transformation technique [Sakia, 1992] to render the marginals of our time series as close to normal as possible. Fourier surrogate techniques (Appendix Appendix S8) produce data with normal marginals [Schreiber and Schmitz, 2000], so significance testing based on statistics derived from these surrogates is only fair if the data themselves are normally distributed.

For each variable at each site we found the optimal Box-Cox transformation using the following procedure. As the transformation is only possible for strictly positive values, standard Box-Cox transformations of population data involve the addition of a constant to each variable to give a minimum value of one. We call this artificial minimum the ‘offset’. Since some of our variables are not population densities but physical variables we allowed for a range of possible best offsets when selecting the best Box-Cox coefficient. We began with a starting offset equal to the minimum value of the time series (i.e. no constant was added), or if the minimum was ≤ 0, equal to the minimum increment found between any two successive ordered values. We transformed the time series using a range of 400 candidate Box-Cox coefficients from -2 to +2, and found the maximum likelihood variance, mean, and slope parameters for a model of the transformed variable with a Gaussian distribution about a linear trend. We then repeated this exercise with a range of 100 offset values decreasing in five percent increments from the starting offset, plus 100 more values increasing in five percent increments from the starting offset. We recorded the log maximum likelihood value found using each candidate offset and Box-Cox coefficient. The offset and coefficient giving the greatest mean log likelihood were selected as optimal and the corresponding Box-Cox transformation was applied to the time series. The linear trend was subtracted from each time series, and the variance re-scaled to one. Thus for each time series we obtained zero-mean time series with no trend and distributions as close to Gaussian as possible for a transformation in the Box-Cox family.

The mean (over locations) of the Box-Cox coefficients found for time series of each variable is given in table S2. Box Cox coefficients for biological variables tended to be around 0, for physical variables around 1, indicating that the raw physical values were approximately normally distributed and biological counts were approximately log normal prior to Box-Cox transformation.

Appendix S5 The wavelet transform and normalizations

If $x(t)$ ($t = 1, \dots, T$) is a time series, we denote its *wavelet transform* at timescale (period) σ and time t by $W_\sigma(t)$. Wavelets are increasingly common in ecology [Grenfell et al., 2001, Viboud et al., 2006, Keitt and Fischer, 2006, Cazelles et al., 2008, Rouyer et al., 2008, Keitt, 2008, Bell et al., 2012, Cazelles et al., 2014, Sheppard et al., 2016] and general introductions are available [Torrence and Compo, 1998, Addison, 2002]. We used a continuous complex Morlet wavelet transform [Addison, 2002]. The mother wavelet is $\Psi(t) = (e^{i2\pi f_0 t} - e^{-(2\pi f_0)^2/2})\exp(-t^2/2)$, with $f_0 = 0.5$. Wavelets associated with a range of timescales were produced using a rescaling technique: $\Psi_\sigma(t) = s^{-1/2}(e^{i2\pi f_0 t/s} - e^{-(2\pi f_0)^2/2})\exp(-t^2/2s^2)$; following earlier convention [Cazelles et al., 2014] we identify each wavelet with a timescale, $\sigma = s/f_0$, and frequency, $f = f_0/s$. Convolving the time series $x(t)$ with wavelets having different periods produces a set of complex components $W_{n,\sigma}(t) = \sum_{t'} x_n(t+t')\Psi_\sigma(t')$ which comprise the wavelet transform of $x(t)$. Wavelets with periods from two years to over 26 years were generated, starting with $\sigma = 2$ and multiplying each period by 1.05 to get the next. The longest timescale used for the data of this study was 26.5499 years. The transform is scalloped to remove poorly estimated values [Addison, 2002], specifically where the wavelet envelope at the time series edge in the convolution has 50% or more of its maximum amplitude. The scalloping procedure determines the shape of the useable wavelet transform values by removing the unreliable edge values, with the high-frequency end having the most useable values and the low-frequency end having fewer useable values due to the wider wavelets overhanging the edge of the time series in the convolutions that define the wavelet transform. Thus the plots are 'scallop-shaped'. The center frequency f_0 of the mother wavelet, which has width $s = 1$ and $\sigma = 2$, was taken to be 0.5 to give a high degree of temporal resolution, but making necessary the subtraction of a constant to keep the mean of the wavelet equal to zero [Addison, 2002]. The mother wavelet was scaled so that one oscillation

equaled two years, i.e., $\sigma = 2$, because a two-year period is the highest-frequency fluctuation that can be identified in an annual time series. The actual peak in the Morlet wavelet power spectrum of a sinusoidal signal with frequency f' and period $\sigma' = 1/f'$ is at $s = ((2\pi f_0 + (2 + (2\pi f_0)^2)^{1/2})/4\pi)(\sigma')$, so $f \approx f'$ only [Meyers et al., 1993].

If $x_n(t)$ $(t = 1, \ldots, T)$ is the nth of N time series from different locations, and $W_{n,\sigma}(t)$ is its wavelet transform, then we refer to

$$w_{n,\sigma}(t) = W_{n,\sigma}(t)/|W_{n,\sigma}(t)|$$

as the *phasor-normalized transform.* We also define

$$w_{n,\sigma}(t) = \frac{W_{n,\sigma}(t)}{\sqrt{\frac{1}{NT}\sum_{n=1}^{N}\sum_{t=1}^{T} W_{n,\sigma}(t)\overline{W_{n,\sigma}(t)}}},$$

where overbar denotes complex conjugation, as the *power-normalized transform*, using the same notation, $w_{n,\sigma}(t)$, as was used for the phasor-normalized transforms. The normalization used in any given context will be specified textually if it is not immediately clear. The terminology *power-normalized transform* was chosen because the denominator in the expression for the power-normalized transforms $w_{n,\sigma}(t)$ is the square root of the average wavelet power of time series over locations. That denominator is only a single positive rescaling factor, so the $w_{n,\sigma}(t)$ contain essentially the same information as the $W_{n,\sigma}(t)$. The phases of $w_{n,\sigma}(t)$ and $W_{n,\sigma}(t)$ are the same, and are equal to the phase of oscillation in $x_n(t)$ at time t and timescale σ.

Appendix S6 Spatial coherence

The *spatial coherence* of two variables $x_n^{(0)}(t)$ and $x_n^{(1)}(t)$ is the magnitude of the quantity

$$\Pi_\sigma^{(01)} = \frac{1}{NT}\sum_{n=1}^{N}\sum_{t=1}^{T} w_{n,\sigma}^{(0)}(t)\overline{w_{n,\sigma}^{(1)}(t)}, \tag{11}$$

where the w are power-normalized transforms (Appendix S5). The spatial coherence takes values between 0 and 1 (lemma 4). Because $w_{n,\sigma}^{(0)}(t)\overline{w_{n,\sigma}^{(1)}(t)}$ is a complex number with phase equal to the phase difference between the two wavelet components, the sum over n and t is large if the phase difference is consistent over time and locations, and small otherwise. Wavelet components can also have varying magnitudes, and spatial coherence is larger still if there are correlations in amplitudes of fluctuations. For $N = 1$, the spatial coherence is the (standard) wavelet coherence [Torrence and Compo, 1998, Addison, 2002]. The measure also relates to the phase coherence which is defined for a single pair of transforms ($N = 1$) [Grenfell et al., 2001, Bandrivskyy et al., 2004, Viboud et al., 2006, Stefanovska, 2007], using the same formula but with phasor-normalized transforms (Appendix S5). The phase of (11) is the average phase difference between the variables, as a function of σ.

Appendix S7 Linear models for wavelet transforms

To understand potential causes of synchrony in the $x_n^{(0)}(t)$ we use complex linear models expressing the transforms, $w_{n,\sigma}^{(0)}(t)$, of the $x_n^{(0)}(t)$ as nearly as possible in terms of the transforms, $w_{n,\sigma}^{(k)}(t)$, of the $x_n^{(k)}(t)$:

$$w_{n,\sigma}^{(0)}(t) \approx \beta_1(\sigma)w_{n,\sigma}^{(1)}(t) + \cdots + \beta_K(\sigma)w_{n,\sigma}^{(K)}(t). \tag{12}$$

We use power-normalized transforms in this section. The coefficient $\beta_k(\sigma)$ is a complex number with phase equal to the phase difference between the fluctuations in $x_n^{(k)}(t)$ at timescale σ and the contribution of this potential driver of dynamics to the fluctuations in $x_n^{(0)}(t)$ at the same timescale. The magnitude

of $\beta_k(\sigma)$ is the corresponding 'gain', i.e., the strength of the contribution of the driver at timescale σ to $x_n^{(0)}(t)$, relative to the strength of the oscillation at that timescale in the driver itself.

For a frequency component σ, we consider the space $\mathbb{C}^D$ where D is the product of N and the number of time steps, t, for which the wavelet components $w_{n,\sigma}^{(0)}(t)$ and $w_{n,\sigma}^{(k)}(t)$ are defined after scalloping to remove poorly estimated values (Appendix S5). The components $w_{n,\sigma}^{(0)}(t)$ for $n = 1, \ldots, N$ combine naturally to form a point in this space, as do the components $w_{n,\sigma}^{(k)}(t)$, $n = 1, \ldots, N$, for any k. The space $\mathbb{C}^D$ is a Hilbert space under the inner product $\langle w_{n,\sigma}^{(0)}(t), w_{n,\sigma}^{(k)}(t)\rangle = \frac{1}{NT}\sum_n \sum_t w_{n,\sigma}^{(0)}(t)\overline{w_{n,\sigma}^{(k)}(t)}$, and we denote the associated norm by $\|\cdot\|$.

Applying lemma 5 with $V = \mathrm{Span}(w_{n,\sigma}^{(1)}(t), \ldots, w_{n,\sigma}^{(K)}(t))$, there is a unique $v \in V$ such that $w_{n,\sigma}^{(0)}(t) = v + d$, where $d \perp V$. The point v is an approximation of $w_{n,\sigma}^{(0)}(t)$, in the sense that the residuals, d, have minimal magnitude, $\|d\|$ (lemma 5). The point v also has maximal spatial coherence with $w_{n,\sigma}^{(0)}(t)$, in the sense that, for any other $w \in V$, the spatial coherence, $\frac{|\langle w_{n,\sigma}^{(0)}(t), w\rangle|}{\|w\|}$, of $w_{n,\sigma}^{(0)}(t)$ with the power-normalization of w is less than or equal to the spatial coherence, $\frac{|\langle w_{n,\sigma}^{(0)}(t), v\rangle|}{\|v\|}$, of $w_{n,\sigma}^{(0)}(t)$ with the power-normalization of v. This follows from lemma 5.

The point v can be written explicitly as a complex linear model expressing the transforms $w_{n,\sigma}^{(0)}(t)$ as nearly as possible in terms of the transforms $w_{n,\sigma}^{(k)}(t)$ by writing $v = \beta_1 w_{n,\sigma}^{(1)}(t) + \cdots + \beta_K w_{n,\sigma}^{(K)}(t)$, where $\beta = (\beta_1, \ldots, \beta_K)^t$ is a solution of the equation $M\beta = p$, with the ij^{th} component of the matrix M equal to $\langle w_{n,\sigma}^{(j)}(t), w_{n,\sigma}^{(i)}(t)\rangle$ (here $1 \leq i, j, \leq K$) and the jth component of p equal to $\langle w_{n,\sigma}^{(0)}(t), w_{n,\sigma}^{(j)}(t)\rangle$ (lemma 5). We know a solution of this equation exists because $v \in V = \mathrm{Span}(w_{n,\sigma}^{(1)}(t), \ldots, w_{n,\sigma}^{(K)}(t))$. The solution will be unique if and only if the $w_{n,\sigma}^{(k)}(t)$ for $k = 1, \ldots, K$ are linearly independent. Solutions to the equation $M\beta = p$ (ie. complex wavelet coefficients β for each frequency) were calculated by means of the left matrix divide operation in Matlab for numerical stability.

Appendix S8 Surrogates

Starting from a spatiotemporal dataset $x_n(t)$ for $n = 1, \ldots, N$ and $t = 1, \ldots, T$, we used two types of artificial 'surrogate' (i.e., resampled) data, *spatially synchronous surrogates* and *asynchronous surrogates*. Surrogate approaches to significance testing for wavelet analyses are standard [Theiler et al., 1992, Prichard and Theiler, 1994, Schreiber and Schmitz, 2000, Rouyer et al., 2008, Cazelles et al., 2014]. Both procedures provide synthetic spatiotemporal data for the same number of locations over the same times as the original data, with certain features of the original data randomized away and other features retained. Both procedures first Fourier transform the $x_n(t)$, then randomize the phases of the Fourier components by adding a random uniformly distributed phase at each frequency, and finally inverse Fourier transform to obtain the surrogate data. Thus both procedures are based on the standard Fourier surrogate approach [Schreiber and Schmitz, 2000]. These steps result in time series that have the same power spectrum as the original time series, and therefore the same autocorrelation properties, but have different temporal structure [Schreiber and Schmitz, 2000]. Box-Cox transformations were performed (Appendix S4) to normalize data during the data preparation stage, in part because Fourier surrogates tend to normalize time series marginal distributions [Schreiber and Schmitz, 2000]. Comparisons are therefore fairer between statistics calculated for surrogate and real data when the real data has been pre-normalized. For spatially synchronous surrogates, for each timescale σ the same phase is added to the Fourier components at σ for each of the $x_n(t)$. Thus surrogates also preserve cross-spectrum and cross-correlation properties of the time series. This is the approach developed by Prichard and Theiler [1994]. For asynchronous surrogates, independent phases are added for each $x_n(t)$, so that synchrony between the $x_n(t)$ is eliminated at all timescales.

Appendix S9 Model construction

We developed a significance test for adding variables to the linear model constructed in Appendix S7. Consider adding another variable to an explanatory model consisting of K variables. If $x_n^{(k)}(t)$ for $k = 0, \ldots, K+1$ are variables measured at locations $n = 1, \ldots, N$ and times $t = 1, \ldots, T$, then we can use spatially synchronous surrogates to determine whether the linear model

$$w_{n,\sigma}^{(0)}(t) \approx \beta_1(\sigma) w_{n,\sigma}^{(1)}(t) + \cdots + \beta_{K+1}(\sigma) w_{n,\sigma}^{(K+1)}(t) \tag{13}$$

is significantly better than the linear model

$$w_{n,\sigma}^{(0)}(t) \approx \alpha_1(\sigma) w_{n,\sigma}^{(1)}(t) + \cdots + \alpha_K(\sigma) w_{n,\sigma}^{(K)}(t). \tag{14}$$

The resulting test, now described in detail, is the wavelet linear model analogue of standard F-tests used to compare nested models in a classic general linear modeling framework.

Using the notation of Appendix S7 and $V = \text{Span}(w_{n,\sigma}^{(1)}(t), \ldots, w_{n,\sigma}^{(K+1)}(t))$, first $w_{n,\sigma}^{(0)}(t)$ is written as $v_{\text{data}} + d_{\text{data}}$ for each timescale, σ. Then define $w_{j,n,\sigma}^{(K+1)}(t)$ as the power-normalized transforms of a jth synchrony-preserving surrogate of $x_n^{(K+1)}(t)$ for $j = 1, \ldots, 10000$, and write $w_{n,\sigma}^{(0)}(t)$ as $v_j + d_j$ for $V = \text{Span}(w_{n,\sigma}^{(1)}(t), \ldots, w_{n,\sigma}^{(K)}(t), w_{j,n,\sigma}^{(K+1)}(t))$ for each timescale, σ. Thus we have $d_{\text{data},\sigma}$ and $d_{j,\sigma}$ for $j = 1, \ldots, 10000$, making the σ dependence explicit. We consider that the model (13) is a significant improvement over the model (14), for timescale σ at significance level α, if $\|d_{\text{data},\sigma}\|^2$ is smaller than a fraction $1-\alpha$ of the $\|d_{j,\sigma}\|^2$. In words, the more complex model is considered better at σ if the additional predictor allows a reliably greater improvement in the representation of $w_{n,\sigma}^{(0)}(t)$ than would a randomized surrogate data set with the same spatial and temporal autocorrelation properties.

The requirement that $\|d_{\text{data},\sigma}\|^2$ is smaller than a fraction $1-\alpha$ of the $\|d_{j,\sigma}\|^2$ at timescale σ is equivalent to the requirement that the spatial coherence at σ between the right side of (13) and $w_{n,\sigma}^{(0)}(t)$ is greater when the right side of (13) is fitted using the actual $w_{n,\sigma}^{(K+1)}(t)$ than it is when fitted using any of a fraction $1-\alpha$ of the surrogate transforms $w_{j,n,\sigma}^{(K+1)}(t)$ in place of $w_{n,\sigma}^{(K+1)}(t)$. Hence the more complex model is considered better at σ if the additional predictor improves the spatial coherence of the model with $w_{n,\sigma}^{(0)}(t)$ more than would a randomized, surrogate predictor. To see this, we write the squares of the spatial coherences to be compared as

$$\frac{|\langle w_{n,\sigma}^{(0)}(t), v_{\text{data}}\rangle|^2}{\|v_{\text{data}}\|^2} = \frac{|\langle v_{\text{data}} + d_{\text{data}}, v_{\text{data}}\rangle|^2}{\|v_{\text{data}}\|^2} \tag{15}$$

$$= \frac{|\|v_{\text{data}}\|^2|^2}{\|v_{\text{data}}\|^2} \tag{16}$$

$$= \|v_{\text{data}}\|^2 \tag{17}$$

$$= \|w_{n,\sigma}^{(0)}(t)\|^2 - \|d_{\text{data}}\|^2 \tag{18}$$

and

$$\frac{|\langle w_{n,\sigma}^{(0)}(t), v_j\rangle|^2}{\|v_j\|^2} = \frac{|\langle v_j + d_j, v_j\rangle|^2}{\|v_j\|^2} \tag{19}$$

$$= \frac{|\|v_j\|^2|^2}{\|v_j\|^2} \tag{20}$$

$$= \|v_j\|^2 \tag{21}$$

$$= \|w_{n,\sigma}^{(0)}(t)\|^2 - \|d_j\|^2, \tag{22}$$

where (18) and (22) follow because $v_{\rm data} \perp d_{\rm data}$ and $v_j \perp d_j$. Thus the spatial coherence (15) is larger than the spatial coherence (19) for exactly the same j for which $\|d_{\rm data}\|^2$ is smaller than $\|d_j\|^2$.

The procedures described above give one statistical test for each timescale σ. But the results of the tests are not independent across values of σ, particularly for similar values. To determine whether (13) is a significant improvement over (14) for a whole band of timescales σ in aggregate, the rank of $\|d_{{\rm data},\sigma}\|^2$ relative to the surrogate values $\|d_{j,\sigma}\|^2$ was found for each timescale in the band (rank 1 was the lowest) and the mean rank was computed inside the band. The same procedure applied to each surrogate separately (i.e., ranking each $\|d_{j,\sigma}\|^2$ against the $\|d_{j',\sigma}\|^2$ for all $j' \neq j$) produced surrogate mean rank values for the timescale band, and the proportion of these mean ranks less than the actual mean rank provided a p-value for the band.

Two timescale bands, as opposed to three or more bands, were used for simplicity and because wavelet methods have finite capacity for resolving timescales of fluctuation in finite time series. A frequency of 1 cycle every 4 years was selected as the boundary between bands both because this is half the Nyquist frequency for annual sampling (1 cycle every 2 years), and because it is the boundary between persistent and anti-persistent behavior in Fourier components, as measured with lag-1 autocorrelation [Sheppard et al., 2016].

Statistical comparisons between (14) and models adding more than one predictor variable are straightforwardly possible via the same approach. Independent spatially synchronous surrogates are generated for each of the added variables.

The use of spatially synchronous surrogates is appropriate for the tests of this section because spatial and temporal autocorrelation properties of $x_n^{(K+1)}(t)$ can inflate spatial coherences between $x_n^{(K+1)}(t)$ and $x_n^{(0)}(t)$, producing small values of $\|d_{{\rm data},\sigma}\|^2$. Using spatially synchronous surrogates ensures that the surrogates of $x_n^{(K+1)}(t)$ have the same spatial and temporal autocorrelation properties as $x_n^{(K+1)}(t)$ itself, and the values $\|d_{j,\sigma}\|^2$ are subject to the same biases. Spatially synchronous surrogates represent the null hypothesis of no relationship between $x_n^{(0)}(t)$ and $x_n^{(K+1)}(t)$ while retaining the spectral properties of $x_n^{(K+1)}(t)$.

We used the above significance testing methods as part of a model selection and validation procedure for constructing optimal models of PCI transforms for long (>4 years) and short (<4 years) timescales, separately. None of the wavelet components we used had a scale of exactly 4 years. We examined all possible combinations of four variables or fewer for each timescale band separately. This choice to limit models to four variables or fewer followed a similar choice of Defriez and Reuman [2017a] and Defriez and Reuman [2017b]. The choice served to keep computation times reasonable, and was consistent with our desire to focus on only the most important determinants of plankton dynamics and synchrony. Models including more than one temperature variable, more than one salinity variable, more than one wind variable or more than one cloud cover index were discarded. Of the remaining models, and separately for long and short timescale, we retained only those models for which each variable included in the model significantly improved the fit over not including it (using the significance methods described above). This step ensured the selected model was not merely the best model among poor alternatives, but also explains a significant amount of variation in the response transform $w_{n,\sigma}^{(0)}(t)$ (this is what the word 'testing' in 'model selection and testing' refers to). Models for which not all variables could individually pass this test were discarded. This prevents the inclusion of redundant explanatory variables. Remaining models were ranked (again, separately for long and short timescales) via a leave-one-out cross validation criterion.

The leave-one-out cross validation was performed as follows. For each model remaining after the above steps, and for each of the 26 regions of British seas for which PCI time series and other time series and their transforms were available (figure S4), we re-fitted to obtain model parameters using the data in the other 25 locations, and then evaluated goodness of fit for the data in the omitted location. In other words, when leaving out location n' we wrote (using the notation of section Appendix S7) $w_{n\neq n',\sigma}^{(0)}(t)$ as $v_{\rm data} + d_{\rm data}$ and found the optimal (minimal $\|d_{{\rm data},\sigma}\|^2$) coefficients β_k for $n \neq n'$. We then found $\|d_{{\rm data},\sigma}\|^2$ at location n' using these coefficients. The average of $\|d_{{\rm data},\sigma}\|^2$ over σ in the desired frequency

band gave the leave-one-out goodness of fit for this location n' for the model. The lower the value the better the fit. We averaged over the 26 locations, left out in turn, to obtain a single index of leave-one-out goodness of fit for each model for the frequency band. The resulting measure was intended to be robust against overfitting by the addition of more variables. The remaining model with the best leave-one-out goodness of fit was selected at high frequencies, and separately at low frequencies.

Appendix S10 Checking phase relationships

The phases of the model coefficients $\beta_k(\sigma)$ are supposed to represent the phase lag of effects (or associations) at timescale σ of $x_n^{(k)}(t)$ on $x_n^{(0)}(t)$ (see Eq.2 from Methods for notation). Strong associations which are consistent over time and space should therefore produce a correspondence between the phase of $\beta_k(\sigma)$ and the average phase, over space and/or time, of $w_{n,\sigma}^{(0)}(t)\overline{w_{n,\sigma}^{(k)}(t)}$, where the overbar denotes complex conjugation; the phase of this quantity is the phase difference between $x_n^{(0)}(t)$ and $x_n^{(k)}(t)$ at timescale σ, time t, and location n. We tested for this phase correspondence and found it to be good (Fig. S1). Especially at long timescales, a particular phase relationship between predictor and PCI transforms is maintained consistently through time (Fig. S1a, b). PCI and temperature had a consistent phase relationship on long timescales, corresponding to a phase shift of one quarter of a cycle (Fig. S1a,e). The average phase difference between PCI and temperature transforms (average of the green line in Fig. S1e over long timescales) was 0.42π radians at long timescales ($\pi/2$ is exactly a quarter cycle). PCI and *C. finmarchicus* were found to be in a consistent anti-phase relationship (Fig. S1b,f). The average phase difference between PCI and *C. finmarchicus* transforms (average of the green line in Fig. S1f) was 0.78π radians for long timescales and 0.99π radians for short timescales, both of which were reasonably close to anti-phase (π radians phase difference). The average phase differences between PCI and echinoderm and decapod larvae, respectively, on short timescales were 0.09π and 0.01π radians, both of which are approximately in-phase relationships.

Appendix S11 Location permutations

For each predictor variable, the spatial coherence with PCI was computed, as was the spatial coherence between PCI and 1000 surrogate versions of the predictor in which time series locations were randomly permuted but time series were otherwise unmanipulated. For each timescale in the relevant band (long timescales for growing season temperature and *C. finmarchicus* abundance, short timescales for *C. finmarchicus* abundance and for echinoderm larvae and decapod larvae) the spatial coherence for the unmanipulated data was ranked in the distribution of surrogate spatial coherences and ranks were averaged across the timescale band. The same procedure was then applied to each surrogate spatial coherence separately, i.e., surrogate spatial coherences were then ranked in the other surrogate spatial coherences and these values were averaged for each surrogate across the timescale band. The proportion of the surrogate mean ranks less than the actual mean rank was then computed. On long timescales, mean spatial coherence rank was higher than that of 989 of 1000 such surrogates values for growing season temperature, and 1000 of 1000 for *C. finmarchicus*. At short timescales, mean spatial coherence rank was higher than that of 997 of 1000 such surrogates for *C. finmarchicus*, 1000 of 1000 for echinoderm larvae, and 988 of 1000 for decapod larvae.

These results are consistent with spatial inhomogeneity observed in the raw values of the PCI time series. Our constructed PCI monthly time series mean values (before the Box-Cox and other standardization), when averaged over all months and years, produced values that varied by location between a minimum of 0.6207 and a maximum of 2.1956 (mean of 1.1157 and standard deviation of 0.4187). These values do not have units because PCI is an index.

Appendix S12 Link to species-specific results

Spatial coherences between nine individual phytoplankton species abundances from the CPR data set and *C. finmarchicus* were tested in Sheppard et al. [2017]. Results were consistent with our findings that PCI and *C. finmarchicus* were significantly spatially coherent and in anti-phase. At short-timescales, three of the four *Ceratium* species tested were significantly spatially coherent ($p < 0.05$) with *C. finmarchicus*, and were in an approximate anti-phase relationship with it. Average phase shifts and p-values were: *Ceratium fusus*, 0.9581π, $p = 0.0088$; *Ceratium furca*, -0.9451π, $p = 0.0143$; *Ceratium macroceros*, -0.8356π, $p = 0.0372$. No other significant ($p < 0.05$) spatial coherences were found between phytoplankton species and *C. finmarchicus* at short or long timescales. Sheppard et al. [2017] used the same definitions of 'short' and 'long' timescales used here, and the same spatial grid.

Appendix S13 Mean fields

Given a spatiotemporal data set $x_n(t)$ for $n = 1, \ldots, N$ and $t = 1, \ldots, T$, and letting $w_{n,\sigma}(t)$ represent the phasor-normalized wavelet transforms (Appendix S5), we define the *wavelet phasor mean field* as

$$r_\sigma(t) = \frac{1}{N}\sum_{n=1}^{N} w_{n,\sigma}(t). \tag{23}$$

The magnitude of this quantity is between 0 and 1. The wavelet phasor mean field is one natural choice for a time- and timescale-specific measure of synchrony: if the phasors, $w_{n,\sigma}(t)$, are asynchronous their average tends to have small magnitude, as the phasors conduct a random walk in the complex plane when summed; but if phases are synchronized, the phasors produce a directed walk when summed, giving a large value for $|r_\sigma(t)|$. The value $|r_\sigma(t)| = 1$ indicates the time series have identical phases of oscillation at timescale σ and time t.

The significance of a wavelet phasor mean field magnitude $|r_\sigma(t)|$ at timescale σ and time t is obtained by comparing to a distribution of the mean field magnitude of N random phasors,

$$\left|\frac{1}{N}\sum_{n=1}^{N} \exp(2\pi u_n i)\right|, \tag{24}$$

where the u_n are independent uniformly distributed random variables on the unit interval. For large N, this is a Rayleigh distribution [Strutt, 1902]. For any N a distribution of possible magnitudes for the sum can quickly numerically generated by summing a large number of sets of N random unit phasors.

If phase synchrony occurs for σ and t, so that the unit phasors $w_{n,\sigma}(t)$ have similar phase for all or most $n = 1, \ldots, N$, then $|r_\sigma(t)|$ will tend to be larger than the vast majority of the random quantities of (24). The test applies for one pair of σ and t values at a time, so one must be cognizant of multiple testing errors when examining ranges of σ and t. The approach used here is the same as that used in Sheppard et al. [2013]. The significance of the wavelet phasor mean field of PCI time series is used to plot the significance contours of the phase synchrony depicted in figure 3a

We also define a *wavelet mean field*, another measure of synchrony which includes information about the magnitudes of oscillation in the $x_n(t)$ in addition to the phase-synchrony information on which the wavelet phasor mean field relies exclusively. The same equation (23) is used to define the wavelet mean field, but now with the $w_{n,\sigma}(t)$ representing power-normalized transforms (Appendix S5).

In addition to the reasoning that the magnitude of the the wavelet mean field will tend to be bigger when fluctuations at time t and timescale σ have similar phase at all sampling locations, and are therefore synchronized, the wavelet mean field is also a natural choice for a time- and timescale-specific measure of strength of synchrony because of its mathematical properties. The mean squared magnitude $\frac{1}{T}\sum_{t=1}^{T} |r_\sigma(t)|^2$ is between 0 and 1. We refer to this quantity as the *mean squared synchrony*. It equals 1

for all σ if and only if the time series $x_n(t)$ $(t = 1, \ldots, T)$ are identical (lemma 6). Also, this quantity is the power of the average time series divided by the average of the powers of all the time series (lemma 6). If time series are unsynchronized, power in the average time series will be reduced, as unsynchronized fluctuations will tend to cancel. In contrast, synchronized fluctuations will reinforce each other and contribute power to the average. In this way $\frac{1}{T}\sum_{t=1}^{T} |r_\sigma(t)|^2$ represents synchrony for each timescale, σ. It is a generalization of

$$\frac{\text{var}\left(\frac{1}{N}\sum_{n=1}^{N} x_n(t)\right)}{\frac{1}{N}\sum_{n=1}^{N} \text{var}(x_n(t))},$$

a familiar quantity interpretable as synchrony because it represents the extent to which fluctuations in local time series reinforce each other or cancel in the average (lemma 6). The wavelet mean field [Sheppard et al., 2016] and related methods [Grenfell et al., 2001, Viboud et al., 2006, Keitt and Fischer, 2006, Keitt, 2008] have been used previously to study synchrony in ecology.

Appendix S14 A Moran theorem for wavelet linear models

This section parallels the development of the wavelet Moran theorem in Sheppard et al. [2016], but this time incorporating multiple drivers of synchrony. If, as in previous sections, $x_n^{(0)}(t)$ and $x_n^{(k)}(t)$, $k = 1, \ldots, K$ are variables measured at locations $n = 1, \ldots, N$ and times $t = 1, \ldots, T$, $w_{n,\sigma}^{(0)}(t)$ and $w_{n,\sigma}^{(k)}(t)$ are the corresponding power-normalized transforms, and we consider the Hilbert space $\mathbb{C}^D = \mathbb{C}^{D_\sigma}$ of Appendix S7 and the subspace $V = V_\sigma = \text{Span}(w_{n,\sigma}^{(1)}(t), \ldots, w_{n,\sigma}^{(K)}(t))$ then we make use of the model $v = v_{n,\sigma}(t)$ described in Appendix S7, i.e., $w_{n,\sigma}^{(0)}(t) = v_{n,\sigma}(t) + d_{n,\sigma}(t)$. Here, dependencies on σ, n and t are initially made explicit for clarity. By the reasoning of Appendix S7, we can write

$$v_{n,\sigma}(t) = \beta_1(\sigma) w_{n,\sigma}^{(1)}(t) + \cdots + \beta_K(\sigma) w_{n,\sigma}^{(K)}(t). \tag{25}$$

This is the right side of the best-fitting model (Eq.2) from the main text.

We let $h_{n,\sigma}(t) = \frac{v_{n,\sigma}(t)}{\|v_{n,\sigma}(t)\|}$ and $\Pi_\sigma^{(0h)} = \|v_{n,\sigma}(t)\|$, so that $v_{n,\sigma}(t) = \Pi_\sigma^{(0h)} h_{n,\sigma}(t)$ and $w_{n,\sigma}^{(0)}(t) = \Pi_\sigma^{(0h)} h_{n,\sigma}(t) + d_{n,\sigma}(t)$. Because $w_{n,\sigma}^{(0)}(t)$ is power normalized,

$$\begin{aligned}
1 &= \|w_{n,\sigma}^{(0)}(t)\| && (26)\\
&= \|v_{n,\sigma}(t) + d_{n,\sigma}(t)\| && (27)\\
&= \sqrt{\langle v_{n,\sigma}(t) + d_{n,\sigma}(t), v_{n,\sigma}(t) + d_{n,\sigma}(t)\rangle} && (28)\\
&= \sqrt{\langle v_{n,\sigma}(t), v_{n,\sigma}(t)\rangle + \langle d_{n,\sigma}(t), d_{n,\sigma}(t)\rangle} && (29)\\
&= \sqrt{\|v_{n,\sigma}(t)\|^2 + \|d_{n,\sigma}(t)\|^2}, && (30)
\end{aligned}$$

so $\Pi_\sigma^{(0h)}$, which is a nonnegative real number, is less than or equal to 1. The larger $\Pi_\sigma^{(0h)}$ is, the closer $h_{n,\sigma}(t)$ comes to $v_{n,\sigma}(t)$, and the closer $v_{n,\sigma}(t)$ comes to $w_{n,\sigma}^{(0)}(t)$. Because

$$\begin{aligned}
\langle w_{n,\sigma}^{(0)}(t), h_{n,\sigma}(t)\rangle &= \langle v_{n,\sigma}(t) + d_{n,\sigma}(t), \frac{v_{n,\sigma}(t)}{\|v_{n,\sigma}(t)\|}\rangle && (31)\\
&= \langle v_{n,\sigma}(t), \frac{v_{n,\sigma}(t)}{\|v_{n,\sigma}(t)\|}\rangle && (32)\\
&= \|v_{n,\sigma}(t)\| && (33)\\
&= \Pi_\sigma^{(0h)}, && (34)
\end{aligned}$$

$\Pi_\sigma^{(0h)}$ equals the spatial coherence of $w_{n,\sigma}^{(0)}(t)$ and $h_{n,\sigma}(t)$, which explains the choice of notation $\Pi_\sigma^{(0h)}$ (see Appendix S6). By orthogonality of $v_{n,\sigma}(t)$ and $d_{n,\sigma}(t)$ (i.e., $\langle v_{n,\sigma}(t),\, d_{n,\sigma}(t)\rangle = 0$), $\langle h_{n,\sigma}(t),\, d_{n,\sigma}(t)\rangle = 0$, as well.

Theorem 1. A Moran theorem for wavelet linear models *Using the notation defined above, and if* $r_\sigma^{(0)}(t) = \frac{1}{N}\sum_{n=1}^N w_{n,\sigma}^{(0)}(t)$ *is the wavelet mean field of the* $x_n^{(0)}(t)$, $r_\sigma^{(h)}(t) = \frac{1}{N}\sum_{n=1}^N h_{n,\sigma}(t)$ *and* $r_\sigma^{(d)}(t) = \frac{1}{N}\sum_{n=1}^N d_{n,\sigma}(t)$, *then the time-averaged squared magnitude of* $r_\sigma^{(0)}(t)$ *is*

$$\frac{1}{T}\sum_{t=1}^{T}|r_\sigma^{(0)}(t)|^2 = |\Pi_\sigma^{(0h)}|^2\frac{1}{T}\sum_{t=1}^{T}|r_\sigma^{(h)}(t)|^2 \tag{35}$$

$$+\frac{1}{TN^2}\sum_{t=1}^{T}\sum_{n=1}^{N}\sum_{m\neq n}2\,\mathrm{Re}(\Pi_\sigma^{(0h)}h_{n,\sigma}(t)\overline{d_{m,\sigma}(t)}) \tag{36}$$

$$+\frac{1}{T}\sum_{t=1}^{T}|r_\sigma^{(d)}(t)|^2. \tag{37}$$

If populations $x_n^{(0)}(t)$ *do not interact with populations* $x_m^{(0)}(t)$ *(for* $m \neq n$*) at neighboring sites, nor are they directly affected by other conditions (environmental or biotic) in neighboring sites, interpreted as the term in (36) being negligible (see justification in the proof below), then*

$$\frac{1}{T}\sum_{t=1}^{T}|r_\sigma^{(0)}(t)|^2 \approx |\Pi_\sigma^{(0h)}|^2\frac{1}{T}\sum_{t=1}^{T}|r_\sigma^{(h)}(t)|^2 + \frac{1}{T}\sum_{t=1}^{T}|r_\sigma^{(d)}(t)|^2. \tag{38}$$

In other words, synchrony of $x_n^{(0)}(t)$ *can be divided into a component attributable to the variables* $x_n^{(k)}(t)$, $k = 1, \ldots, K$, *and residual synchrony. It follows that*

$$\{|r_\sigma^{(0)}(t)|\}_t \geq |\Pi_\sigma^{(0h)}|^2\{|r_\sigma^{(h)}(t)|\}_t, \tag{39}$$

where $\{x\}_t$ *represents the time average of the square of* x. *If, in addition, the only synchronizing influence on* $x_n^{(0)}(t)$ *is the variables* $x_n^{(k)}(t)$, $k = 1, \ldots, K$, *interpreted as the second term on the right of (38) being negligible, then*

$$\{|r_\sigma^{(0)}(t)|\}_t \approx |\Pi_\sigma^{(0h)}|^2\{|r_\sigma^{(h)}(t)|\}_t. \tag{40}$$

Thus $|\Pi_\sigma^{(0h)}|^2\{|r_\sigma^{(h)}(t)|\}_t$ *gives the amount of synchrony explained solely by the synchronizing influence of the variables* $x_n^{(k)}(t)$, $k = 1, \ldots, K$.

Proof. We omit subscripts σ and time arguments t, sums over n and m are understood to be from 1 to N, and sums over t are understood to be from 1 to T.

$$|r^{(0)}|^2 = \left(\frac{1}{N}\sum_n w_n^{(0)}\right)\left(\frac{1}{N}\sum_m \overline{w_m^{(0)}}\right) \tag{41}$$

$$= \frac{1}{N^2}\left(\sum_n(\Pi^{(0h)}h_n + d_n)\right)\left(\sum_m(\overline{\Pi^{(0h)}}\,\overline{h_m} + \overline{d_m})\right) \tag{42}$$

$$= \frac{1}{N^2}\sum_n\sum_m(\Pi^{(0h)}h_n\overline{\Pi^{(0h)}}\,\overline{h_m} + d_n\overline{\Pi^{(0h)}}\,\overline{h_m} + \Pi^{(0h)}h_n\overline{d_m} + d_n\overline{d_m}) \tag{43}$$

$$= |\Pi^{(0h)}|^2\frac{1}{N^2}\sum_n\sum_m h_n\overline{h_m} + \frac{1}{N^2}\sum_n\sum_m(d_n\overline{\Pi^{(0h)}}\,\overline{h_m} + \Pi^{(0h)}h_n\overline{d_m}) + \frac{1}{N^2}\sum_n\sum_m d_n\overline{d_m} \tag{44}$$

$$= |\Pi^{(0h)}|^2\left|\frac{1}{N}\sum_n h_n\right|^2 + \frac{1}{N^2}\sum_n\sum_m 2\,\mathrm{Re}(\Pi^{(0h)}h_n\overline{d_m}) + \left|\frac{1}{N}\sum_n d_n\right|^2. \tag{45}$$

Taking the time average and using the fact that $\langle h_n, d_n \rangle = 0$ to exclude summands with $n = m$ in the middle term of (45), we get

$$\frac{1}{T}\sum_t |r^{(0)}|^2 = |\Pi^{(0h)}|^2 \frac{1}{T}\sum_t \left|\frac{1}{N}\sum_n h_n\right|^2 + \frac{1}{TN^2}\sum_t\sum_n\sum_{m\neq n} 2\,\mathrm{Re}(\Pi^{(0h)} h_n \overline{d_m}) + \frac{1}{T}\sum_t \left|\frac{1}{N}\sum_n d_n\right|^2, \quad (46)$$

which proves the first statement of the theorem, i.e., (35)-(37). The remaining claims follow straightforwardly.

The assumption of non-interacting populations not affected by the environments of neighboring sites was interpreted to mean (36) is negligible. This is reasonable because for such populations, the magnitude of $\frac{1}{TN^2}\sum_t\sum_n\sum_{m\neq n} h_n\overline{d_m}$ should be negligible, as it quantifies relationships between environmental fluctuations at site n, built into the model h_n, and residuals at other sites, d_m. We tested the assumption that the cross terms of (36) were negligible for our best short- and long-timescale models, finding that they were (Appendix S15). ■

Appendix S15 Predicted synchrony, fractions of synchrony explained, and cross terms

Using the reasoning behind theorem 1 of Appendix S14, and continuing the notation from that section, we can consider $|\Pi_\sigma^{(0h)}||r_\sigma^{(h)}(t)|$ (see (45)) as it varies with σ and t as a 'predicted synchrony' function, comparable with the observed value of $|r_\sigma^{(0)}(t)|$. See, e.g., Fig.2b, which shows synchrony predicted by our best long-timescale model on long timescales, and which shows synchrony predicted by our best short-timescale model on short timescales.

Using the wavelet Moran theorem, we can further quantify the proportion of synchrony explained by the model as

$$q(\sigma) = \frac{|\Pi_\sigma^{(0h)}|^2 \frac{1}{T}\sum_{t=1}^T |r_\sigma^{(h)}(t)|^2}{\frac{1}{T}\sum_{t=1}^T |r_\sigma^{(0)}(t)|^2}. \quad (47)$$

This quantity was averaged over long (> 4 years) or short (< 4 years) timescales, respectively, to provide the fractions of synchrony explained, and quoted in the main text, by our best long- and short-timescale models of PCI, respectively: 61.1% for the long-timescale model, which had predictors growing season temperature and *C. finmarchicus* abundance; and only 3.1% for the short-timescale model, which had predictors *C. finmarchicus*, decapod larvae abundance, and echinoderm larvae abundance (see Results in main text). Because the short-timescale model explained such a small fraction of observed synchrony in PCI, we did not base any conclusions about the nature of PCI synchrony on the model.

We tested the assumption that the cross terms of (36) were negligible for our best short- and long-timescale models of PCI as follows. The quantity

$$\frac{\frac{1}{TN^2}\sum_{t=1}^T\sum_{n=1}^N\sum_{m\neq n} 2\,\mathrm{Re}(\Pi_\sigma^{(0h)} h_{n,\sigma}(t)\overline{d_{m,\sigma}(t)})}{\frac{1}{T}\sum_{t=1}^T |r_\sigma^{(0)}(t)|^2} \quad (48)$$

was averaged over long timescales for our best long-timescale model of PCI. The result was 11%. The quantity was averaged over short time scales for our best short-timescale model of PCI. The result was 1.4%. The long-timescale value was relatively small compared to the the fraction of synchrony explained (see main text and above). As stated above, the fraction of synchrony explained by the short-timescale model was very small so the model was not considered informative about synchrony. The 11% contribution of cross terms in the long-timescale model indicates a tendency for the model at a location to be slightly positively associated with model residuals at other locations; these associations may be due to mixing effects, which render PCI values in each area of the sea slightly dependent on the driving effects of temperature and *C. finmarchicus* fluctuations in nearby parts of the sea.

The quantity q is also, as we now show, the fraction of the total power in the mean signal $\frac{1}{N}\sum_n x_n^{(0)}(t)$ explained by the synchrony of the $x_n^{(k)}(t)$. Consider the power P of the mean of the un-normalized wavelet transforms $W_{n,\sigma}(t)$, which is also the power of the wavelet transform of the mean $\frac{1}{N}\sum_n x_n^{(0)}(t)$, by linearity of the transform. This represents the amount of variability in the mean signal at scale σ:

$$P = \frac{1}{T}\sum_{t=1}^{T}\left(\frac{1}{N}\sum_{n=1}^{N} W_{n,\sigma}(t)\right)\overline{\left(\frac{1}{N}\sum_{n=1}^{N} W_{n,\sigma}(t)\right)} \tag{49}$$

$$= \left[\frac{1}{NT}\sum_{n=1}^{N}\sum_{t=1}^{T} W_{n,\sigma}(t)\overline{W_{n,\sigma}(t)}\right]\left[\frac{1}{T}\sum_{t=1}^{T}\left(\frac{1}{N}\sum_{n=1}^{N} w_{n,\sigma}(t)\right)\overline{\left(\frac{1}{N}\sum_{n=1}^{N} w_{n,\sigma}(t)\right)}\right] \tag{50}$$

$$= \left[\frac{1}{N}\sum_{n=1}^{N}\frac{1}{T}\sum_{t=1}^{T} W_{n,\sigma}(t)\overline{W_{n,\sigma}(t)}\right]\left[\{|r_\sigma^{(0)}(t)|\}_t\right]. \tag{51}$$

Here we use the definitions of the power normalized transform and the mean field. Thus the power of the mean signal depends on the mean of wavelet powers and on synchrony $\{|r_\sigma^{(0)}(t)|\}_t$. The component of synchrony attributable to the model v is $|\Pi_\sigma^{(0h)}|^2\{|r_\sigma^{(h)}(t)|\}_t$, so the component of P explained by v is

$$P_v = \left[\frac{1}{NT}\sum_{n=1}^{N}\sum_{t=1}^{T} W_{n,\sigma}(t)\overline{W_{n,\sigma}(t)}\right]\left[|\Pi_\sigma^{(0h)}|^2\{|r_\sigma^{(h)}(t)|\}_t\right] \tag{52}$$

and the fraction of the total power of the mean explained by synchrony in the $x_n^{(k)}(t)$ is

$$\frac{P_v}{P} = \frac{|\Pi_\sigma^{(0h)}|^2\frac{1}{T}\sum_{t=1}^{T}|r_\sigma^{(h)}(t)|^2}{\frac{1}{T}\sum_{t=1}^{T}|r_\sigma^{(0)}(t)|^2} = q(\sigma). \tag{53}$$

If the number of sampling locations is large, a case for which local effects are negligible, the remaining power in the fluctuations of the total population may be explained by the synchronizing influence of one or more other variables unrelated to the $x_n^{(k)}(t)$ for $k = 1, \ldots, K$.

Appendix S16 Testing for significance of interaction effects

We tested whether the synchrony attributable to our model included a substantial effect due to interactions between the influences of the predictor variables occasioned by their non-independence. We here describe the case in which the best model had two predictors, $K = 2$ in Eq.2 of the main text, for simplicity and because the best long-timescale model on which we focus in Results had $K = 2$. Having fitted the model with data to determine $\beta_k(\sigma)$ for $k = 1, 2$, we replaced $x_n^{(2)}(t)$ by *spatially synchronous surrogates* (Appendix S8), $\tilde{x}_n^{(2)}(t)$, which had the same patterns of autocorrelation and synchrony as the unmanipulated $x_n^{(2)}(t)$, but were unrelated to $x_n^{(1)}(t)$. Mean squared model synchrony $\{|\tilde{r}_\sigma^{(h)}(t)|\}_t$ was recomputed using the surrogates and their transforms $\tilde{w}_{n,\sigma}^{(2)}(t)$ in Eq.2 of the main text in place of $w_{n,\sigma}^{(2)}(t)$ (but still using the original $\beta_k(\sigma)$). This was done for 1000 independent sets of spatially synchronous surrogates and the average surrogate mean squared model synchrony was computed and compared to $\{|r_\sigma^{(h)}(t)|\}_t$. For comparison, the same procedure was carried out with *asynchronous surrogates* (Appendix S8) of $x_n^{(2)}(t)$, which randomize away both relationships with $x_n^{(1)}(t)$ and synchrony of $x_n^{(2)}(t)$, while preserving the autocorrelation structure of $x_n^{(2)}(t)$.

Appendix S17 Attributing synchrony to individual predictors and interactions among predictors

The interaction terms in the following theorem indicate synchrony is not only the 'sum of its parts'.

Theorem 2. Synchrony attribution theorem *Using the notation of the previous two sections, the portion of synchrony explained by a wavelet model can be partitioned as*

$$|\Pi_\sigma^{(0h)}|^2 \frac{1}{T}\sum_t |r_\sigma^{(h)}(t)|^2 =$$

$$\sum_k |\beta_k(\sigma)|^2 \frac{1}{T}\sum_t \left|\frac{1}{N}\sum_n w_{n,\sigma}^{(k)}(t)\right|^2 + \sum_j \sum_{k\neq j} \frac{1}{T}\sum_t \left(\beta_j(\sigma)\frac{1}{N}\sum_m w_{m,\sigma}^{(j)}(t)\right)\overline{\left(\beta_k(\sigma)\frac{1}{N}\sum_n w_{n,\sigma}^{(k)}(t)\right)}. \quad (54)$$

The kth summand in the first term on the right is the timescale-specific portion of synchrony attributable to the kth predictor. The second term on the right is the timescale-specific portion of synchrony attributable to interaction effects. The second term is real-valued and can be positive or negative.

Proof. The following equations omit explicit dependencies on σ and t for brevity.

$$|\Pi^{(0h)}|^2 \frac{1}{T}\sum_t |r^{(h)}|^2 = \frac{1}{T}\sum_t \left|\Pi^{(0h)}\frac{1}{N}\sum_n h_n\right|^2 \quad (55)$$

$$= \frac{1}{T}\sum_t \left|\frac{1}{N}\sum_n v_n\right|^2 \quad (56)$$

$$= \frac{1}{T}\sum_t \left|\frac{1}{N}\sum_n\sum_k \beta_k w_n^{(k)}\right|^2 \quad (57)$$

$$= \frac{1}{TN^2}\sum_t \left[\left(\sum_j\sum_m \beta_j w_m^{(j)}\right)\overline{\left(\sum_k\sum_n \beta_k w_n^{(k)}\right)}\right] \quad (58)$$

$$= \frac{1}{TN^2}\sum_t \left[\sum_k \left|\sum_n \beta_k w_n^{(k)}\right|^2 + \sum_j\sum_{k\neq j}\left(\sum_m \beta_j w_m^{(j)}\right)\overline{\left(\sum_n \beta_k w_n^{(k)}\right)}\right] \quad (59)$$

$$= \frac{1}{T}\sum_t\sum_k \left|\frac{1}{N}\sum_n \beta_k w_n^{(k)}\right|^2 + \frac{1}{T}\sum_t\sum_j\sum_{k\neq j}\left(\frac{1}{N}\sum_m \beta_j w_m^{(j)}\right)\overline{\left(\frac{1}{N}\sum_n \beta_k w_n^{(k)}\right)} \quad (60)$$

$$= \sum_k |\beta_k|^2 \frac{1}{T}\sum_t \left|\frac{1}{N}\sum_n w_n^{(k)}\right|^2 + \sum_j\sum_{k\neq j}\frac{1}{T}\sum_t \left(\beta_j\frac{1}{N}\sum_m w_m^{(j)}\right)\overline{\left(\beta_k\frac{1}{N}\sum_n w_n^{(k)}\right)}. \quad (61)$$

The remaining statements of the theorem follow straightforwardly. ∎

Applying the synchrony attribution theorem, we can partition the fraction of synchrony explained (47) as $q(\sigma) = \sum_k q_k(\sigma) + q_{\text{int}}(\sigma)$, where

$$q_k(\sigma) = \frac{|\beta_k|^2 \frac{1}{T}\sum_t \left|\frac{1}{N}\sum_n w_n^{(k)}\right|^2}{\frac{1}{T}\sum_{t=1}^T |r_\sigma^{(0)}(t)|^2} \quad (62)$$

$$q_{\text{int}}(\sigma) = \frac{\sum_j\sum_{k\neq j}\frac{1}{T}\sum_t \left(\beta_j\frac{1}{N}\sum_m w_m^{(j)}\right)\overline{\left(\beta_k\frac{1}{N}\sum_n w_n^{(k)}\right)}}{\frac{1}{T}\sum_{t=1}^T |r_\sigma^{(0)}(t)|^2}. \quad (63)$$

These quantities were averaged over long timescales to provide the fractions quoted in the main text of long-timescale synchrony in PCI explained by growing season temperature (41.4%), *C. finmarchicus* abundance (5.4%), and interactions between these two (14.3%). The sum of the quantities, 61.1%, is the previously-quoted fraction of long-timescale synchrony explained by our best long-timescale model, as expected.

Attribution of synchrony to particular drivers and interaction effects as well as the construction of best models on which this attribution is based are both complicated by the regime shift that occurred in the 1980s in the seas around the UK (Beaugrand and Reid, 2003; PCI increased, *C. finmarchicus* abundance decreased, and there were other effects). The regime shift may mean there were changes in biological dependencies from before to after the shift, so that our results represent an average of distinct phenomena before and after the regime shift instead of being a reflection of one consistent set of relationships between variables. This possibility is difficult to assess because time series are too short to reliably perform wavelet analyses separately for data before and after the shift.

Appendix S18 Tests of methods using the numeric example

Treating the simulated time series of the numeric example ($\alpha(n,t)$ and $\beta(n,t)$ from Fig.1a,d and $\gamma^{(3)}(n,t)$) in the same way as real ecological data, we evaluated our wavelet methods by checking they gave results consistent with the construction of those data.

We constructed and tested a wavelet model of $\gamma^{(3)}(n,t)$ as described in Appendix S9. Working back from the time series only, we constructed the best fit model of the wavelet transform of $\gamma^{(3)}(n,t)$ in terms of the wavelet transforms of its two drivers $\alpha(n,t)$ and $\beta(n,t)$, as in equation 2. We applied the method of spatially synchronous surrogates (Appendix S8) to determine that adding the second variable to a one-variable model incorporating only either $\alpha(n,t)$ or $\beta(n,t)$ would produce a statistically significant improvement ($p = 0.000$ for 1000 surrogates in both cases) in goodness of fit.

We verified that the synchrony explicable by the best fit model (according to the wavelet Moran theorem, see Appendix S14 and Appendix S15) is consistent with the actual synchrony of $\gamma(3)(n,t)$, as expected since the local noise added to $\gamma^{(3)}$ was asynchronous. At the 20 year timescale, the expected synchrony according to the best fit model was 1.022 times the synchrony observed in the wavelet mean field of $\gamma(n,t)$, i.e., all of the synchrony in $\gamma^{(3)}$ was attributable to $\alpha(n,t)$ and $\beta(n,t)$, as expected. In the analysis of real PCI data, the synchrony associated with the statistical model we constructed was only some fraction of the observed synchrony, due to the other, unattributed components of variability having non-zero spatial synchrony of their own.

We evaluated the size of the interaction effects between $\alpha(n,t)$ and $\beta(n,t)$ as in Appendix S16: 1) we evaluated the mean squared synchrony of the model (evaluated at the 20-year timescale); and we compared it to 2) the same quantity calculated after replacing $\alpha(n,t)$ by synchrony-preserving surrogates (we used 1000 surrogates and took the average). The former quantity was 1.24 times the latter, showing substantial interaction effects were detected.

Appendix S19 Lemmas and extended computations

Lemma 3. $\mathrm{cov}_t(\sin(wt), \sin(wt - \varphi\pi)) \approx \frac{1}{2}\cos(\varphi\pi)$*, where* $w = 2\pi/20$*,* cov_t *is the sample covariance computed over* $t = 1, \ldots, T-1$*, and* T *is divisible by* 20*.*

Proof.

$$\text{cov}_t(\sin(wt), \sin(wt - \varphi\pi)) = \frac{1}{T-1}\sum_{t=0}^{T-1} \sin(wt)\sin(wt-\varphi\pi) \tag{64}$$

$$= \frac{1}{T-1}\sum_{t=0}^{T-1} \sin(wt)[\sin(wt)\cos(\varphi\pi) - \cos(wt)\sin(\varphi\pi)] \tag{65}$$

$$= \cos(\varphi\pi)\frac{1}{T-1}\sum_{t=0}^{T-1} \sin^2(wt) - \sin(\varphi\pi)\frac{1}{T-1}\sum_{t=0}^{T-1}\sin(wt)\cos(wt) \tag{66}$$

$$\approx \frac{1}{2}\cos(\varphi\pi) - \sin(\varphi\pi)\frac{1}{T-1}\sum_{t=0}^{T-1}\frac{\sin(2wt)}{2} \tag{67}$$

$$= \frac{1}{2}\cos(\varphi\pi), \tag{68}$$

where (65) and (67) follow from the trigonometric identities $\sin(x \pm y) = \sin(x)\cos(y) \pm \cos(x)\sin(y)$ and $\sin(2x) = 2\sin(x)\cos(x)$, respectively. ∎

The following lemma was taken, verbatim, from Sheppard et al. [2016].

Lemma 4. *The spatial coherence, $|\Pi_\sigma^{(01)}|$, is between* 0 *and* 1.

Proof. See lemma 2 of Sheppard et al. [2016]. ∎

Lemma 5. *Let X be a complex Hilbert space, let $\langle \cdot, \cdot \rangle$ be its inner product, let V be a finite-dimensional subspace of X, and take $x \in X$. Then there exists a unique $v \in V$ such that $x = v + d$ and $d \perp V$. Furthermore, $\|d\| \leq \|x - w\|$ for all $w \in V$, with equality if and only if $w = v$. And $\frac{|\langle x,w\rangle|}{\|x\|\|w\|} \leq \frac{|\langle x,v\rangle|}{\|x\|\|v\|}$ for all $w \in V$, with equality if and only if $w = \alpha v$ for some complex number α. Additionally, if $v_1, \ldots, v_K \in X$, $V = \text{Span}(v_1, \ldots, v_K)$, and $v = \beta_1 v_1 + \cdots + \beta_K v_K$, then $\beta = (\beta_1, \ldots, \beta_K)^t$ is a solution of the equation $M\beta = p$ where $M = \left[\frac{\langle v_j, v_i\rangle}{\|v_i\|^2}\right]_{ij}$ and $p = (p_1, \ldots, p_K)^t$ with $p_j = \frac{\langle x, v_j\rangle}{\|v_j\|^2}$.*

Proof. By theorem 2.1 on page 253 in Loomis and Sternberg [1968], $X = V \oplus V^\perp$, so that gives a unique $v \in V$ and $d \in V^\perp$ such that $x = v + d$. By lemma 2.1 on page 252 of Loomis and Sternberg [1968], $\|d\| \leq \|x - w\|$ for all $w \in V$, with equality if and only if $w = v$.

Now let $w_1, \ldots, w_m$ be an orthonormal basis for V such that $w_1 = \frac{v}{\|v\|}$. Then, given $w \in V$, write $w = \alpha_1 w_1 + \cdots + \alpha_m w_m$. We have

$$\frac{|\langle x, w\rangle|}{\|x\|\|w\|} = \frac{|\langle \|v\|w_1 + d, \alpha_1 w_1 + \cdots + \alpha_m w_m\rangle|}{\|x\|\sqrt{|\alpha_1|^2 + \cdots + |\alpha_m|^2}} = \frac{\|v\||\alpha_1|}{\|x\|\sqrt{|\alpha_1|^2 + \cdots + |\alpha_m|^2}} \leq \frac{\|v\|}{\|x\|} = \frac{|\langle x, v\rangle|}{\|x\|\|v\|}. \tag{69}$$

Equality holds if and only if $\alpha_2 = \cdots = \alpha_m = 0$, in other words, if and only if $w = \alpha v$.

The last statement of the lemma is equivalent to $\sum_{j=1}^K \frac{\langle v_j, v_i\rangle}{\|v_i\|^2}\beta_j = \frac{\langle x, v_i\rangle}{\|v_i\|^2}$ for all $i = 1, \ldots, K$, but

$$\frac{\langle x, v_i\rangle}{\|v_i\|^2} = \frac{\langle v, v_i\rangle}{\|v_i\|^2} = \frac{\langle \sum_{j=1}^K \beta_j v_j, v_i\rangle}{\|v_i\|^2} = \sum_{j=1}^K \beta_j \frac{\langle v_j, v_i\rangle}{\|v_i\|^2}. \tag{70}$$

∎

The following lemma was taken, verbatim, from Sheppard et al. [2016].

Lemma 6. *The mean squared magnitude of the wavelet mean field,* $\frac{1}{T}\sum_{t=1}^{T}|r_\sigma(t)|^2$*, satisfies*

$$\frac{1}{T}\sum_{t=1}^{T}|r_\sigma(t)|^2 = \frac{\frac{1}{T}\sum_{t=1}^{T}\left[\left(\frac{1}{N}\sum_{n=1}^{N}W_{n,\sigma}(t)\right)\left(\frac{1}{N}\sum_{n=1}^{N}\overline{W_{n,\sigma}(t)}\right)\right]}{\frac{1}{N}\sum_{n=1}^{N}\frac{1}{T}\sum_{t=1}^{T}W_{n,\sigma}(t)\overline{W_{n,\sigma}(t)}} \tag{71}$$

and

$$0 \leq \frac{1}{T}\sum_{t=1}^{T}|r_\sigma(t)|^2 \leq 1, \tag{72}$$

with

$$\frac{1}{T}\sum_{t=1}^{T}|r_\sigma(t)|^2 = 1 \tag{73}$$

if and only if all the time series are identical. The denominator of the right side of (71) is the average of the powers of all the time series, $x_n(t)$*, and the numerator is the power of the average time series, and hence equation (71) is a timescale-specific generalization of*

$$\frac{\mathrm{var}\left(\frac{1}{N}\sum_{n=1}^{N}x_n(t)\right)}{\left(\frac{1}{N}\sum_{n=1}^{N}\mathrm{var}(x_n(t))\right)}. \tag{74}$$

Proof. See lemma 1 of Sheppard et al. [2016]. ■

References

P. S. Addison. *The Illustrated Wavelet Transform Handbook: Introductory Theory and Applications in Science, Engineering, Medicine and Finance.* Taylor and Francis, New York, 2002.

A. Bandrivskyy, A. Bernjak, P. V. E. McClintock, and A. Stefanovska. Wavelet phase coherence analysis: Application to skin temperature and blood flow. *Cardiovasc. Eng.*, 4(1):89–93, 2004.

G. Beaugrand and P.C. Reid. Long-term changes in phytoplankton, zooplankton and salmon related to climate. *Global Change Biology*, 9:801–817, 2003.

G. Beaugrand, K.M. Brander, J.A. Lindley, S. Souissi, and P.C. Reid. Plankton effect on cod recruitment in the North Sea. *Nature*, 426:661–664, 2003.

J. Bell, E.C. Burkness, A.E. Milne, D.W. Onstad, M. Abrahamson, K.L. Hamilton, and W.D. Hutchinson. Putting the brakes on a cycle: bottom-up affects damp cycle amplitude. *Ecology Letters*, 15:310–318, 2012.

B. Cazelles, M. Chavez, D. Berteaux, F. Ménard, J.O. Vik, S. Jenouvrier, and N.C. Stenseth. Wavelet analysis of ecological timeseries. *Oecologia*, 156:287–304., 2008.

B. Cazelles, K. Cazelles, and M. Chavez. Wavelet analysis in ecology and epidemiology: imapct of statistical tests. *Journal of the Royal Society Interface*, 11:20130585, 2014.

J.M. Colebrook and G. A. Robinson. Continuous plankton records: Seasonal cycles of phytoplankton and copepods in the north-eastern Atlantic and the North Sea. *Bulletins of Marine Ecology*, 6:123–139, 1965.

Emma J. Defriez and Daniel C. Reuman. A global geography of synchrony for marine phytoplankton. *Global Ecology and Biogeography*, 26(8):867–877, 2017a. ISSN 1466-8238. doi: 10.1111/geb.12594. URL `http://dx.doi.org/10.1111/geb.12594`.

Emma J. Defriez and Daniel C. Reuman. A global geography of synchrony for terrestrial vegetation. *Global Ecology and Biogeography*, 26(8):878–888, 2017b. ISSN 1466-8238. doi: 10.1111/geb.12595. URL `http://dx.doi.org/10.1111/geb.12595`.

B.T. Grenfell, O.N. Bjørnstad, and J. Kappey. Traveling waves and spatial hierarchies in measles epidemics. *Nature*, 414:716–723, 2001.

T.H. Keitt. Coherent ecological dynamics induced by large-scale disturbance. *Nature*, 454:331–335, 2008.

T.H. Keitt and J. Fischer. Detection of scale-specific community dynamics using wavelets. *Ecology*, 87: 2895–2904, 2006.

L.H. Loomis and S. Sternberg. *Advanced Calculus.* Addison-Wesley Series in Mathematics, New York, 1968.

S.D. Meyers, B.G. Kelly, and J.J. O'Brien. An introduction to wavelet analysis in oceanography and meteorology: with application to the dispersion of yanai waves. *Mon. Weather Rev.*, 121:2858–2866, 1993.

P.A.P. Moran. The statistical analysis of the Canadian Lynx cycle. *Australian Journal of Zoology*, 1: 291–298, 1953.

D. Prichard and J. Theiler. Generating surrogate data for time series with several simultaneously measured variables. *Phys. Rev. Lett.*, 73(7):951–954, 1994.

D. E. Raitsos, P. C. Reid, S. J. Lavender, M. Edwards, and A.J. Richardson. Extending the SeaWiFS chlorophyll data set back 50 years in the northeast Atlantic. *Geophysical Research Letters*, 32:L06603, 2005.

D. E. Raitsos, A. Walne, S. J. Lavendar, P. Licandro, P. C. Reid, and M. Edwards. A 60-year ocean colour data set from the continuous plankton recorder. *Journal of Plankton Research*, 35:158–164, 2013.

T. Rouyer, J.M. Fromentin, N.C. Stenseth, and B. Cazelles. Analysing multiple time series and extending significance testing in wavelet analysis. *Marine Ecology Progress Series*, 359:11–23, 2008.

R.M. Sakia. The Box-Cox transformation technique: a review. *The Statistician*, 41:169–178, 1992.

T. Schreiber and A. Schmitz. Surrogate time series. *Physica D*, 142(3-4):346–382, AUG 15 2000. ISSN 0167-2789.

L. W. Sheppard, A. C. Hale, S. Petkoski, P. V. E. McClintock, and A. Stefanovska. Characterizing an ensemble of interacting oscillators: The mean-field variability index. *Phys.Rev.E*, 87:012905, 2013.

L.W. Sheppard, J.R. Bell, R. Harrington, and D.C. Reuman. Changes in large-scale climate alter spatial synchrony of aphid pests. *Nature Climate Change*, 6:610–613, 2016. doi: 10.1038/nclimate2881.

L.W. Sheppard, P.C. Reid, and D.C. Reuman. Rapid surrogate testing of wavelet coherences. *European Physical Journal Nonlinear Biomedical Physics*, 5:1–9, 2017.

A. Stefanovska. Coupled oscillators - Complex but not complicated cardiovascular and brain interactions. *IEEE Engineering in Biology and Medicine Magazine*, 26(6):25–29, 2007. ISSN 0739-5175. doi: {10.1109/MEMB.2007.907088}.

J. W. Strutt. *Scientific Papers by John William Strutt, Baron Rayleigh, Vol. 3, pp. 47-187 (see in particular p. 52).* Cambridge University Press, Cambridge, 1902.

J. Theiler, S. Eubank, A. Longtin, B Galdrikian, and J.D. Farmer. Testing for nonlinearity in time series: the method of surrogate data. *Physica D*, 58(1–4):77–94, 1992.

C. Torrence and G. P. Compo. A practical guide to wavelet analysis. *Bull. Am. Meteorol. Soc.*, 79(1): 61–78, 1998.

C. Viboud, O.N. Bjørnstad, D.L. Smith, L. Simonsen, M.A. Miller, and B.T. Grenfell. Synchrony, waves, and spatial hierarchies in the spread of influenza. *Science*, 312:447–451, 2006.
