# Supplementary material for: Synchrony is more than its top-down and climatic parts: interacting Moran effects on phytoplankton in British seas
Source: PLoS Comput Biol. 2019 Mar 28;15(3):e1006744. doi: 10.1371/journal.pcbi.1006744 (PMC6438443; doi:10.1371/journal.pcbi.1006744)
Supplement: S1 Table — Of all models we considered (Methods), the model with the highest leave-one-out goodness of fit score for which no variables could be dropped without significantly reducing model fit was the model listed in the top row and analyzed in the main text. The table includes all models considered for which the leave-one-out goodness of fit score was at least 90% that of the top model and for which no variables could be dropped without significantly reducing model fit. (PDF) [file pcbi.1006744.s002.pdf]

Synchrony is more than its top-down and climatic parts: interacting Moran effects on phytoplankton in British seas: Supporting information

L. W. Sheppard, E. J. Defriez, P. C. Reid, D. C. Reuman

**Low frequency model**	**Leave-one-out score**
Growing season temperature,<br>*C. finmarchicus*	0.2045
Yearly temperature,<br>Summer salinity,<br>Autumn cloud cover,<br>*C. finmarchicus*	0.1991
Yearly temperature,<br>*C. finmarchicus*	0.1982
Growing season temperature,<br>Summer salinity,<br>*C. finmarchicus*	0.1918
Yearly temperature,<br>Summer salinity,<br>*C. finmarchicus*	0.1878

Table S1: Table of best long-timescale models. Of all models we considered (Methods), the model with the highest leave-one-out goodness of fit score for which no variables could be dropped without significantly reducing model fit was the model listed in the top row and analyzed in the main text. The table includes all models considered for which the leave-one-out goodness of fit score was at least 90% that of the top model and for which no variables could be dropped without significantly reducing model fit.
