# Supplementary material for: Synchrony is more than its top-down and climatic parts: interacting Moran effects on phytoplankton in British seas
Source: PLoS Comput Biol. 2019 Mar 28;15(3):e1006744. doi: 10.1371/journal.pcbi.1006744 (PMC6438443; doi:10.1371/journal.pcbi.1006744)
Supplement: S2 Table — (PDF) [file pcbi.1006744.s003.pdf]

Synchrony is more than its top-down and climatic parts: interacting Moran effects on phytoplankton in British seas: Supporting information

L. W. Sheppard, E. J. Defriez, P. C. Reid, D. C. Reuman

**Variable**	**Box-Cox coefficient**
Calanus I-IV	-0.116
Para-Pseudocalanus spp.	-0.036
Acartia spp. (unidentified)	0.102
Oithona spp.	0.193
Pseudocalanus elongatus adult	0.135
Temora longicornis	0.137
Centropages typicus	0.197
Calanus finmarchicus	0.088
Calanus helgolandicus	0.110
Metridia lucens	0.125
Echinoderm larvae	0.042
Decapoda larvae (total)	0.061
Euphausiacea (total)	0.099
Yearly temperature	0.942
Spring temperature	1.235
Summer temperature	0.583
Autumn temperature	0.241
Growing season temperature	0.972
Yearly wind speed	1.201
Spring wind speed	0.811
Summer wind speed	0.602
Autumn wind speed	0.951
Growing season wind speed	0.972
Yearly salinity	1.443
Spring salinity	1.616
Summer salinity	0.713
Autumn salinity	1.904
Growing season salinity	1.257
Yearly cloud cover	1.633
Spring cloud cover	1.482
Summer cloud cover	1.755
Autumn cloud cover	1.379
Growing season cloud cover	1.257

Table S2: The mean of the Box-Cox coefficients found at each location, for every variable.
