# Supplementary figures and images for: Synchrony is more than its top-down and climatic parts: interacting Moran effects on phytoplankton in British seas
Source: PLoS Comput Biol. 2019 Mar 28;15(3):e1006744. doi: 10.1371/journal.pcbi.1006744 (PMC6438443; doi:10.1371/journal.pcbi.1006744)

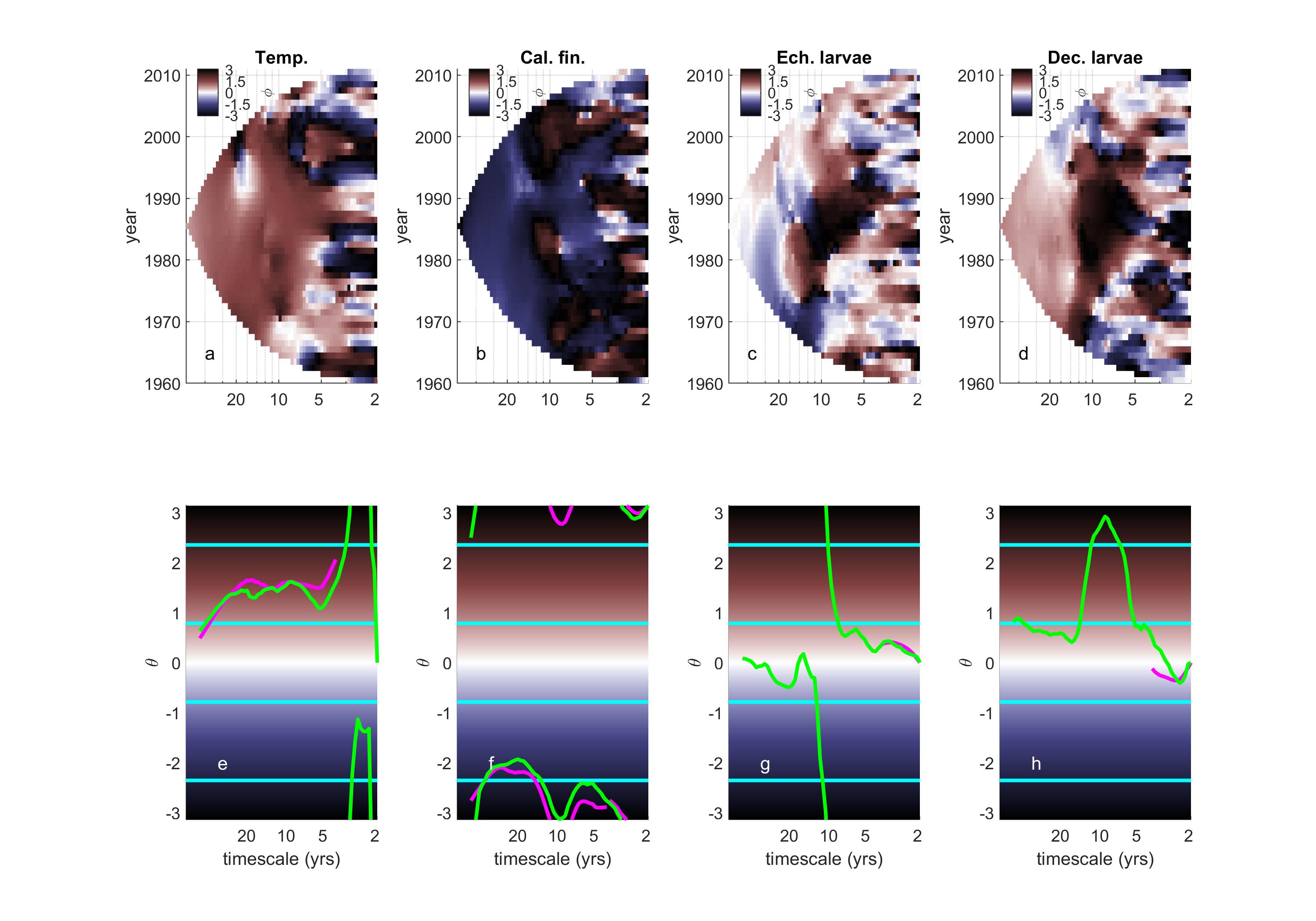

Supplement: S1 Fig — Top panels: The typical phase difference, φ, i.e. the phase of 1N∑neiη(n,t), where η(n, t) is the phase of wn,σ(0)(t)wn,σ(k)(t)¯, for the four predictors k appearing in the top-ranked models at long and short timescales: growing season temperature (a); C. finmarchicus abundance (b); echinoderm larvae abundance (c); and decapod larvae abundance (d). Bottom panels: The typical phase difference, θ, i.e. the phase of 1NT∑n,teiη(n,t) (green), compared to phases of corresponding coefficients βk(σ) from top-ranked models (magenta). Magenta lines extend across long timescales for predictors included in the top-ranked long-timescale model, across short timescales for predictors included in the top-ranked short-timescale model, and across long and short timescales for C. finmarchicus, since that variable was in the top-ranked models for both long and short timescales. Temp. = growing season temperature; C. fin. = C. finmarchicus; Ech. = echinoderm; Dec. = decapod. (JPG) [file pcbi.1006744.s004.jpg]

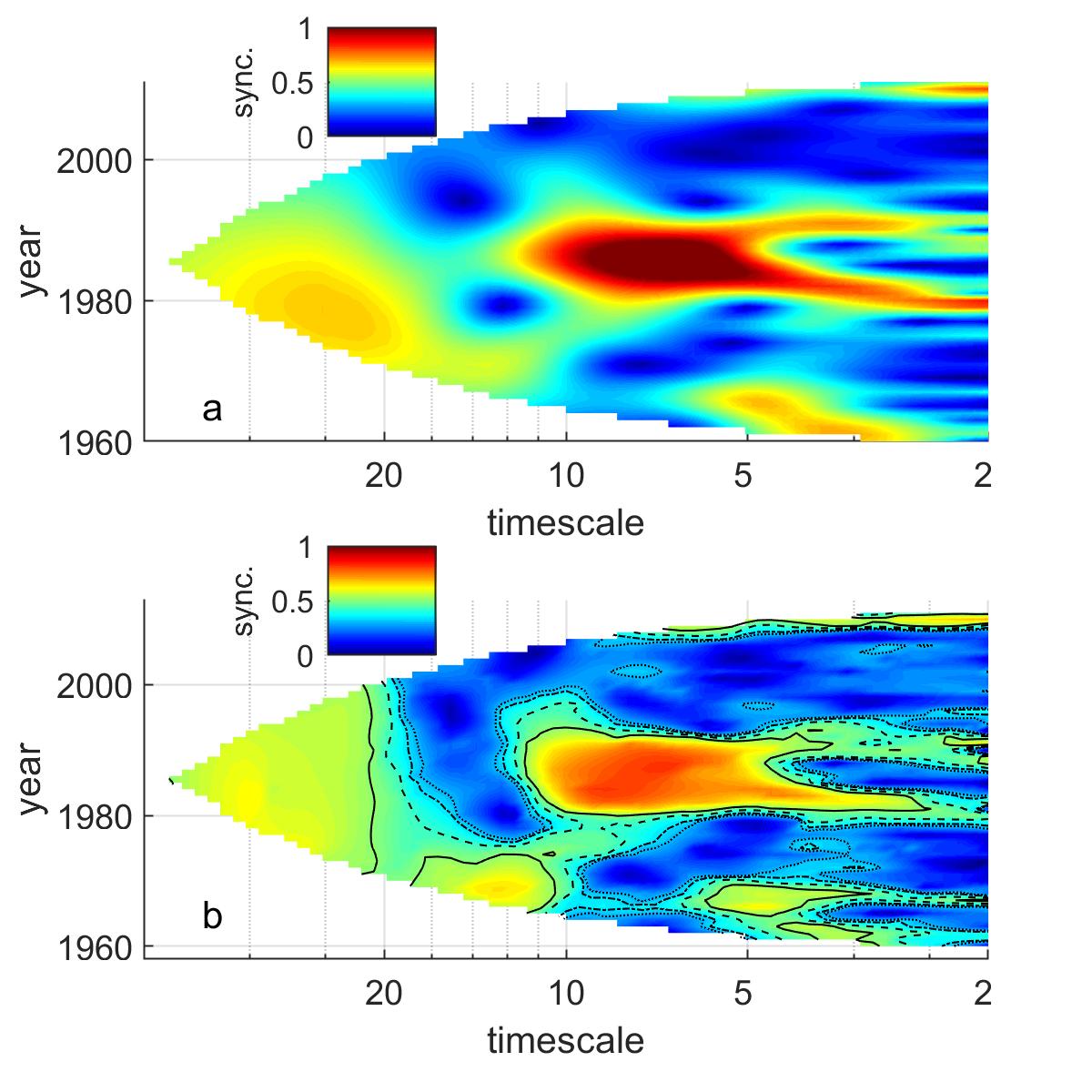

Supplement: S2 Fig — Statistical significance thresholds on the WPMFM are plotted as contours showing actual phase agreement between locations greater than the 90th, 95th, 99th and 99.9th percentile of a distribution of unsynchronized unit phasors (dotted, dash-dotted, dashed and line contours respectively). (JPG) [file pcbi.1006744.s005.jpg]

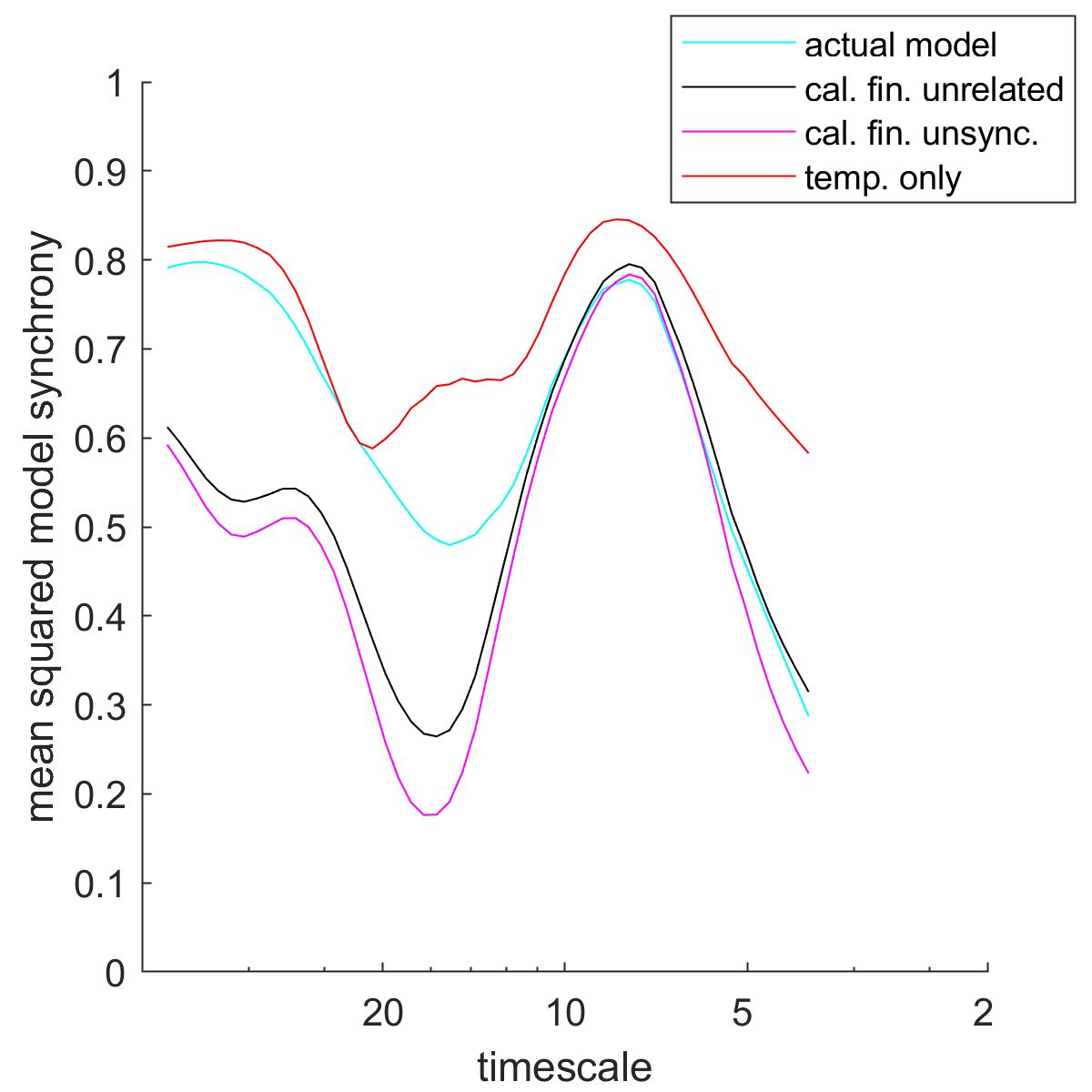

Supplement: S3 Fig — (JPG) [file pcbi.1006744.s006.jpg]

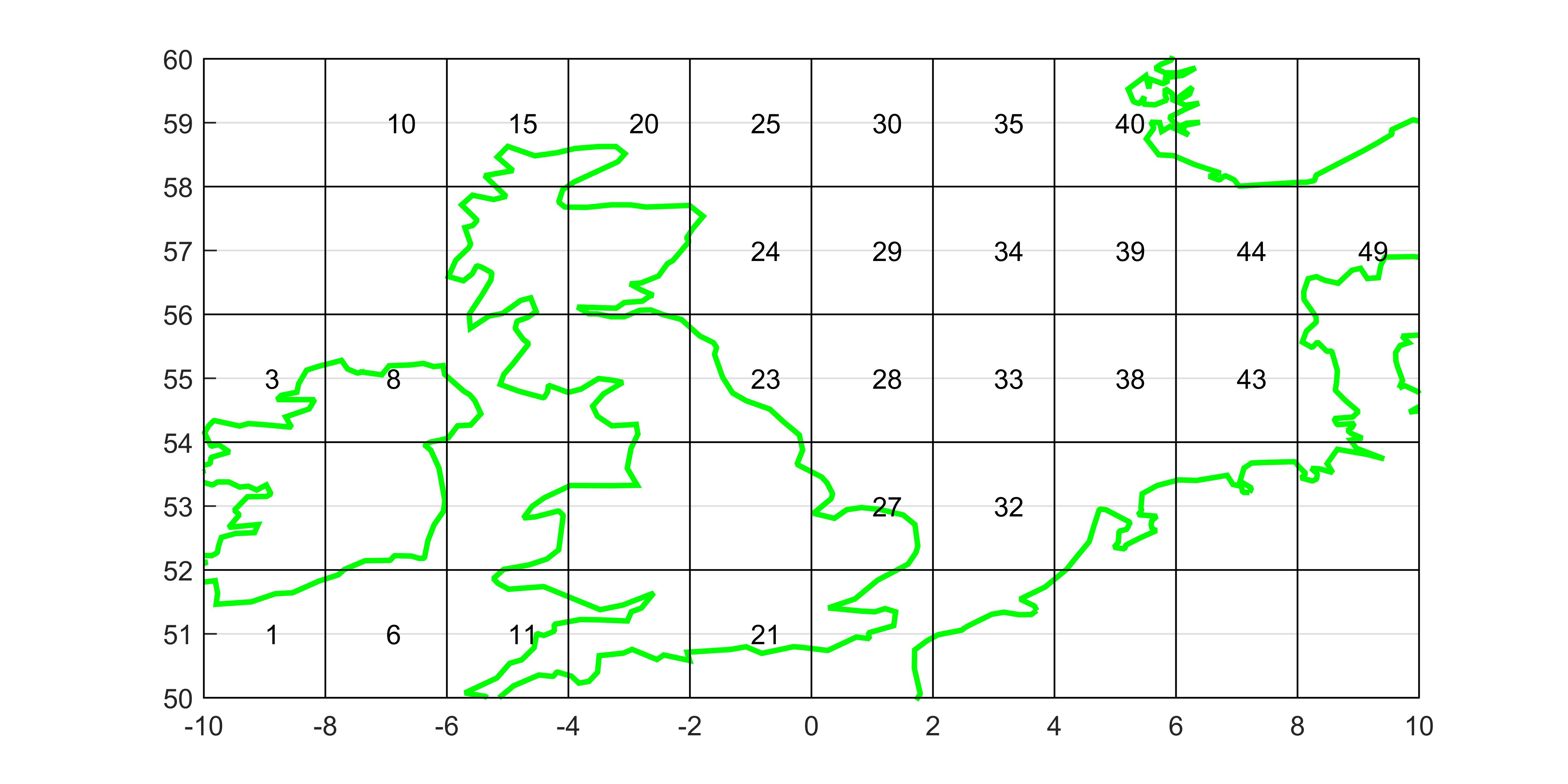

Supplement: S4 Fig — (JPG) [file pcbi.1006744.s007.jpg]

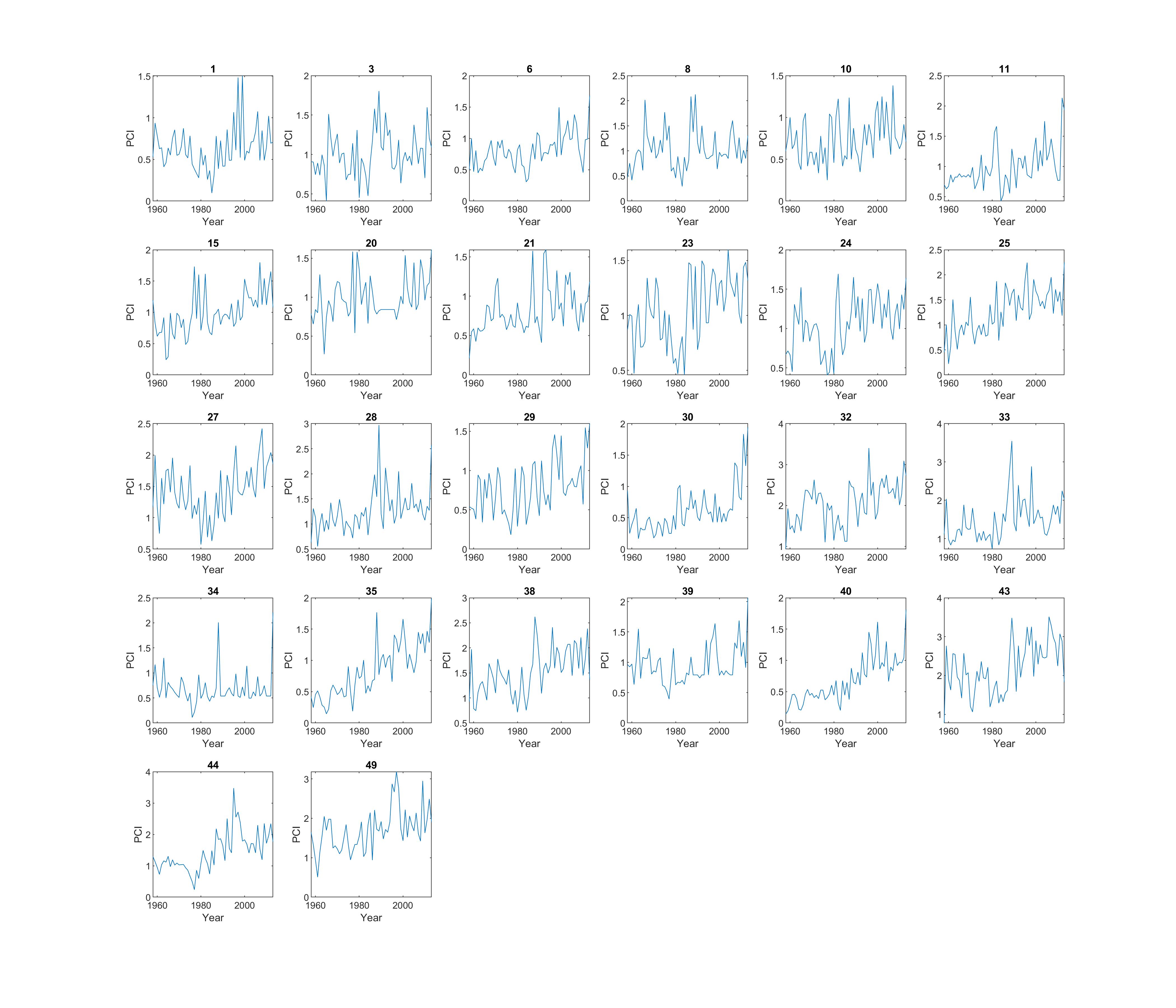

Supplement: S5 Fig — Plots numbered as in S4 Fig. (JPG) [file pcbi.1006744.s008.jpg]

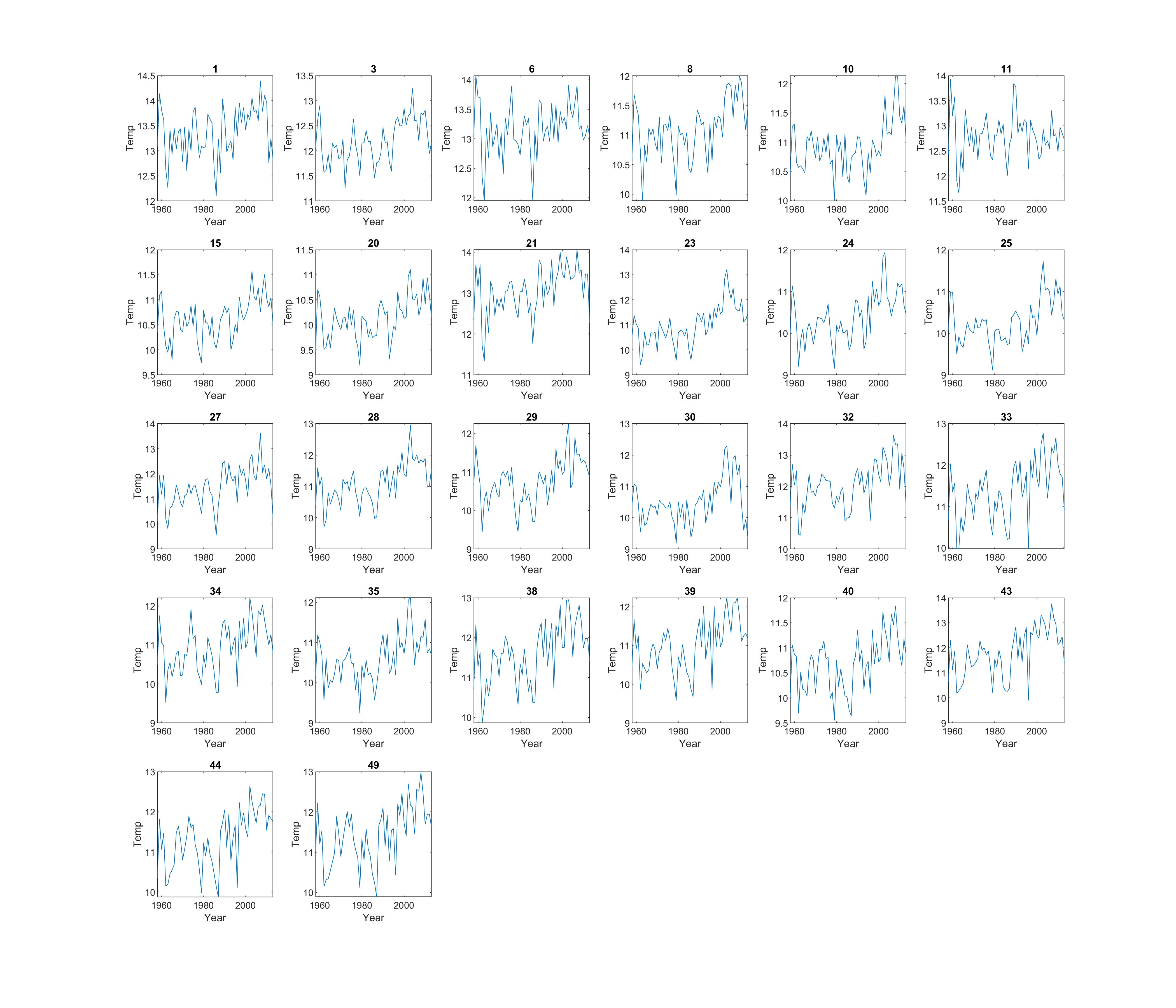

Supplement: S6 Fig — Plots numbered as in S4 Fig. (JPG) [file pcbi.1006744.s009.jpg]

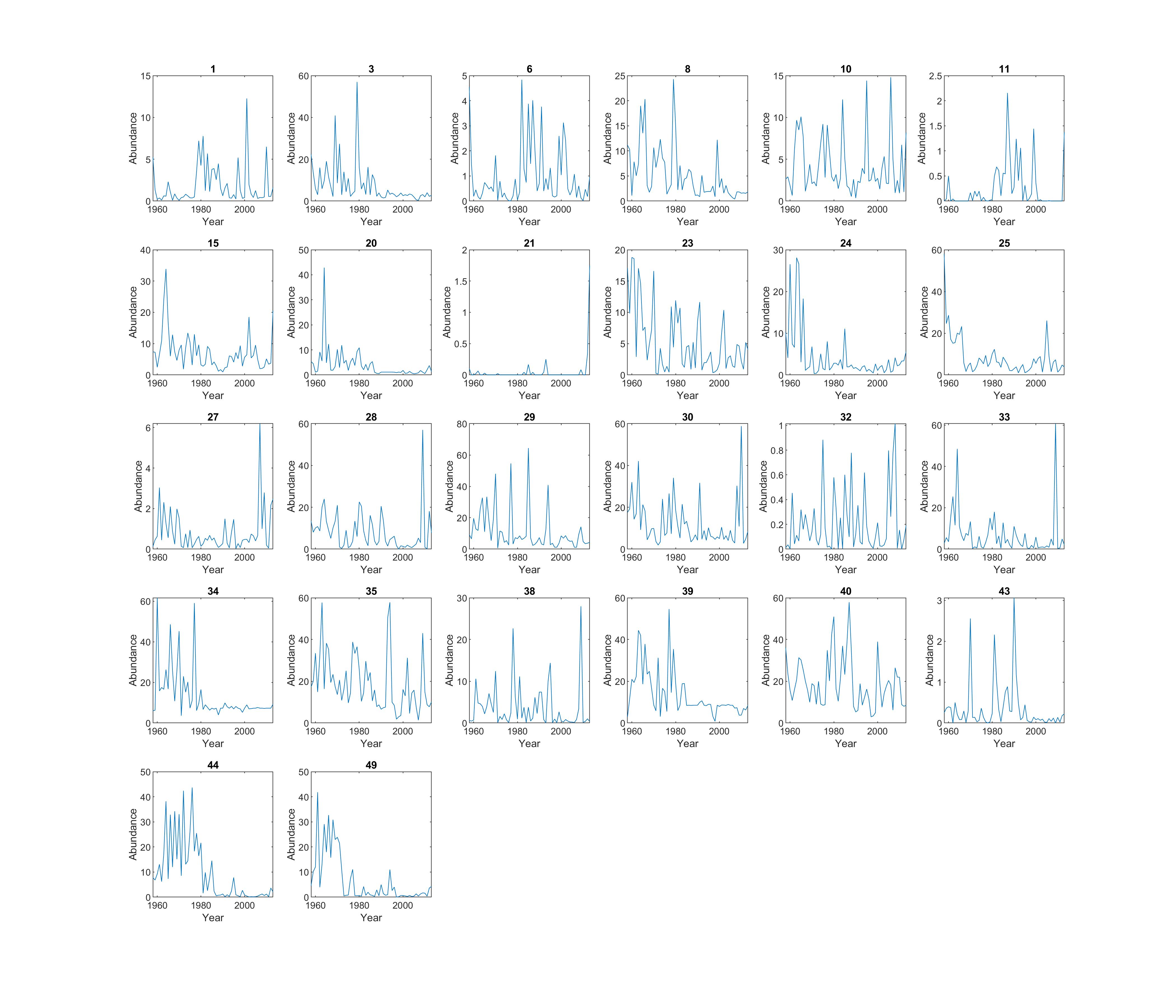

Supplement: S7 Fig — Plots numbered as in S4 Fig. (JPG) [file pcbi.1006744.s010.jpg]

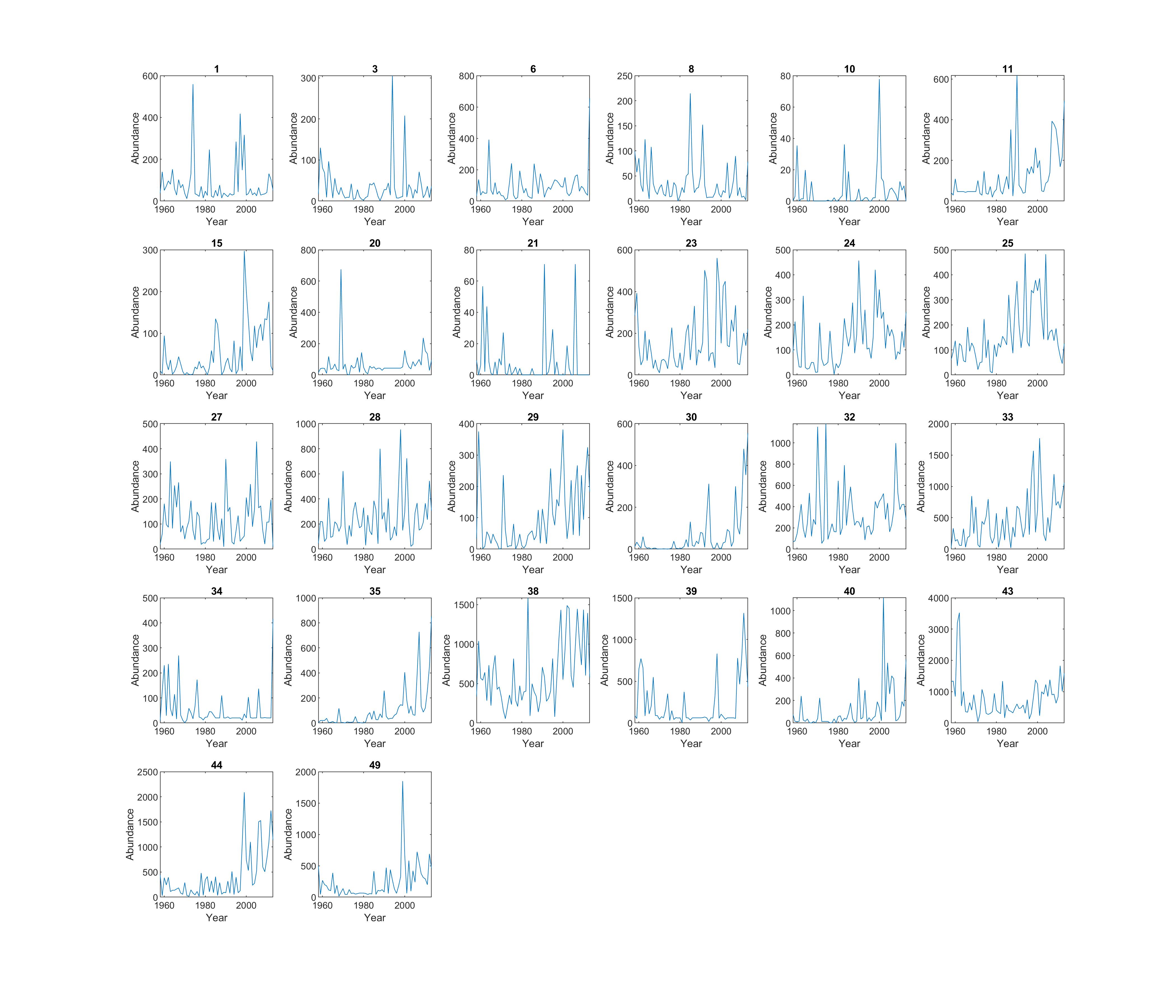

Supplement: S8 Fig — Plots numbered as in S4 Fig. (JPG) [file pcbi.1006744.s011.jpg]

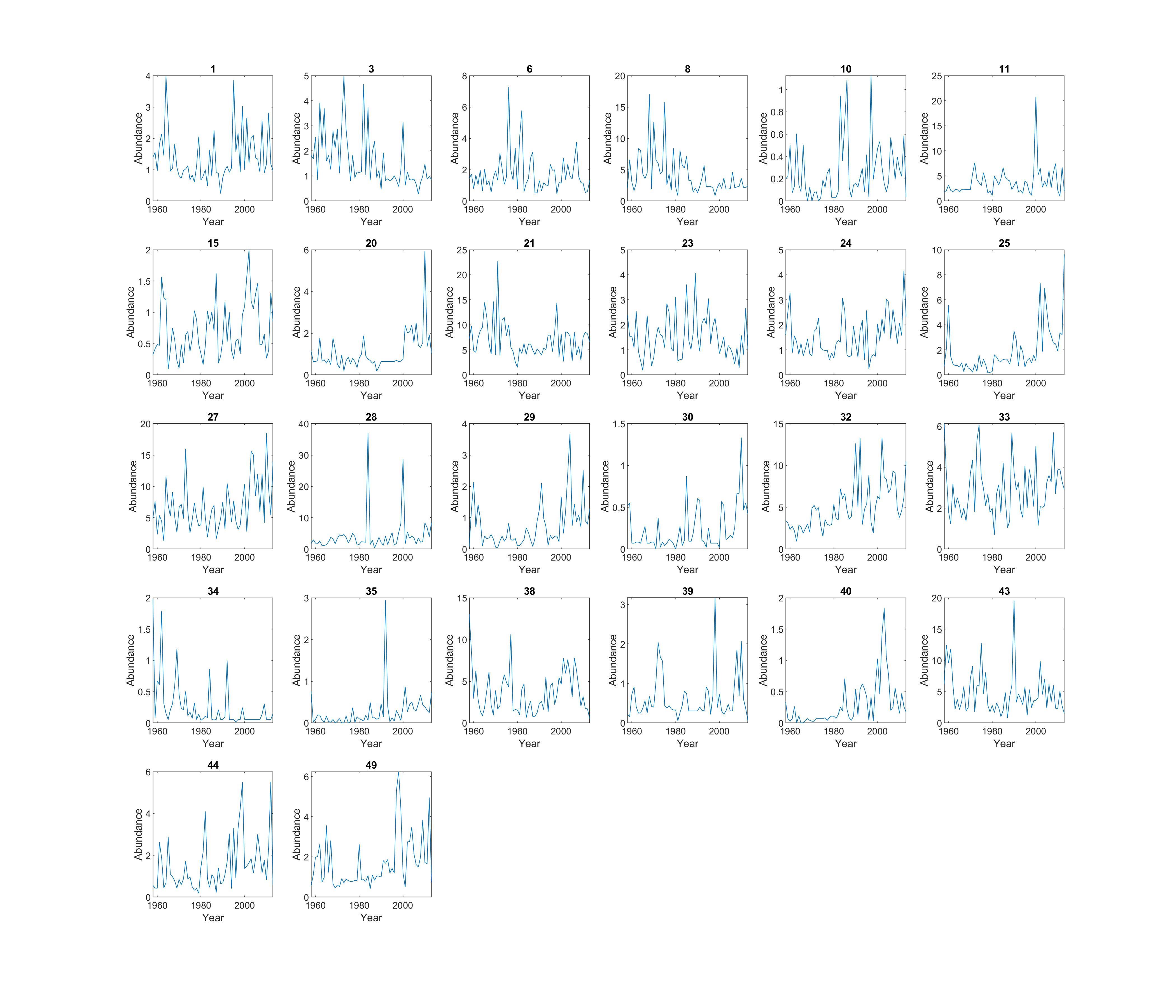

Supplement: S9 Fig — Plots numbered as in S4 Fig. (JPG) [file pcbi.1006744.s012.jpg]

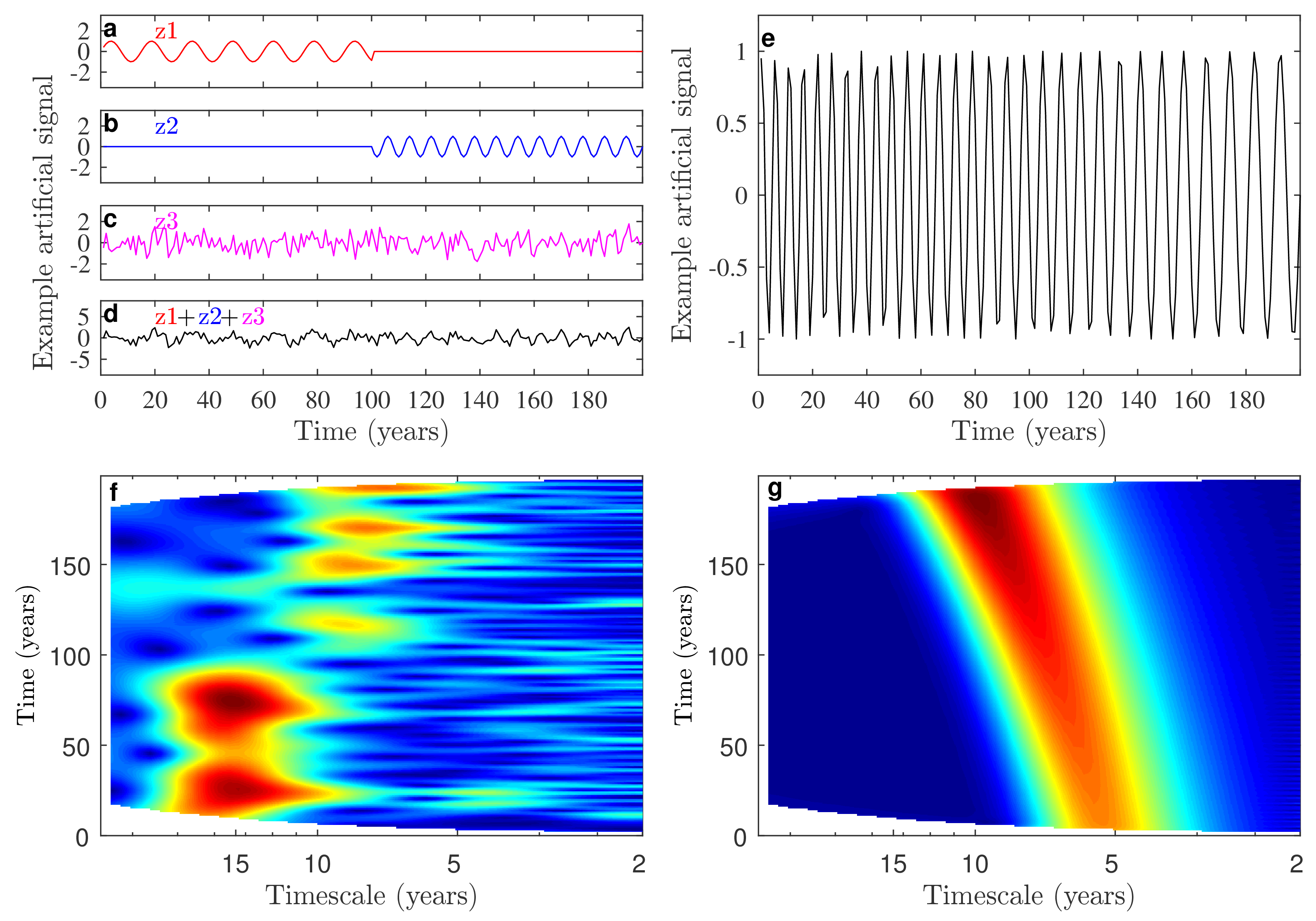

Supplement: S10 Fig — The time series of panel d was the sum of: 1) a sine wave of amplitude 1 and period 15 that operated for the first half of the time series (a); 2) a sine wave of amplitude 1 and period 8 that operated for the second half of the time series (b); and 3) normally distributed white noise of standard deviation 0.5 (c). Although periodicities in (d) and changes therein are difficult to detect by eye with any certainty, the magnitude of the wavelet transform (f; Appendix S5 in S1 Text) reveals them clearly. The time series of (e) changes period gradually from 5 to 10. The magnitude of the wavelet transform (g) shows the change. (PNG) [file pcbi.1006744.s013.png]

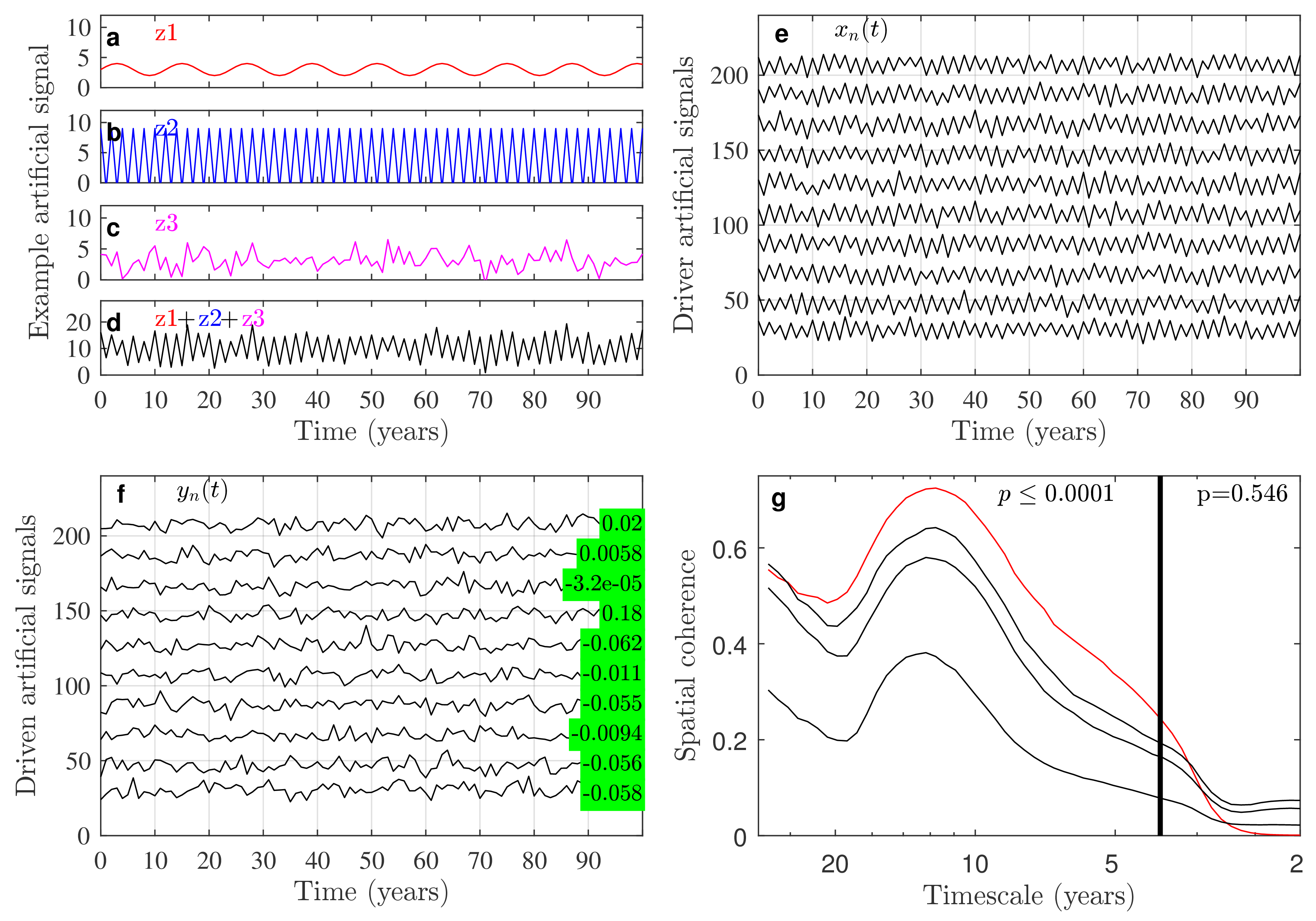

Supplement: S11 Fig — This figure was adapted with only minor changes from supplementary figure 5 of [7]. The time series of panel e were used as drivers in producing the time series of panel f and the figure shows how this relationship can be detected with the spatial coherence technique. The time series of panel e were constructed as the sum of: 1) a single common signal of amplitude 1 and period 12 years (a); 2) a single common signal of amplitude 5 and period 2 years (b); and 3) normally distributed white noise of standard deviation 1.5, independently generated for each of the 10 time series (c). The time series of panel f were produced via the relationship yn(t) = (xn(t) + xn(t − 1))/2 + ϵn(t) where the ϵn(t) were independent normal random numbers of mean 0 and standard deviation 3. This transmits the period-12 component of the x signals to the y but not the period-2 component because averaging covers a whole period for that component. Correlations (f, green numbers) between xn(t) and yn(t) did not indicate any particular relationship. Correlations cannot detect the relationship between the x and y because the technique confounds phenomena occurring on different timescales. Spatial coherences revealed a highly significant relationship at periods around 12 years (g) and on average over long timescales (left p-value on panel g, long timescales defined as > 4 years) but no relationship (right p-value) for short timescales (< 4 years). The red line on g is the spatial coherence and black lines are 50th, 95th, and 99th percentiles of spatial coherences of synchrony-preserving surrogate data sets (Appendix S8 in S1 Text) appropriately representing the null hypothesis of no relationship between the x and y. See also Appendix S9 in S1 Text for a description of how the aggregate long- and short-timescale p-values were computed. (PNG) [file pcbi.1006744.s014.png]

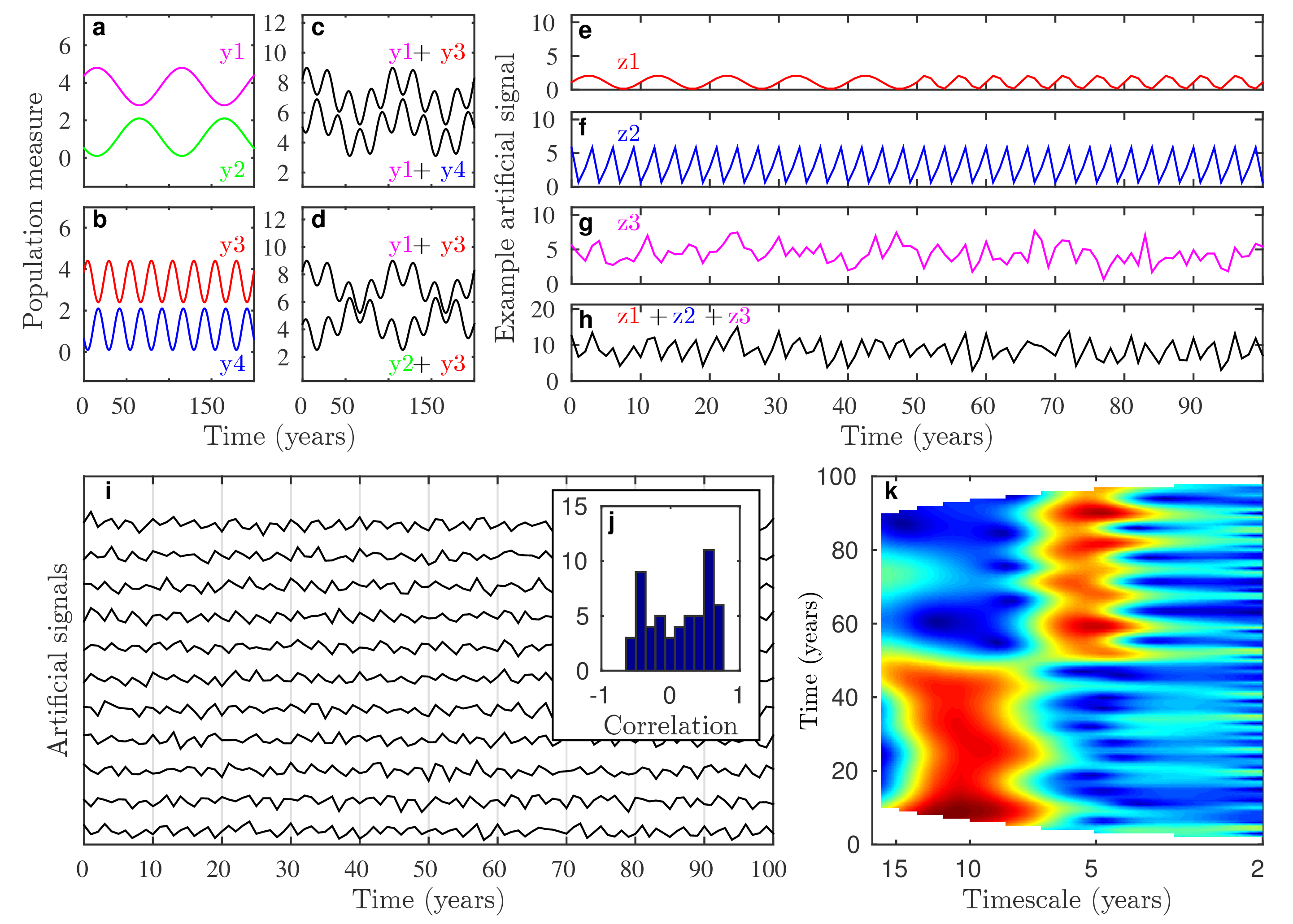

Supplement: S12 Fig — This figure was taken without change from [7]. Panels a-d show the principle of how synchrony can differ for different timescales of dynamics. Time series y1 and y2 (a) are exactly anti-correlated (out of phase), as are y3 and y4 (b). Combining y1 with y3 and y1 with y4 gives two time series (c) which are synchronized on long timescales, but anti-synchronized on short timescales. The reverse is also possible (d). This timescale-specific structure of synchrony cannot be detected with correlation coefficients, which are 0 for both c and d, because contributions from different timescales cancel. In practice, real population and environmental signals are broadband, and exact cancelation is unlikely, but asynchrony at some frequencies can strongly conceal important synchrony at other frequencies. Panels e-k demonstrate this concealment, using artificial data, and also show how the wavelet mean field detects time- and timescale-specific synchrony. Each of 11 artificial time series were constructed as the sum of: 1) a single common signal of amplitude 1 that changes its oscillatory period at t = 50 from 10 years to 5 years (e); 2) oscillations of amplitude 3 that have the same oscillatory period (3 years), but random and independent phases in each of the 11 constructed time series (f); and 3) white noise of standard deviation 1.5, again independently generated for each of the 11 time series (g). Synchrony in the resulting time series cannot be visually detected (i), nor is it readily apparent by examining the 55 pairwise correlation coefficients between time series (j), which spanned a wide range of values including 0. But the wavelet mean field magnitude (k) showed clear color bands at 10-year period for t < 50 and 5-year period for t > 50. The wavelet mean field magnitude displays strength of synchrony as a function of timescale of dynamics and time, here with red indicating synchrony and blue asynchrony. Wavelet phasor mean fields provide plots similar to (k) but with v [file pcbi.1006744.s015.png]

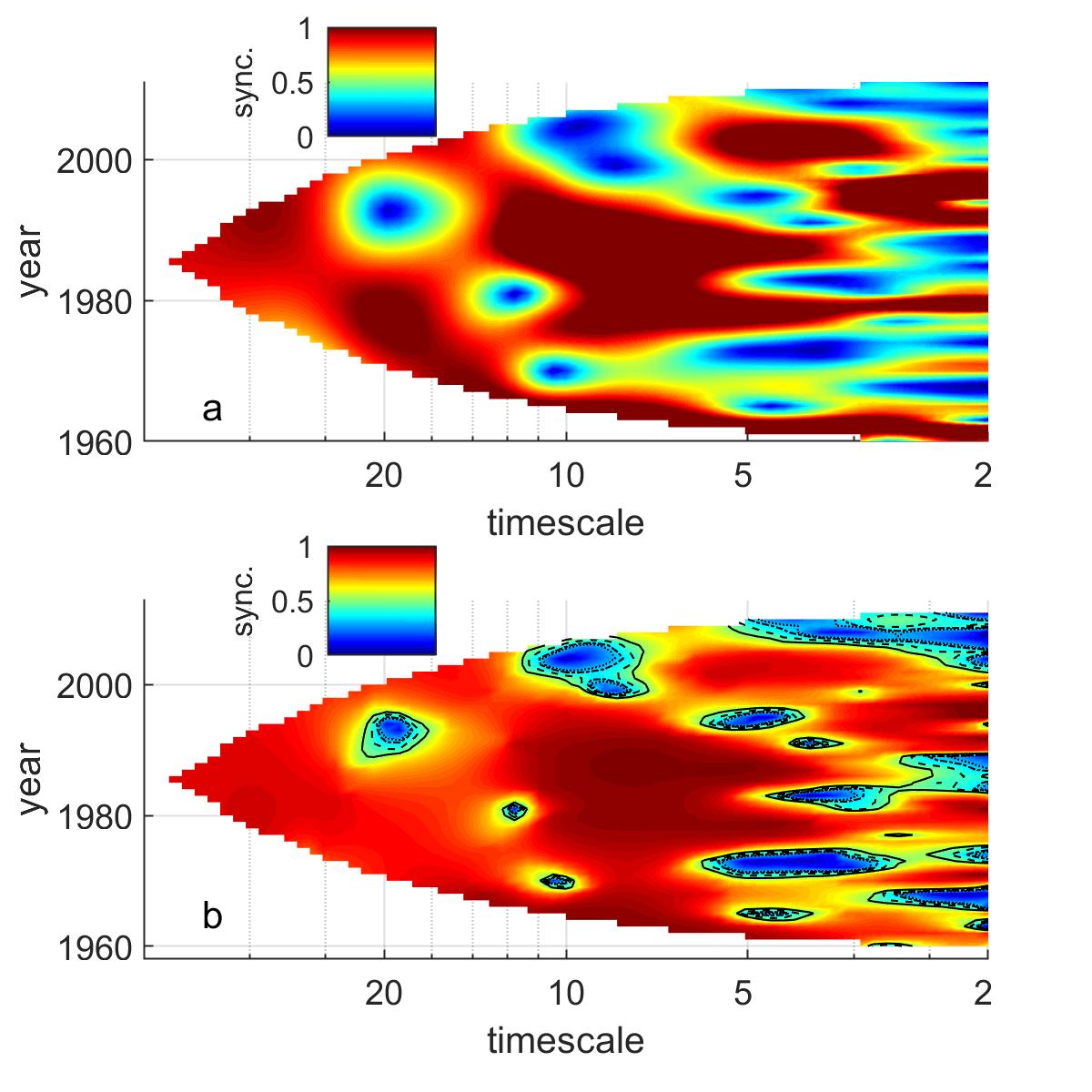

Supplement: S13 Fig — Statistical significance thresholds on the WPMFM are plotted as contours showing actual phase agreement between locations greater than the 90th, 95th, 99th and 99.9th percentile of a distribution of unsynchronized unit phasors (dotted, dash-dotted, dashed and line contours respectively). (JPG) [file pcbi.1006744.s016.jpg]

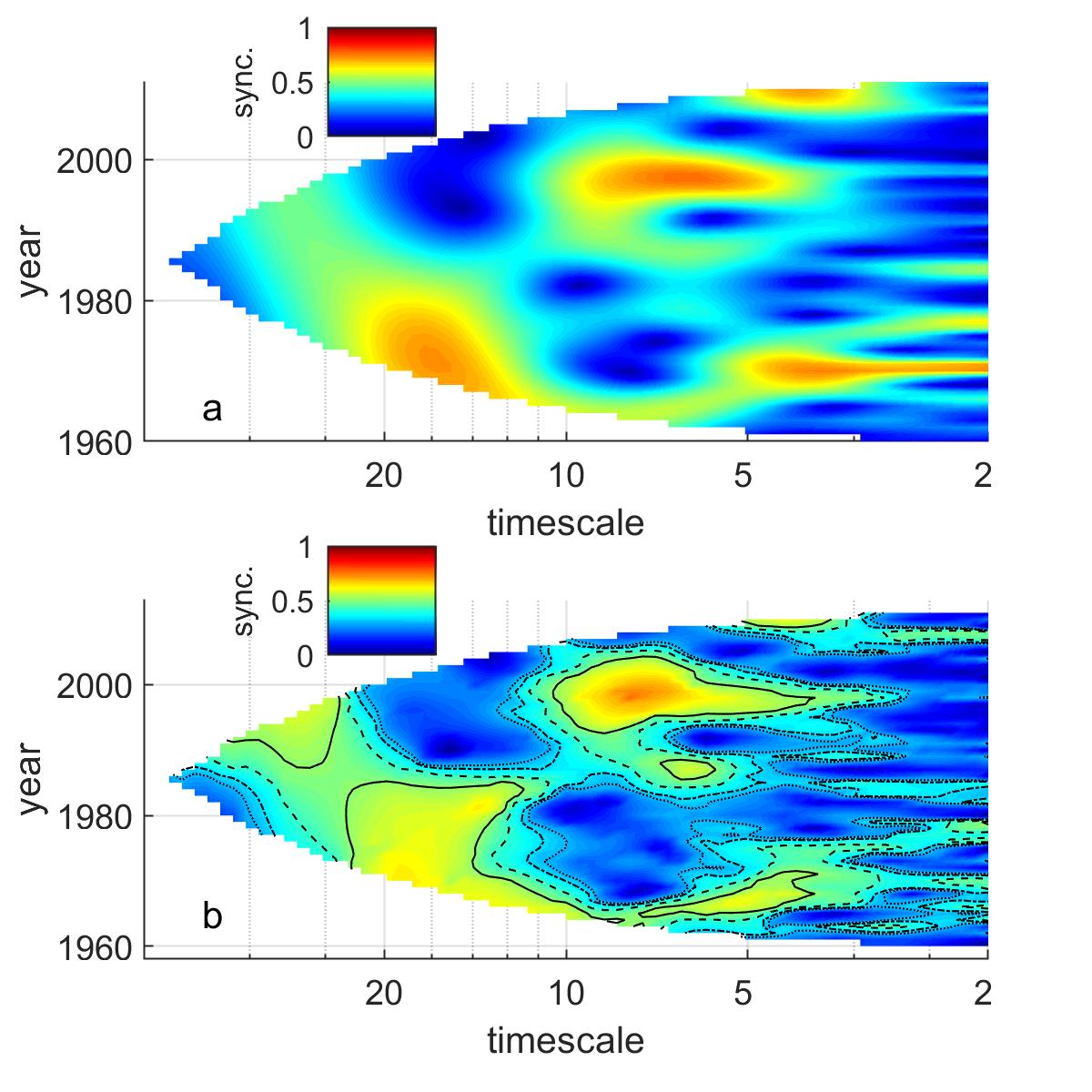

Supplement: S14 Fig — Statistical significance thresholds on the WPMFM are plotted as contours showing actual phase agreement between locations greater than the 90th, 95th, 99th and 99.9th percentile of a distribution of unsynchronized unit phasors (dotted, dash-dotted, dashed and line contours respectively). (JPG) [file pcbi.1006744.s017.jpg]

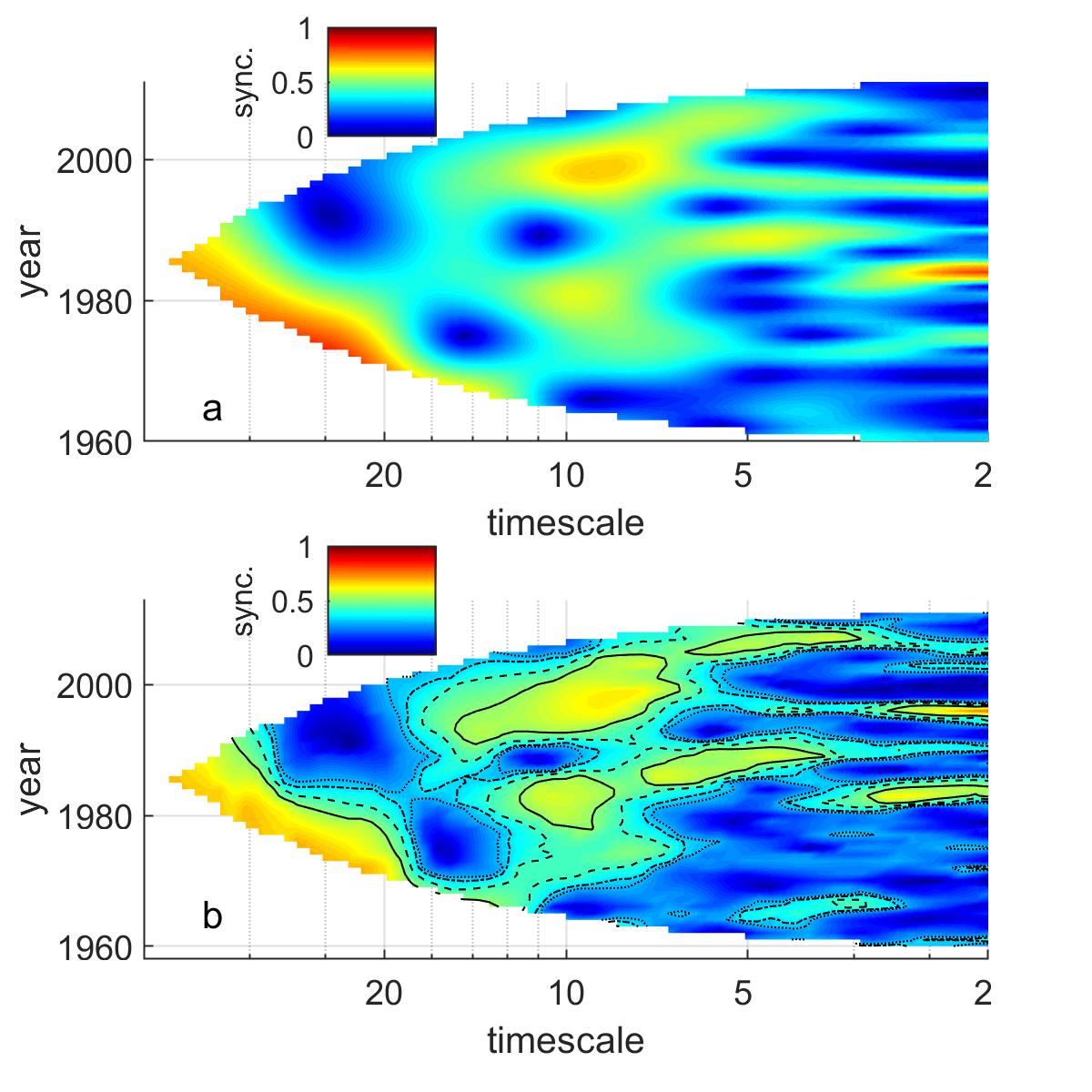

Supplement: S15 Fig — Statistical significance thresholds on the WPMFM are plotted as contours showing actual phase agreement between locations greater than the 90th, 95th, 99th and 99.9th percentile of a distribution of unsynchronized unit phasors (dotted, dash-dotted, dashed and line contours respectively). (JPG) [file pcbi.1006744.s018.jpg]

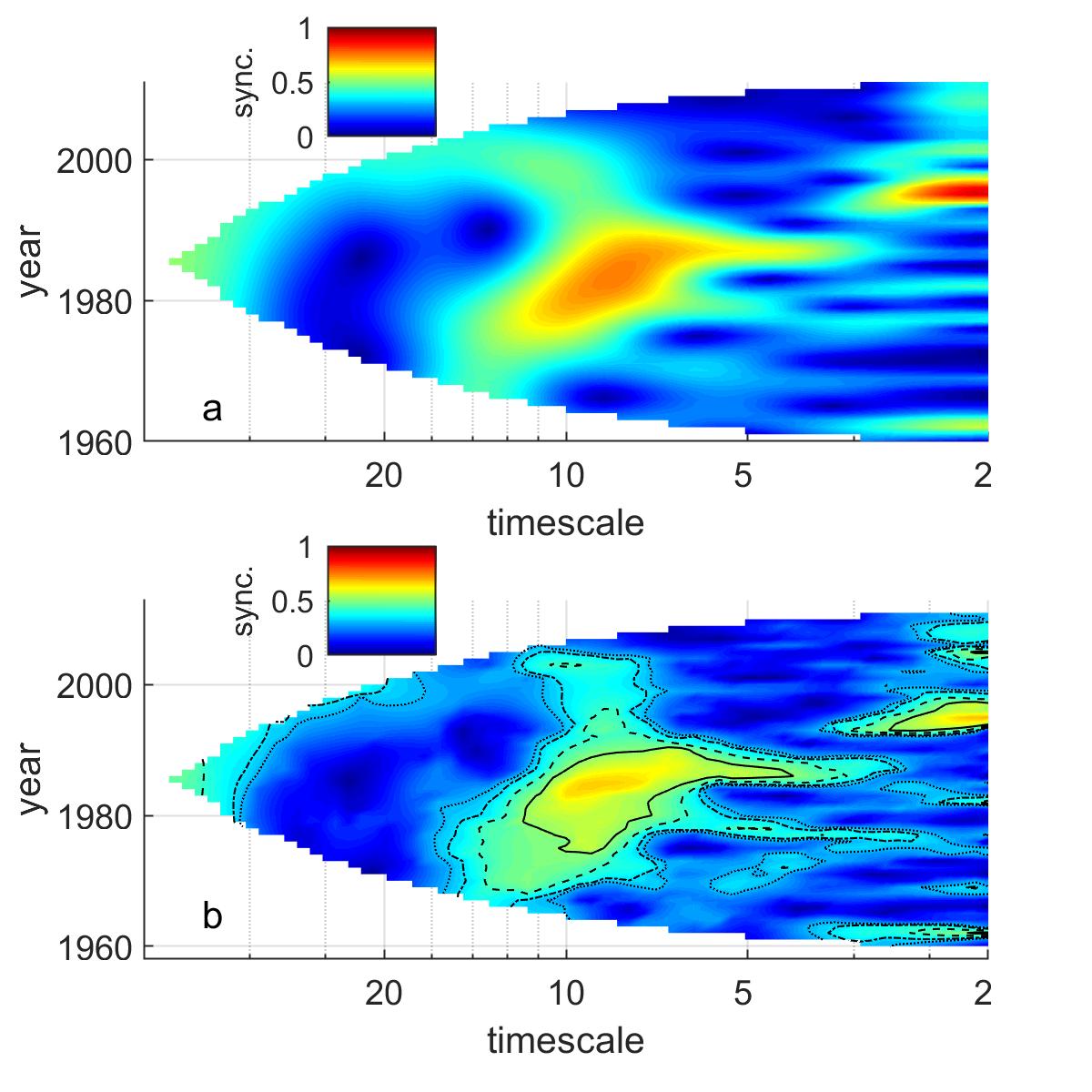

Supplement: S16 Fig — Statistical significance thresholds on the WPMFM are plotted as contours showing actual phase agreement between locations greater than the 90th, 95th, 99th and 99.9th percentile of a distribution of unsynchronized unit phasors (dotted, dash-dotted, dashed and line contours respectively). (JPG) [file pcbi.1006744.s019.jpg]
